# Greener deprotection pathways: microwave assisted aqueous deprotection of *N*-Boc amines

**DOI:** 10.1039/d6ra08533d

**Published:** 2026-10-26

**Authors:** José Vidal-Núñez, Sebastián Morales-Guerrero, Lars Ratjen, Eduardo Schott, Flavia C. Zacconi

**Affiliations:** a Departamento de Química Orgánica, Facultad de Química y de Farmacia, Pontificia Universidad Católica de Chile Vicuña Mackenna 4860 Santiago 7820436 Chile fzacconi@uc.cl; b Center for Green Chemistry and Green Engineering, School of the Environment, Yale University New Haven Connecticut 06511 USA; c Departamento de Química Inorgánica, Facultad de Química y de Farmacia, CE-UC, CIEN-UC, Pontificia Universidad Católica de Chile Avenida Vicuña Mackenna 4860 Santiago Chile; d Institute for Biological and Medical Engineering, Schools of Engineering, Medicine and Biological Sciences, Pontificia Universidad Católica de Chile Av. Vicuña Mackenna 4860, Macul Santiago 7820436 Chile

## Abstract

The development of sustainable deprotection strategies is crucial for reducing the environmental impact of multi-step syntheses in pharmaceutical and fine chemical production. In this work, we present a rapid microwave-assisted protocol for the deprotection of *N*-Boc-protected amines using water as the sole solvent, without corrosive acids, organic solvents, or catalysts. The method enables quantitative deprotection of a wide range of substrates in minutes, significantly reducing reaction times compared to conventional methods and improving the overall sustainability of the process. Density functional theory calculations were performed to understand the reaction mechanism and thermodynamic characteristics of the transformation in aqueous media. The protocol demonstrates broad functional-group tolerance and substrate compatibility, offering a simpler, more environmentally friendly alternative to *N*-Boc deprotection in medicinal chemistry and related synthetic applications.

## Abstract 2

Microwave-assisted *N*-Boc deprotection in water provides a rapid, catalyst-free alternative to conventional acidic protocols, affording a broad range of amines in high yields within short reaction times.

## Graphical abstract

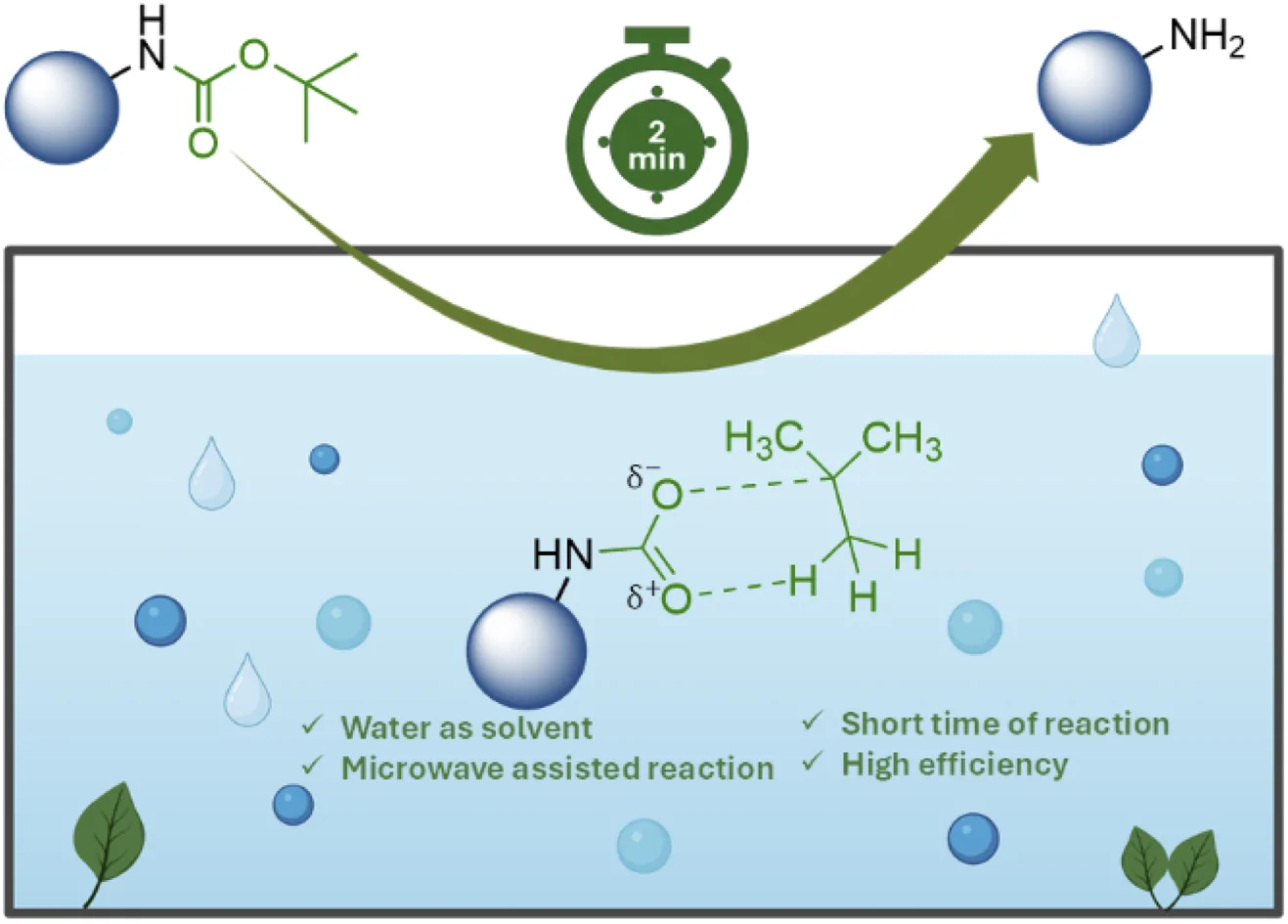

## 1. Introduction

In recent decades, organic chemistry has advanced significantly towards developing greener and more sustainable methodologies, strongly emphasizing synthetic procedures that minimize the environmental impact associated with chemical products.^1–4^ In the context of complex syntheses, the protection and deprotection of functional groups remains a critical.^5^ Despite efforts to avoid protection groups completely,^6,7^ they remain irreplaceable in chemical synthesis, since they allow for minimizing undesired reaction pathways, which hamper overall yields.^8^

The amino group plays a central role in organic synthesis and in the chemical industry. Amines serve as essential building blocks in the synthesis of polyamides, polyureas, and polyurethanes, materials widely utilized in the automotive, aerospace, and construction sectors.^9–11^ Moreover, the amine functionality is a cornerstone of numerous bioactive molecules, including drugs and natural products, making it essential for the synthesis of pharmaceutical ingredients and key intermediates in drug development.^12,13^ Due to its reactivity, the protection and deprotection of this functional group remain areas of utmost interest. To illustrate this, protecting amino groups is fundamental in synthesizing nitrogen-containing heterocycles, a shared structural motif in over 75% of FDA-approved drugs.^14^

Recent analyses reveal that up to 25% of all reactions performed in drug synthesis involve a protective group manipulation, with amine protections/deprotections representing a significant fraction.^15,16^ These operations are commonly employed in the synthesis of several pharmaceutically relevant compounds, including glipizide,^17^ used for the treatment of type 2 diabetes; sertraline,^18^ an antidepressant widely prescribed for mood disorders; and domperidone,^19^ used in the management of gastrointestinal conditions (Fig. 1). In all these cases, the removal of protecting groups is critical, underscoring the need to optimize these transformations towards more sustainable, efficient protocols.

**Fig. 1:**
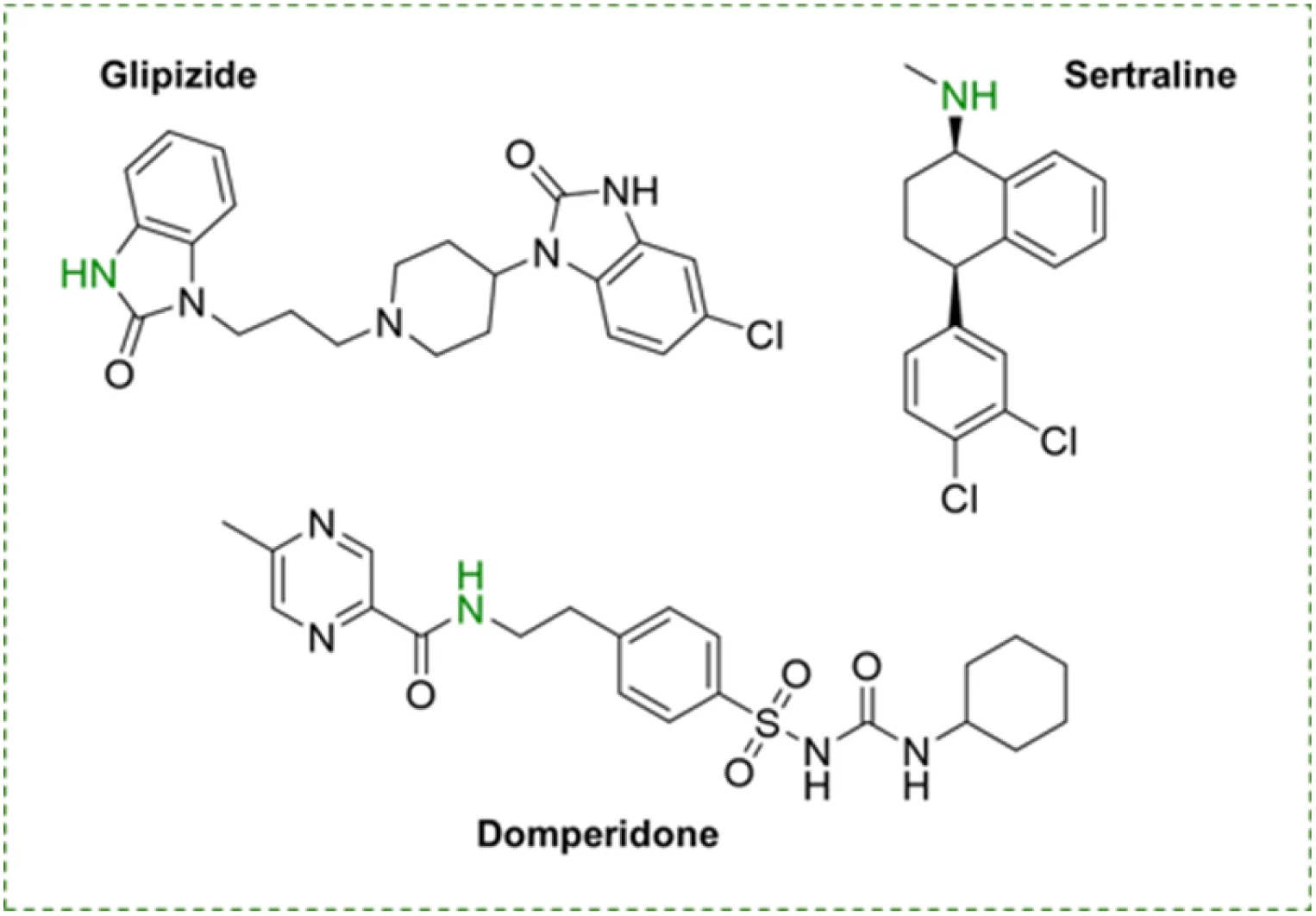
Commercial drugs synthesized using intermediates containing *N*-Boc-protected amines (relevant N-atoms highlighted in green).

The most widely used protecting group for nitrogen atoms is the *tert*-butoxycarbonyl (Boc) due to its high stability under diverse reaction conditions^20^ and ease of selective removal in orthogonal protection strategies.^21,22^ Thus, the deprotection of *N*-Boc groups remains a frequent and often critical step in pharmaceutical development.^21^ The limitations of *N*-Boc deprotection methodologies frequently result in difficult product isolation and purification, especially in multistep syntheses.^14^ Conventional *N*-Boc protection/deprotection methods rely on toxic reagents and solvents, excessive use of corrosive mineral acids, moderate yields, expensive catalysts, long reaction times, energy-intensive reflux conditions, and complex protocols.^23–32^

Deprotection methodologies usually imply superstoichometric quantities of organic or inorganic acids. The most common deprotection approach is mediated by trifluoracetic acid (TFA) in dichloromethane (DCM). TFA, a polyfluoroalkyl substance (PFAS), is particularly problematic due to its associated environmental and health concerns.^33,34^ Other common methodologies feature HCl in organic solvents such as tetrahydrofuran (THF), acetonitrile (MeCN), 1,4-dioxane, and toluene. In the quest for more environmentally friendly methodologies, thermal deprotections have emerged as a powerful alternative, as they typically do not require additional reagents. However, these reactions typically require high temperatures (>150 °C) and extended reflux times, which are especially challenging for more complex structural motifs.^35–37^ Also, the overall environmental benefits of employing water as a solvent may be counterbalanced by higher energy consumption and operational costs.^38^ The high utility and wide prevalence of the *N*-Boc protecting emphasize the dire need for more sustainable methodologies (see Fig. 2 for details).

**Fig. 2:**
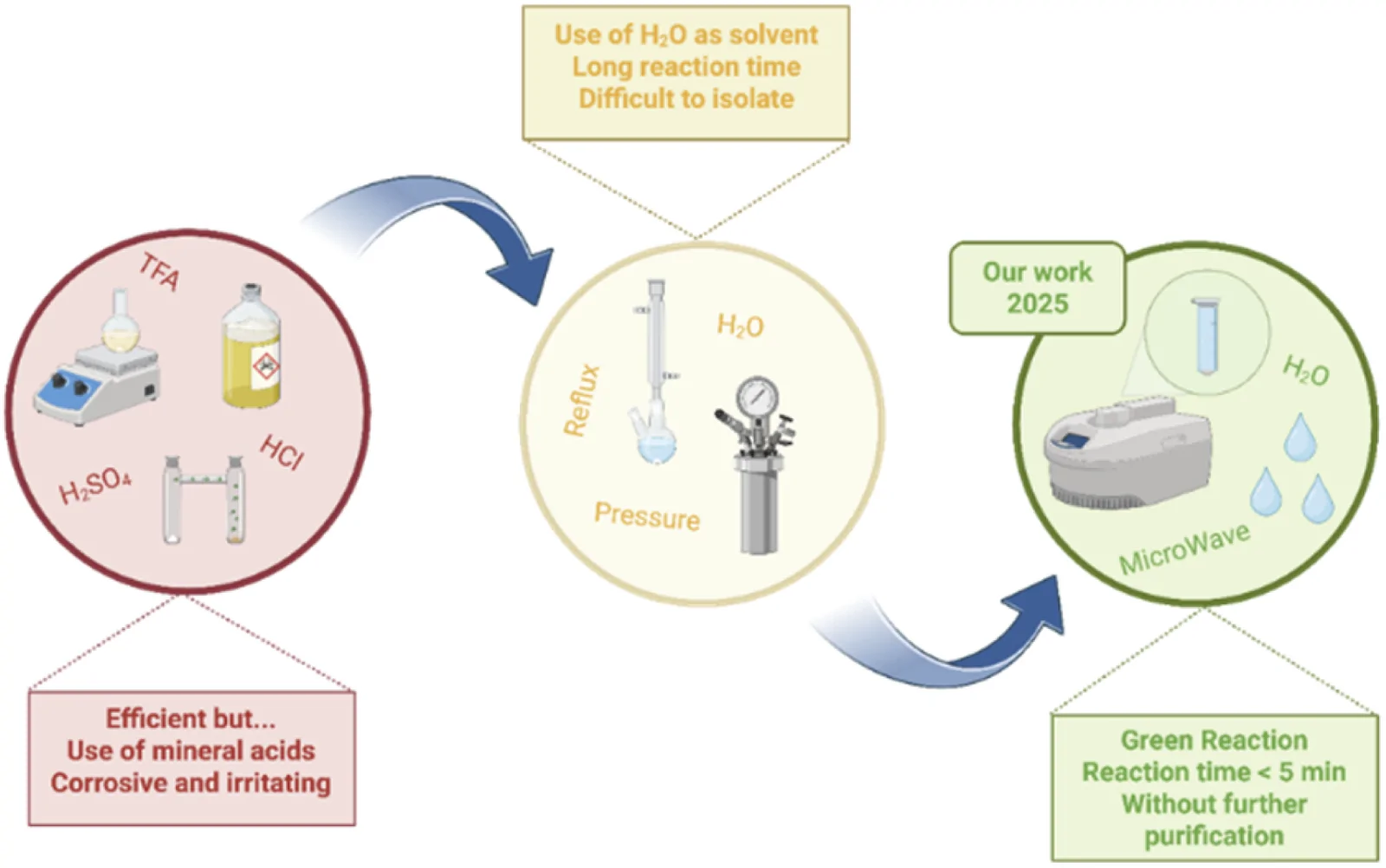
Schematic representation of the methodological evolution of *N*-Boc deprotection, from traditional acid-mediated protocols to sustainable strategies using water and microwave irradiation.

Here, we report a microwave-assisted *N*-Boc deprotection using water as the sole solvent, without additional reagents. Microwave irradiation significantly reduces reaction times, from up to 14 hours under conventional thermal conditions to 2–30 minutes, thereby drastically improving the efficiency and synthetic feasibility of deprotection and offering a concrete contribution towards more sustainable and greener synthetic protocols. Furthermore, we evaluate the developed method using established green chemistry metrics, allowing for a systematic comparison with conventional *N*-Boc deprotection procedures. The evaluation of metrics for synthetic methods is increasingly important, as it enables the alignment of the chemical enterprise with key objectives of the United Nations Sustainable Development Goals (SDGs), as well as the planetary boundaries framework proposed by Rockström,^39,40^ addressing chemical pollution and resource depletion. In celebration of the first anniversary of the Stockholm Declaration on Chemistry for the Future,^41^ prioritizing the design of molecules and materials that are inherently sustainable, safe, efficient, and environmentally responsible, this report is especially timely and relevant.

## 2. Experimental details

Commercially available reagents, chemicals, *N*-Boc-protected substrates, and deprotected amine standards were purchased from Sigma-Aldrich, Merck, or Ambeed and used as received without further purification, unless otherwise stated. According to the supplier specifications or certificates of analysis, the declared purities of the commercial reagents and substrates were typically 98–99%. All solvents used for reactions, extraction, washing, and purification were HPLC grade, except for the deuterated solvents used for NMR analysis, which were NMR grade. HPLC-grade water was used as the reaction medium for the aqueous microwave-assisted *N*-Boc deprotection reactions. Compounds 13, 14, 22, and 24 were synthesized according to previously reported procedures^42,43^ and characterized as described in the Experimental section.

Microwave-assisted reactions were performed in a Monowave 400 microwave synthesis reactor (Anton Paar, Graz, Austria). Reaction progress was monitored by thin-layer chromatography (TLC) on silica gel plates (Merck 60 F254) with solvent systems optimized for each substrate.

Nuclear magnetic resonance (NMR) spectra were recorded on Bruker Avance 400 instruments operating at 400 MHz for ^1^H NMR and 100 MHz for ^13^C NMR (Bruker, Billerica, MA, USA). CDCl_3_ was used as the solvent unless otherwise specified. Chemical shifts (*δ*) are reported in parts per million (ppm) relative to tetramethylsilane (TMS) as the internal standard.

### 2.1 Computational details

All geometry optimizations were conducted using density functional theory (DFT) with the B3LYP exchange–correlation functional and the def2-TZP basis set, as implemented in the ORCA 6.0 software package.^44,45^ To improve the accuracy of the results, Grimme's D4 empirical dispersion correction was included,^46^ accounting for long-range dispersion interactions and providing corrections to the total energy, energy gradients, and vibrational frequencies.

Frequency calculations were performed at the same level of theory to confirm that all optimized structures correspond to the true minimum on the potential energy surface, as indicated by the absence of imaginary frequencies. The diagonalization of the Hessian matrix yielded the vibrational eigenfrequencies and corresponding normal modes, which describe the harmonic motion of the molecular nuclei. All computational protocols and input files are available in the SI.

Molecular structures were initially constructed using Avogadro software (version 1.2.0),^47^ followed by a preliminary optimization with the integrated “optimize geometry” extension. Subsequent optimizations were performed using density functional theory (DFT) methods, employing the selected functional and the Def2-TZP basis set with D4 dispersion corrections.^48^ Optimized geometries were confirmed as true minima by vibrational frequency analysis, ensuring the absence of imaginary frequencies.

Reaction pathways were explored using the Nudged Elastic Band (NEB) method, connecting optimized reactant and product geometries.^49^ The minimum energy pathways obtained were further refined through transition state (TS) optimizations. The identity of the transition states was confirmed by frequency analysis, which revealed a single imaginary frequency along the reaction coordinate.

### 2.2 Experimental details

#### 2.2.1 General procedure for *N*-Boc deprotection

For the deprotection of *N*-Boc derivatives, 1.0 mmol of substrate was placed in a G10 microwave reaction tube with 2.0 mL of HPLC-grade water. The tube was sealed and heated in an Anton Paar Monowave 400 microwave reactor at a target temperature of 140 °C for 2 min. The reactor has a maximum microwave output power of 850 W and automatically adjusts the applied power during operation. Upon completion, the reaction mixture was cooled to room temperature, diluted with 20.0 mL of ethanol, and subsequently evaporated under reduced pressure (70 mbar, 40 °C) to yield the corresponding free amine. The isolated products were obtained quantitatively without further purification.

## 3. Result and discussion

### 3.1 Optimization of the *N-*Boc deprotection methodology

In pursuit of more sustainable methodologies for *N*-Boc deprotection, we initially evaluated conditions adapted from Ly *et al.*^24^ using *tert*-butyl (4-hydroxyphenyl)carbamate as a model substrate (Table 1). The initial experiments were performed in dichloromethane (DCM) using 85% phosphoric acid (H_3_PO_4_, 8–30 equiv.) under microwave irradiation at 30 °C and 50 °C. However, complete conversion was not achieved within 1 h. This result was attributed, at least in part, to the heterogeneous nature of the reaction medium, where limited contact between the organic substrate and the aqueous phosphoric acid phase may have reduced the efficiency of the deprotection process.

**Table 1:** Summary of the optimization steps for the microwave-assisted *N*-Boc deprotection of 16

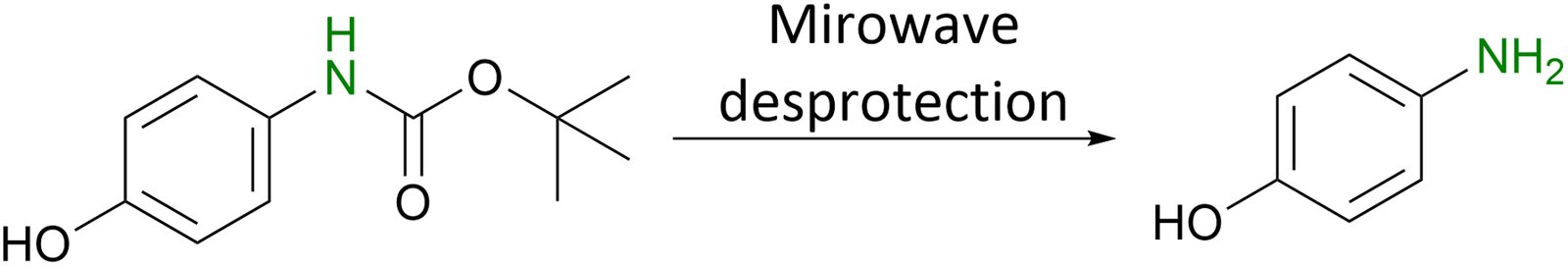			
Step	Solvent system	Conditions	Outcome
1	DCM	H_3_PO_4_ (8–30 equiv.), 30–50 °C, MW, 1 h	Partial conversion (48%)
2	THF	H_3_PO_4_, (18 equiv.), 50 °C, MW	Partial conversion (40%)
3	2-MeTHF	H_3_PO_4_, 50 °C, MW	Partial conversion (50%)
4	Dioxane	H_3_PO_4_, (18 equiv.), 50 °C, MW	Partial conversion (15%)
5	MeCN	H_3_PO_4_ (18 equiv.), 50 °C, MW, 15 min	Full conversion, difficult workup (>98%)
6	MeCN/H_2_O (1 : 1)	H_3_PO_4_, (18 equiv.), 50 °C, MW	High conversion, product losses during workup (>90%)
7	H_2_O	MW optimization	Best green reaction medium (>99%)

To overcome this limitation, a solvent screening was conducted to improve substrate–acid contact and identify a more efficient and sustainable reaction medium. Solvents with different polarities and miscibility profiles, including tetrahydrofuran (THF), 2-methyltetrahydrofuran (2-MeTHF), acetonitrile (MeCN), 1,4-dioxane, and water, were evaluated under comparable microwave-assisted conditions. THF, 2-MeTHF, and 1,4-dioxane afforded only partial conversion, whereas both acetonitrile and water resulted in complete *N*-Boc deprotection, with isolated yields exceeding 90%. However, the reaction times differed considerably since complete conversion required 15–30 min in acetonitrile and approximately 45 min in water.

The higher conversions observed in polar media can be attributed to improved stabilization of polar intermediates or transition states involved in the acid-promoted cleavage of the *N*-Boc group. In addition, water has been reported to promote acid–base processes at elevated temperatures, partly through changes in its ionization behavior under high-temperature conditions.^50^ These considerations supported the further evaluation of both acetonitrile- and water-based systems.

Because acetonitrile afforded complete conversion with shorter reaction times, the initial optimization was focused on this solvent. Microwave-assisted deprotection of carbamate 16 in acetonitrile at 50 °C using 18 equiv. of H_3_PO_4_ afforded the deprotected product in 98% yield after 15 min. Nevertheless, the isolation of the highly polar product was operationally demanding and required extensive use of organic solvents during extraction and purification. To address this limitation, a 1 : 1 v/v ACN/H_2_O mixture was subsequently evaluated. Although high substrate conversion was maintained at 50 °C, product recovery remained inefficient because significant losses occurred during the aqueous workup. On this basis, water was reconsidered as the sole reaction medium and selected for systematic optimization. This choice was based not only on substrate conversion but also on operational simplicity, reduced reliance on organic solvents, compatibility with microwave heating, and its potential to facilitate deprotection under high-temperature aqueous conditions. Accordingly, the effects of temperature and reaction time under microwave irradiation were investigated, and the results are summarized in Table 2.

**Table 2:** Optimization of the deprotection reaction using H_2_O and microwave irradiation

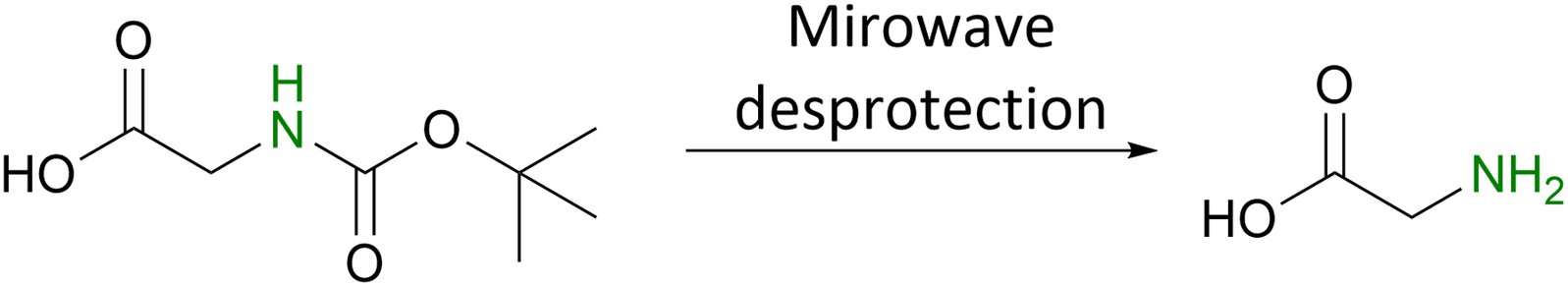						
Entry	Substrate (mg)	Substrate (mmol)	H_2_O volume (mL)	Temperature (°C)	Time (min)	Conversion^a^ (%)
1	100	0.52	2.0	100	2.0	53
2	100	0.52	2.0	100	4.0	67
3	100	0.52	2.0	100	8.0	83
4	100	0.52	2.0	120	8.0	>99
5	100	0.52	2.0	120	6.0	>99
6	100	0.52	2.0	120	3.0	>99
7	100	0.52	2.0	130	2.0	>99
8	100	0.52	2.0	140	1.5	>99
9	100	0.52	2.0	140	1.0	>99
10	100	2.85	10.0	150	0.5	>99
11	500	2.85	5.0	150	0.5	>99
12	500	2.85	2.0	150	0.5	>99
13	1000	5.70	2.0	150	0.5	>99

a Conversion was calculated by dividing the methylene product integral with the sum of product and starting material methylene integrals.

To investigate the scope of the newly developed approach, microwave-assisted *N*-Boc deprotection was evaluated using a 100 mg of *N*-Boc glycine, the simplest nitrogen-protected amino acid (1). Initially, the experiments were carried out at 100 °C for 2, 4 and 8 minutes, respectively, to assess the effect of reaction time on deprotection efficiency. Conversions were determined using ^1^H-NMR (in D_2_O) by comparing the integrals of the methylene protons of the protected compound with those of the deprotected amino acid (Fig. 3). Under these conditions, conversions of 53, 67, and 83% were obtained after 2, 4, and 8 min, respectively (Table 2, entries 1–3).

**Fig. 3:**
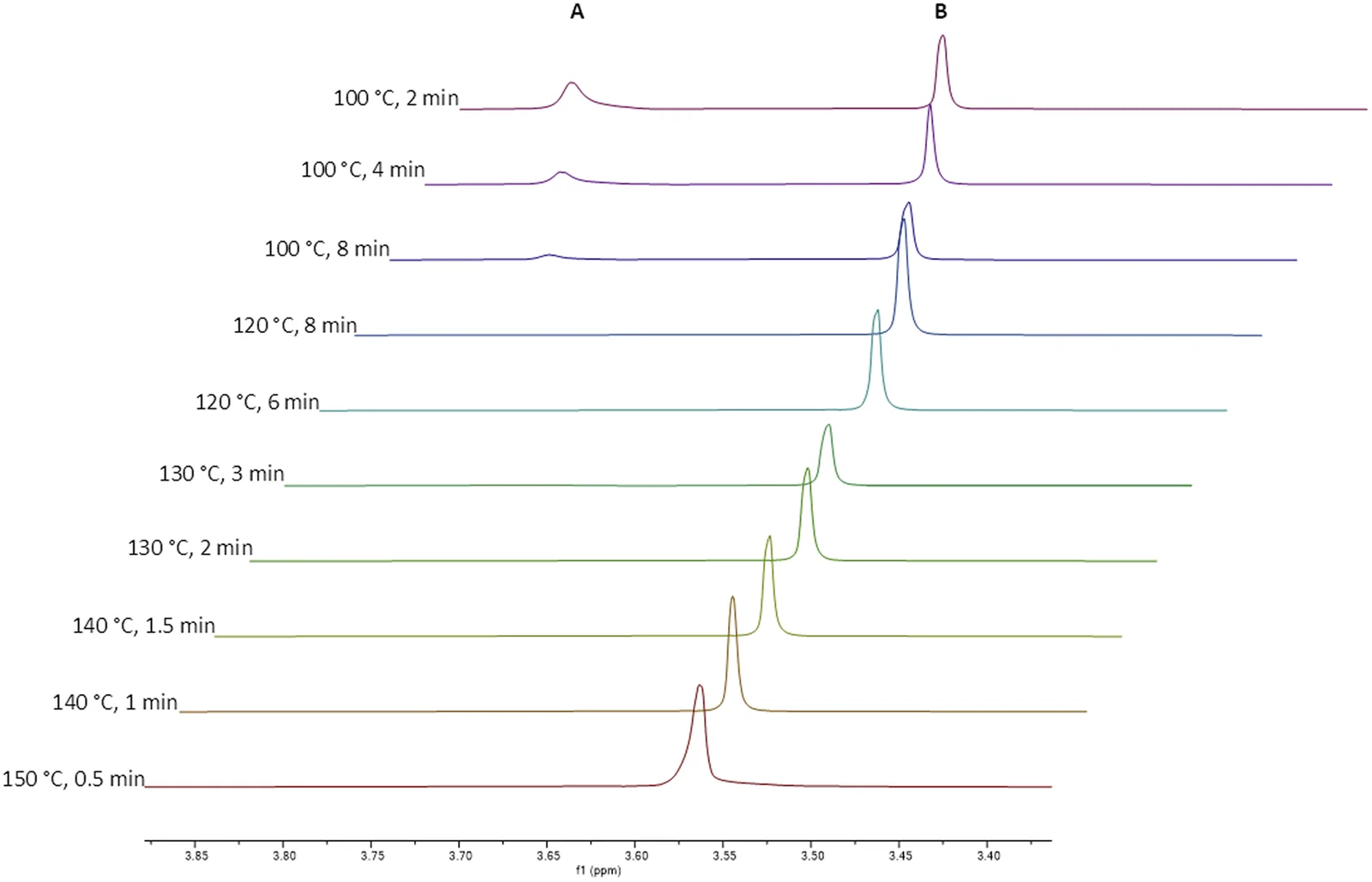
Reaction progress of *N*-Boc glycine deprotection in D_2_O monitored by ^1^H NMR spectroscopy, showing the progressive disappearance of the protected substrate signals and the formation of the deprotected glycine derivative (entry 1–10).

As shown in Table 2, entries 1–3, increasing the reaction time produced progressively smaller improvements in conversion. This trend is consistent with previous kinetic studies on thermal *N*-Boc deprotection. Liotta *et al.*,^51^ suggested that *N*-Boc deprotection at 150 °C may follow either first-order or autocatalytic kinetics, while Li *et al.*^50^ investigated *N*-Boc deprotection under continuous-flow conditions at 225 °C and described the reaction using a first-order kinetic model. In these kinetic descriptions, conversion displays a nonlinear time dependence and approaches completion asymptotically. Therefore, rather than substantially extending the reaction time once high conversion had been reached, the temperature was increased to accelerate deprotection while maintaining short processing times. In addition, recent studies on thermolytic *N*-Boc deprotection have shown that the apparent reaction order can depend considerably on the solvent, with different kinetic regimes arising from solvent–substrate interactions.^52^

Increasing the temperature to 120 °C afforded quantitative conversion after 8 min (entry 4), confirming that higher temperatures markedly accelerate the *N*-Boc deprotection and reduce the time required for complete conversion. The reaction time was subsequently shortened to 6 and 3 min (entries 5 and 6), while quantitative conversion was maintained. Because glycine was obtained quantitatively under these conditions, the temperature was further increased to identify the minimum reaction time required for complete deprotection (entries 6–10). Ultimately, quantitative conversion was achieved within only 30 s at 150 °C, compared to other thermal *N*-Boc deprotection protocols carried out under conventional reflux or in small sealed reactors.^36,51,53^ The microwave-assisted approach reported here significantly reduces reaction times, underscoring the key role of microwave irradiation.

After establishing the optimal temperature and reaction time, the effects of substrate concentration and scale-up were investigated. The reaction was first scaled up fivefold to a 0.5 g, affording glycine quantitatively without any operational issues (entry 10). The water volume was then reduced by half while maintaining complete conversion (entry 11). Subsequently, the reaction was performed at the same scale under 2.5-fold increase of concentration, again affording quantitative conversion (entry 12). Finally, the procedure was successfully scaled to a 1.0 g substrate loading, affording glycine in quantitative yield (1). During the latter experiment, however, a considerable increase in internal pressure was observed, which should be carefully considered when further scaling up the procedure to ensure safe operation. It is also noteworthy that both the substrate and product were highly soluble in water under the reaction conditions, which likely favoured efficient deprotection.

To assess the reproducibility of the optimized aqueous microwave-assisted deprotection protocol, four independent experiments were performed under the optimized reaction conditions. Complete conversion was observed in all runs, with an average isolated yield of 98.5 ± 0.6% (mean ± standard deviation, *n* = 4). The 1.0 g scale-up experiment was also performed in four independent runs, consistently affording an isolated yield of 99% in all cases (*n* = 4). These results support the reproducibility of the methodology under the investigated conditions and at the evaluated reaction scale.

With the optimized protocol in hand, we evaluated the substrate scope using a variety of *N*-Boc-protected substrates, including aromatic, alicyclic, and aliphatic amines bearing diverse functional groups, such as alcohols, amines, halogens, esters, carboxylic acids, and amides. The methodology consistently afforded the corresponding deprotected products in excellent yields, demonstrating broad functional-group tolerance and good versatility and robustness across structurally diverse substrates.

*N*-Boc-l-alanine (2) and *N*-Boc-l-phenylalanine (3) were first examined to extend the scope to additional α-amino acids, affording the corresponding deprotected products in quantitative yields. Because these amino acids are non-volatile, they were readily isolated by removing the water under reduced pressure, followed by drying under vacuum. To determine whether the methodology could simultaneously remove more than one Boc group, *N*,*N*′-di-Boc-l-histidine (4) was also subjected to the optimized conditions, providing histidine quantitatively and confirming efficient double deprotection.

Further insight into functional-group compatibility was obtained by subjecting *N*-Boc-l-lysine *tert*-butyl ester hydrochloride to the reaction (5). Under optimized conditions, the *tert*-butyl ester group was also cleaved, resulting in the complete deprotection of the amino acid. Finally, the scope was extended to non-α-amino acids by evaluating the deprotection of *N*-Boc-GABA (6) and *N*-Boc-11-aminoundecanoic acid (7), both of which were obtained quantitatively, demonstrating the robustness of the methodology towards diverse amino acids.

The scope of the reaction was extended to secondary alicyclic Boc-protected amines. *N*-Boc-protected 3-carboxylic acid azetidine (8), a common building block in drug discovery, was first evaluated. Azetidine-3-carboxylic acid is a medicinally relevant non-proteinogenic β-amino acid whose four-membered ring provides a useful conformational constraint in the design of bioactive molecules.^54–57^ Accordingly, its efficient preparation highlights the synthetic value of the developed deprotection methodology. Subsequently, β-proline (9), a non-α-amino acid, was also subjected to the optimized deprotection conditions. This compound has been employed as a building block in peptide and peptidomimetic design,^58–60^ bioactive compounds,^61^ and as an organocatalyst in asymmetric transformations.^62,63^ In both cases, quantitative deprotection was achieved without detectable side reactions involving either the azetidine or pyrrolidine ring.

Compounds 10 and 11 are piperidine and piperazinone derivatives, respectively. These heterocyclic scaffolds are widely used in medicinal chemistry.^64–66^ Both compounds underwent quantitative deprotection. Notably, the Nahm–Weinreb amide and lactam functionalities remained intact, indicating that the developed conditions did not promote undesired hydrolysis. Other *N*-Boc-protected compounds relevant to the synthesis of bioactive molecules were also evaluated. *N*-Boc-nortropinone (12), a tropane-based building block used in medicinal chemistry,^67,68^ was selected to assess the methodology's compatibility with a functionalized bicyclic ketone. Quantitative deprotection was achieved without detectable degradation of the bicyclic framework, further demonstrating the applicability of the developed method to structurally complex substrates.

More structurally complex piperazinone derivatives 13 and 14 were subsequently evaluated to further assess the methodology's applicability. In both cases, quantitative yields were obtained. Overall, these results demonstrate that fluoro and amino substituents are well tolerated under the developed conditions.

Given the prevalence of aniline scaffolds in bioactive molecules, robust *N*-Boc deprotection methods are important for enabling efficient synthetic access and late-stage functionalization. For aniline derivatives, the optimized conditions were 140 °C for 30 min. Higher temperatures led to the formation of by-products, as evidenced by ^1^H NMR analysis, and the isolated deprotected products developed a brownish coloration. Under the optimized conditions, the aniline-derived *N*-Boc substrates were quantitatively deprotected, as determined by ^1^H NMR analysis. The corresponding free amines were isolated in high yields after an extraction-based work-up, aniline (15, 98%), *p*-aminophenol (16, 93%), *p*-chloroaniline (17, 98%), *p*-toluidine (18, 96%), and *p*-aminobenzoic acid (19, 99%). In addition, the *N*-Boc-protected aminopyridine derivative (20) was completely deprotected within 2 min and isolated in 99% yield after solvent removal. Overall, chloro, phenolic hydroxy, and carboxylic acid functionalities were well tolerated under the optimized aqueous microwave-assisted conditions.

To further assess the functional-group tolerance and chemoselectivity of the methodology, additional ester-containing substrates were evaluated under the optimized conditions (1 mmol, 140 °C, 2 min, 2 mL H_2_O). These included l-alanine and l-phenylalanine-derived methyl, benzyl, and *tert*-butyl esters, *tert*-butyl benzoate, and (*R*)-ethyl *N*-Boc-piperidine-3-carboxylate. The results showed that ester compatibility depends on both the nature of the ester group and the structural environment of the substrate. Methyl and benzyl esters were retained under the reaction conditions, whereas ethyl and *tert*-butyl esters underwent competitive hydrolysis. These results expand the functional scope of the methodology while defining its chemoselectivity limits toward ester groups under high-temperature aqueous conditions.

The compatibility of the protocol with silyl protecting groups was also examined using the TBDPS-protected derivative of (3-(4-nitrophenyl)isoxazol-5-yl)methanol as a model substrate. Under the optimized conditions (1 mmol, 140 °C, 2 min, 2 mL H_2_O), the TBDPS group remained intact; cleavage of the TBDPS group was not observed. This experiment provided a preliminary assessment of the method's tolerance toward silyl protecting groups.

In addition, *N*-Boc-*p*-toluenesulfonamide (21) was examined to extend the scope beyond conventional Boc-protected amines. Complete deprotection was achieved within 5 min at 160 °C, affording *p*-toluenesulfonamide in quantitative yield. This result demonstrates the applicability of the methodology to *N*-Boc-protected sulfonamides, an important class of compounds in medicinal chemistry.^69^

Compounds 22 and 23 were evaluated to assess the applicability of the methodology to structurally complex substrates. Compound 22, which bears a triazole moiety, was also readily deprotected at 160 °C within 30 min. The desired product was obtained quantitatively following the removal of the water under reduced pressure. Compound 23, a precursor used in the synthesis of semaglutide, the active pharmaceutical ingredient in Ozempic.^70,71^ This compound contains a triphenylmethyl moiety, which results in very low water solubility, even at elevated temperatures. Consequently, a longer reaction time was required than for the other substrates. Interestingly, Although Boc deprotection proceeded under the optimized conditions, concomitant cleavage of the trityl protecting group was also observed, indicating limited orthogonality for this substrate. The crude product was washed with diethyl ether to remove the released triphenylmethane, affording the fully deprotected compound in 75% yield.

Additionally, *N*-Boc deprotection was evaluated using isoxazole derivative (24), which has previously been reported to exhibit inhibitory activity against coagulation factor Xa and was readily deprotected at 160 °C within 30 min.^43^

Finally, Fmoc-protected glycine (25) was subjected to the optimized reaction conditions to evaluate the chemoselectivity of the proposed methodology. Analysis by ^1^H NMR showed that the starting material remained unchanged, indicating that the methodology is selective for *N*-Boc-protected substrates while leaving the Fmoc protecting group unaffected.

The method proved broadly applicable, affording the corresponding primary and secondary amines in high yields and tolerating a wide range of functional groups, including hydroxyl, amino, halogen, ester, carboxylic acid, and amide groups. These results highlight the versatility and robustness of the methodology. Additionally, chiral amines were deprotected enantiospecifically. Given its compatibility with amide functionalities, this methodology could be readily applied to peptide synthesis or to the preparation of related chiral compounds.

To evaluate the environmental performance of the developed microwave-assisted *N*-Boc deprotection, various metrics were employed to compare the methodology with two previously reported aqueous procedures using *N*-Boc-l-alanine as a common model substrate (Table 3). Materials used during workup and isolation were consistently excluded from this comparison. These values therefore describe reaction-stage material consumption and should not be interpreted as complete-process metrics.

**Table 3:** Comparison of reaction conditions and green metrics for the aqueous deprotection of *N*-Boc-alanine

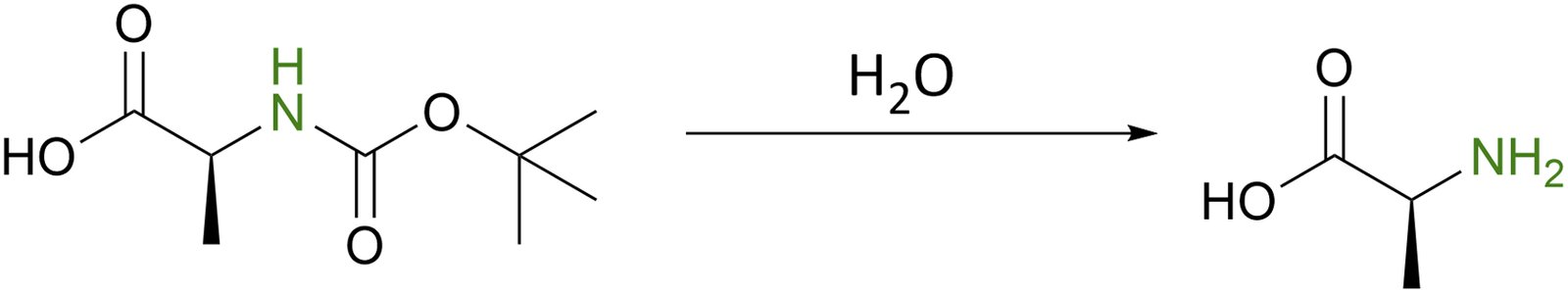				
Entry	Parameter	Procedure 1 (ref. 53)	Procedure 2 (ref. 36)	This method
1	*N*-Boc-*l*-alanine mass (mmol)	1	1	5.3
2	H_2_O volume (mL)	1	20	2
3	H_2_O use (mL mmol^−1^)	1	20	0.38
4	Temperature (°C)	140	100	150
5	Time (min)	120	240	0.5
6	Condition	Teflon®-lined stainless bomb	Reflux	Microwave
7	Yield	>99	>99	>99
8^a^	MP (%)	7.5	0.5	15.7
9^a^	MI (kg kg^−1^)	13.4	228.9	6.4
10	STY (g L^−1^ h^−1^)	44.5	1.10	2.8 × 10^4^

a Water was considered in the analysis of the parameter.

The three methods afforded l-alanine in quantitative yield but differed considerably in reaction conditions, specifically substrate concentration, reaction volume, reaction time, and heating conditions. Procedure 1 employed 1 mmol of *N*-Boc-l-alanine in 1 mL of water for 2 h at 140 °C using a Teflon®-lined stainless bomb, whereas procedure 2 used 1 mmol in 20 mL of water under reflux for 4 h. In contrast, the present methodology converted 5.7 mmol of *N*-Boc-l-alanine in 2 mL of water at 160 °C within only 30 s, quantitatively affording alanine (entries 1–6).

The efficiencies of the three procedures were assessed using three metrics: mass productivity (MP), mass intensity (MI), and space-time yield (STY).^6,72–74^ Under these conditions, metrics such as atom economy, reaction mass efficiency, and optimum efficiency do not allow discrimination between the methodologies, as they reflect the same transformation, comparable stoichiometry, and quantitative product formation. MP values (entry 8) of the present methodology and procedure 1 are substantially higher than those of procedure 2. Moreover, the present methodology shows an MI value of 6.4, the lowest among procedures 1 and 2, which show 13.4 and 228.9, respectively (entry 9). These metrics primarily reflect the improvement resulting from the lower amount of water required per unit mass of product under the microwave-assisted conditions, which required only 0.38 mL per mmol substrate.

STY was employed as a complementary indicator of process intensification (Entry 10). Procedure 1 showed an STY of 44.5 g L^−1^ h^−1^, whereas Procedure 2 showed a considerably lower value of 1.10 g L^−1^ h^−1^. In comparison, the present microwave-assisted methodology afforded a nominal reaction-stage STY of approximately 2.8 × 10^4^ g L^−1^ h^−1^, corresponding to an approximately 630-fold increase relative to Procedure 1. The higher STY value is representative of the procedure, since it accounts for the combined contributions of yield, substrate, and water loading and, most importantly, the reaction time, which can be reduced from hours to minutes or even seconds using the microwave reactor. It is important to note that STY was calculated using only the holding time at the target reaction temperature, excluding heating and cooling to maintain a consistent comparison with the literature protocols, which did not report the durations of these stages. These values, therefore, represent nominal reaction-stage productivity rather than full-cycle throughput. Isolation and purification requirements are substrate-dependent and provide an additional qualitative basis for assessing practical utility. Earlier aqueous *N*-Boc deprotection studies report procedures involving extraction followed by silica gel chromatography or recrystallization for selected substrates. In the present study, most products were isolated by solvent evaporation using ethanol to facilitate water removal and without further purification. Including the 20.0 mL of ethanol used during evaporation gives an MI of 39.9 kg kg^−1^, while MP is 2.5%. Extraction or washing was used only when required. This operational simplicity is a useful feature of the methodology, although the differences in downstream procedures cannot be translated into a quantitative comparison of total solvent consumption without complete substrate-specific input.

Microwave heating is associated with higher energy efficiency over conventional heating, particularly at the laboratory scale.^75,76^ The reaction temperature could be reduced to industrially accepted temperatures^74^ while maintaining quantitative conversion in a few minutes (Fig. 4). These results demonstrate considerable process intensification across the entire temperature range investigated. From a green chemistry perspective, the availability of several quantitative conditions is particularly relevant, as the condition that yields the shortest reaction time may not necessarily correspond to the lowest overall energy demand. However, identifying the most energy-efficient condition would require measuring the electricity consumed over the complete heating, holding, and cooling cycle, ideally using an external wattmeter. Therefore, no definitive energy-efficiency comparison with conventional heating is claimed. This approach is consistent with previous critical assessments indicating that the energy efficiency of microwave-assisted organic synthesis should be evaluated on a case-by-case basis rather than assumed from microwave irradiation alone.^77^

**Fig. 4:**
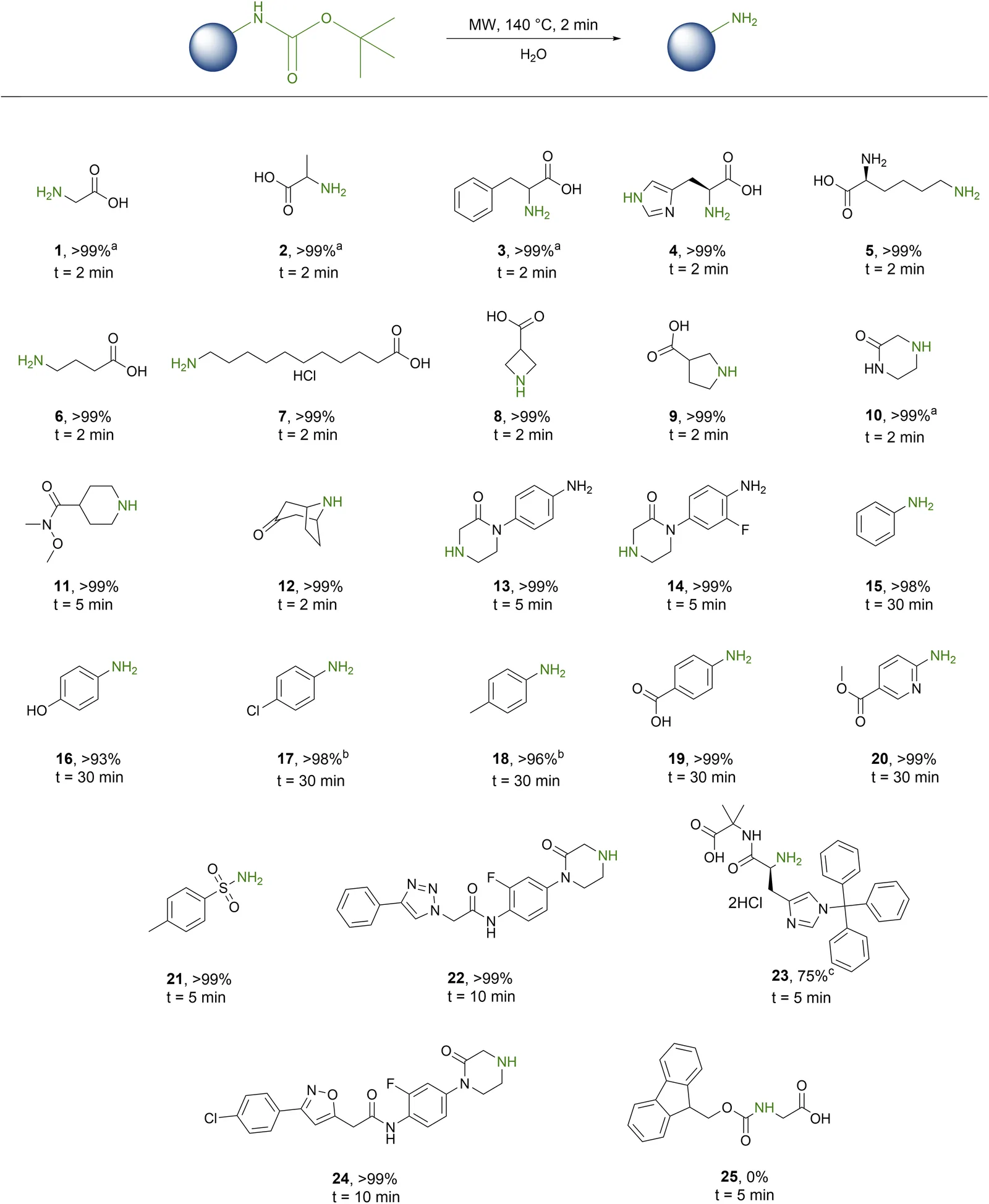
Substrate scope and functional-group tolerance of the microwave-assisted aqueous *N*-Boc deprotection protocol. ^*a*^Reaction performed on a 1.0 g scale. ^*b*^Products isolated by Et_2_O extraction; conversion determined by ^1^H NMR. ^*c*^An additional purification step involving treatment with diethyl ether followed by filtration was performed for the product obtained from compound 23. See SI for details.

For the optimized conditions selected in this study (1 mmol, 140 °C, 30 s), an instrument-based estimate of the apparent microwave energy input was calculated as *E* = *P* × *t*, where *P* is the power displayed by the Anton Paar Monowave 400.^78^ Although the reactor has a maximum microwave output power of 850 W, the displayed power during irradiation was typically 15–20 W. Thus, the apparent microwave energy input was estimated as 0.015–0.020 kW × 0.00833 *h* = 0.000125–0.000167 kWh, corresponding to 0.125–0.167 kWh mol^−1^ for a 1 mmol reaction. These values represent apparent microwave energy input rather than total electrical consumption, since ramp-up/cooling periods, magnetron efficiency, and auxiliary energy demands were not experimentally determined. Accordingly, the sustainability benefits of the protocol are mainly associated with the use of water, avoidance of corrosive acids and catalysts, reduced solvent demand, and short reaction time (Fig. 5).

**Fig. 5:**
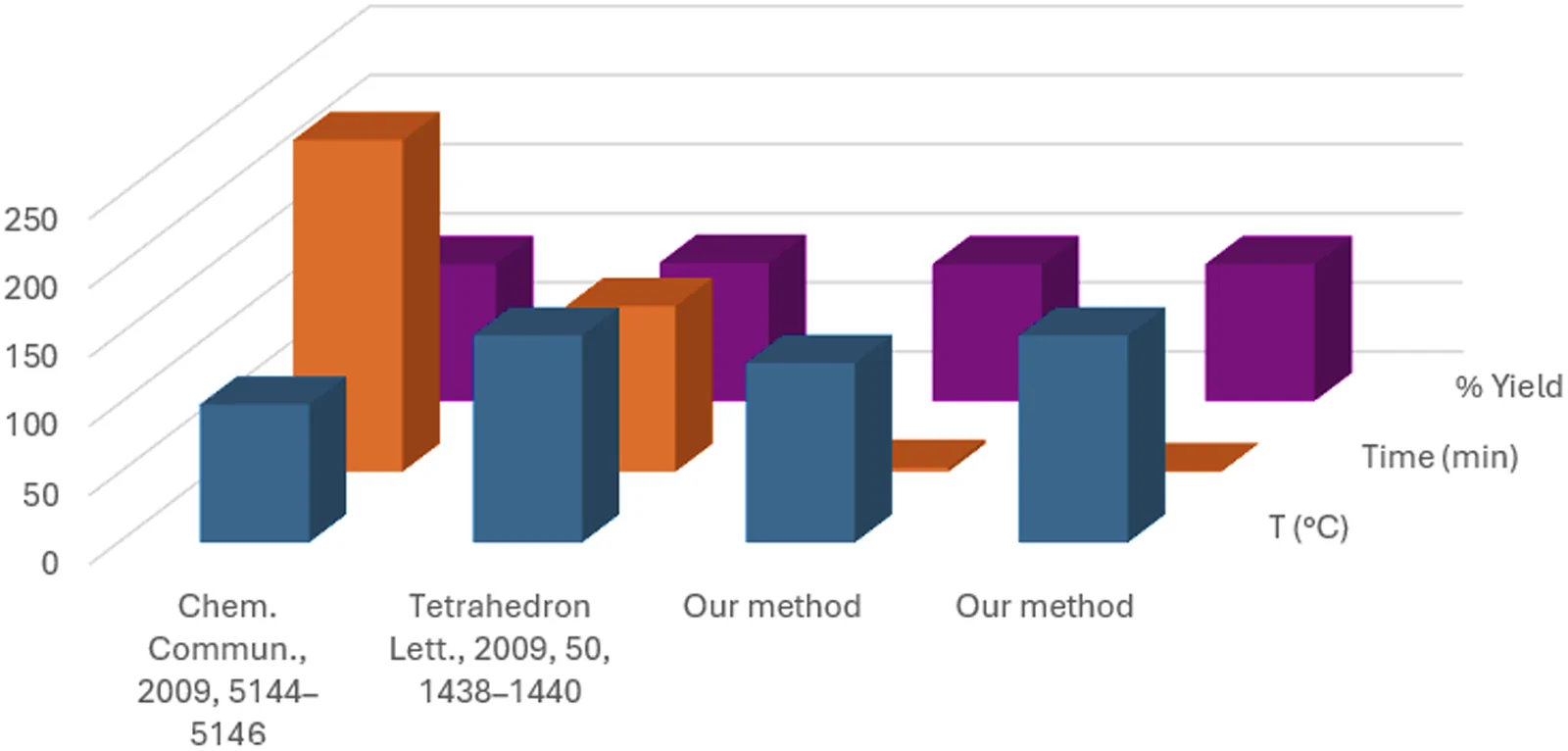
Comparison of reaction conditions reported in the literature and the present protocol. The bars summarize reaction temperature (°C), reaction time (min), and isolated yield (%) for representative entries from the literature (*Chem. Commun.*, 2009, 5144–5146; *Tetrahedron Lett.*, 2009, **50**, 1438–1440) and this work; numerical values are listed in the table below.

Overall, the temperature screening demonstrates that the methodology offers operational flexibility while maintaining quantitative conversion. The 160 °C condition maximizes temporal productivity, whereas the lower-temperature conditions reduce the maximum operating temperature while still providing reaction times substantially shorter than those of the reported aqueous methods. Therefore, the selection of the preferred condition may depend on whether priority is given to maximum throughput, lower operating temperature, equipment limitations, or complete-cycle energy consumption.

Finally, the successful scale-up of the reaction to a 1.0 g substrate loading, while maintaining quantitative conversion, provides encouraging evidence of the robustness and practical potential of the microwave-assisted protocol. However, microwave-assisted reaction scale-up has been associated with some limitations.^79^ Therefore, further scale-up should consider factors such as microwave penetration depth, field and temperature uniformity, mixing efficiency, and pressure control, since CO_2_ release during the reaction considerably increases internal pressure as the substrate mass increases. Thus, the present result demonstrates promising preliminary scalability and supports the methodology's potential application at larger scales following appropriate reactor and process optimization.

Taken together, clear differences emerged in water consumption and reaction time, as reflected in MI, MP, and STY. The present methodology required only 0.38 mL of water per mmol of substrate, resulting in the lowest MI value and the highest mass productivity among the compared procedures. In addition, microwave-assisted heating enabled quantitative deprotection within 30 s at 160 °C, while quantitative conversion was also maintained within only a few minutes at lower temperatures. These markedly shorter processing times led to a substantially higher nominal STY than conventional thermal methods and are consistent with a potentially lower overall energy demand. Together with the successful scale-up to a 1 g substrate loading, these results support the developed protocol as a rapid, resource-efficient, and promisingly scalable alternative for aqueous *N*-Boc deprotection.

### 3.2 Molecular modelling

Molecular modelling was based on the mechanistic proposal described by Li *et al.*,^50^ focusing on the characterization of the transition state involved in the *N*-Boc deprotection pathway (Fig. 6). A plausible water-assisted thermolytic pathway involving proton transfer and *tert*-butyl cleavage to generate carbamic acid and isobutene, followed by rapid decarboxylation. Under the aqueous reaction conditions, isobutene may subsequently be hydrated to *tert*-butanol.

**Fig. 6:**
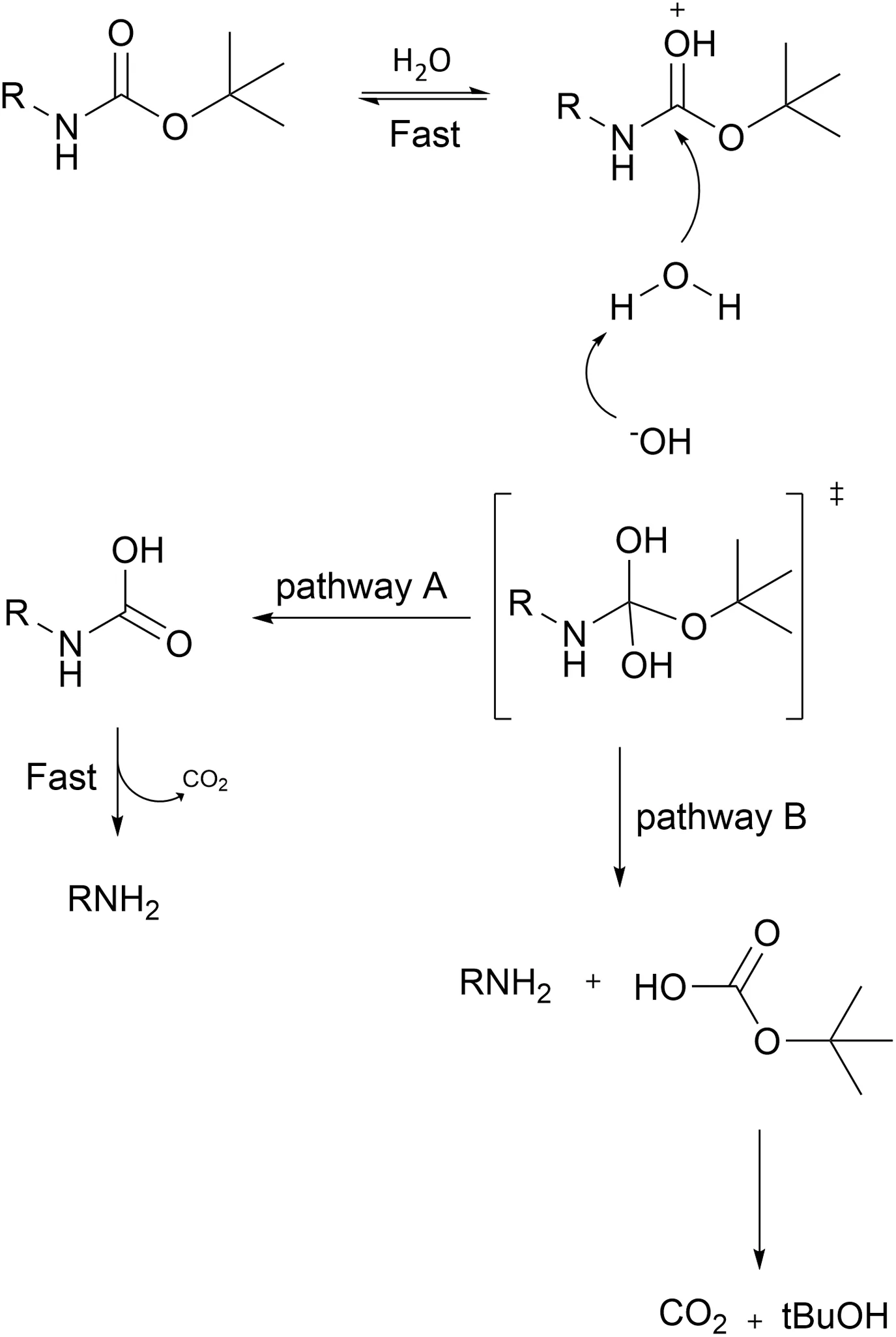
Proposed reaction mechanism for water-catalyzed *N*-Boc deprotection, as reported by Wang *et al.*^36^

To analyze differences in chemical reactivity among the substrates and products involved in the aqueous *N*-Boc deprotection process, global reactivity descriptors were calculated. The descriptors correspond to the energy gap (Δ*E*), global hardness (*η*), softness (*S*), chemical potential (*µ*), and the global electrophilicity index (*ω*), as defined within the framework of conceptual DFT^80–82^ (see SI for details).

According to Parr and Pearson, using a finite-difference approximation, the *µ* and *η* parameters are estimated from the ionization potential (*I*) and electron affinity (*A*); moreover, applying Koopmans' theorem, they can be expressed as functions of the HOMO and LUMO orbital energies. In physical terms, *µ* relates to the tendency of electrons to escape and to charge rearrangement in a chemical process, while *η* represents resistance to charge transfer.

1 *µ* = ½(*ε*_L_ + *ε*_H_)

2 *η* = ½(*ε*_L_ − *ε*_H_)

In addition, molecular softness (*S*) and the electrophilicity index (*ω*) describe the electronic redistribution and electrophilic capacity of the system, respectively. Formally, softness is defined as the inverse of hardness and allows the study of charge transfer in response to a perturbation. In turn, electrophilicity simultaneously integrates the tendency to accept electron density (*µ*) and the resistance to this process (*η*).3
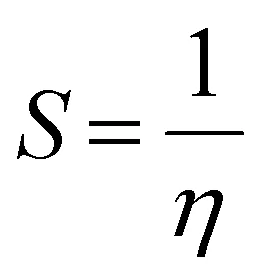
4
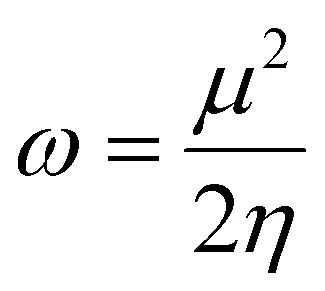


As previously described, under microwave-assisted hydrothermal conditions, the reaction medium corresponds to subcritical water, which allows operation in the liquid phase at high temperature (*P*_sat_ ≈ 0.618 MPa at 160 °C). Under these conditions, the physicochemical properties of water change markedly; the dielectric constant decreases (*ε* ≈ 41.6 at 160 °C), thereby increasing the solvent's compatibility with organic substrates and favoring its solubilization and adequate mixing (Fig. 7).^83^

**Fig. 7:**
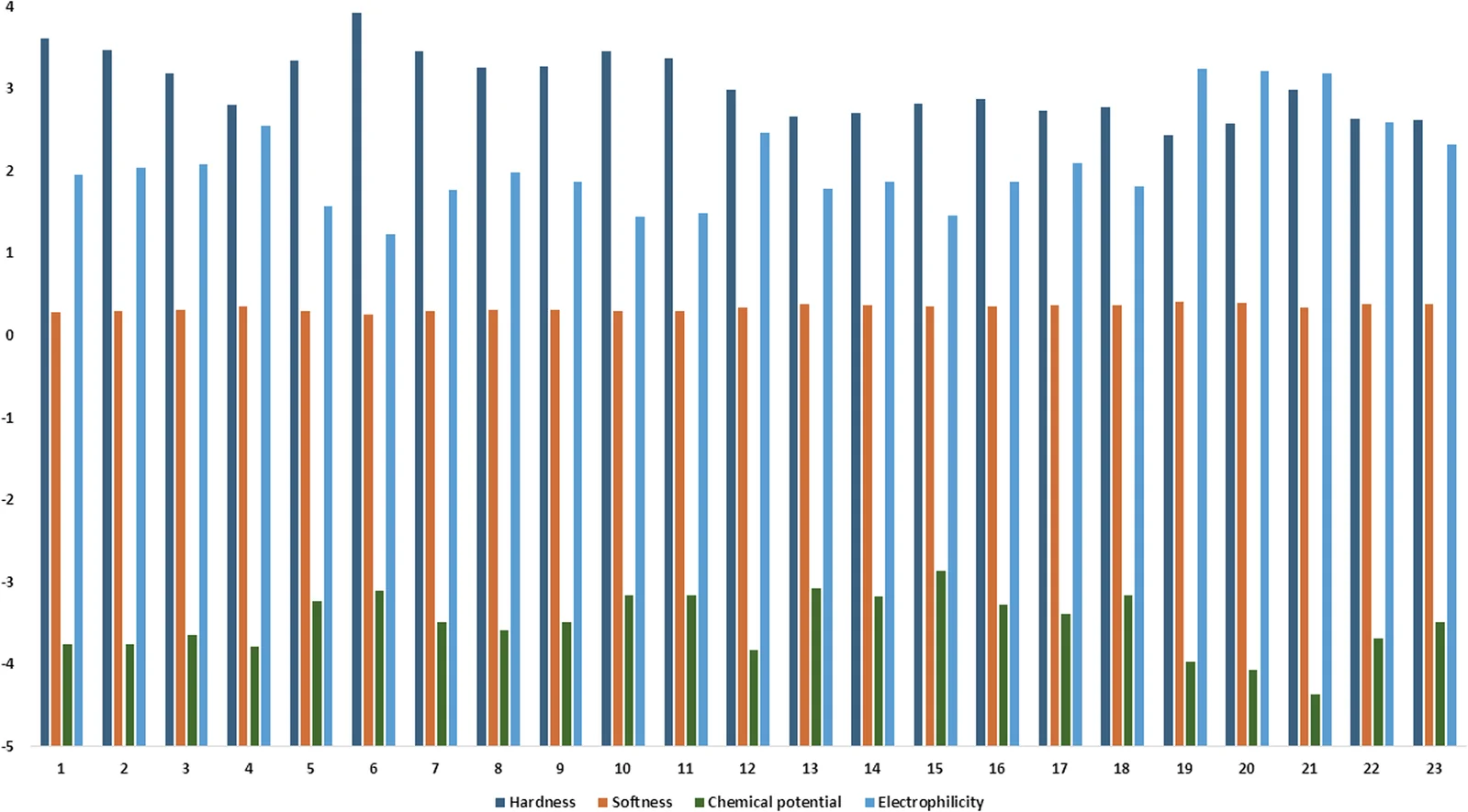
Frontier molecular orbital (FMO) energies and global reactivity indices of the *N*-Boc reagent(s) used in this work.

Additionally, the ionic product of water increases with temperature (p*K*_w_ ≈ 11.56 at 160 °C),^84^ increasing the intrinsic availability of acid/base species that can mediate relevant protonation and solvolysis stages in carbamate cleavage. Our work considers microwave irradiation, which enables rapid attainment of high temperatures and precise control of reaction times to achieve optimal *N*-Boc deprotection.^85^

In general, the deprotected products exhibit slightly lower HOMO and LUMO energies compared to their corresponding substrates, indicating increased electronic stabilization after the deprotection reaction. In most cases, an increase in the HOMO–LUMO energy gap (Δ*E*) is also observed, suggesting a decrease in global reactivity and a greater thermodynamic stability of the products. This trend is consistent with the Maximum Hardness Principle, which postulates that molecular systems tend to evolve toward electronically harder states, *i.e.*, those with larger gaps and higher resistance to external perturbations.^86,87^

Moreover, the chemical potential (*µ*) remains negative for all species, reflecting an intrinsic stability toward electron uptake. A slight increase in electronegativity (*χ*) is also observed in the products relative to their precursors. This may be attributed to a more polarized electron density distribution after cleavage of the carbamate moiety, potentially enhancing hydrogen-bonding interactions with the aqueous medium, improving solvation.^88,89^

As shown in Fig. 8, the analysis of the overall electrophilicity index (*ω*) revealed that the studied substrates exhibited comparatively higher values before and after deprotection. These compounds may tend to accept electron density, which would facilitate electronic stabilization during *N*-Boc bond cleavage. Conversely, the overall decrease in softness (*S*) observed in most deprotected products suggests a lower capacity for electron density reorganization, consistent with a more electronically rigid post-deprotection state.

**Fig. 8:**
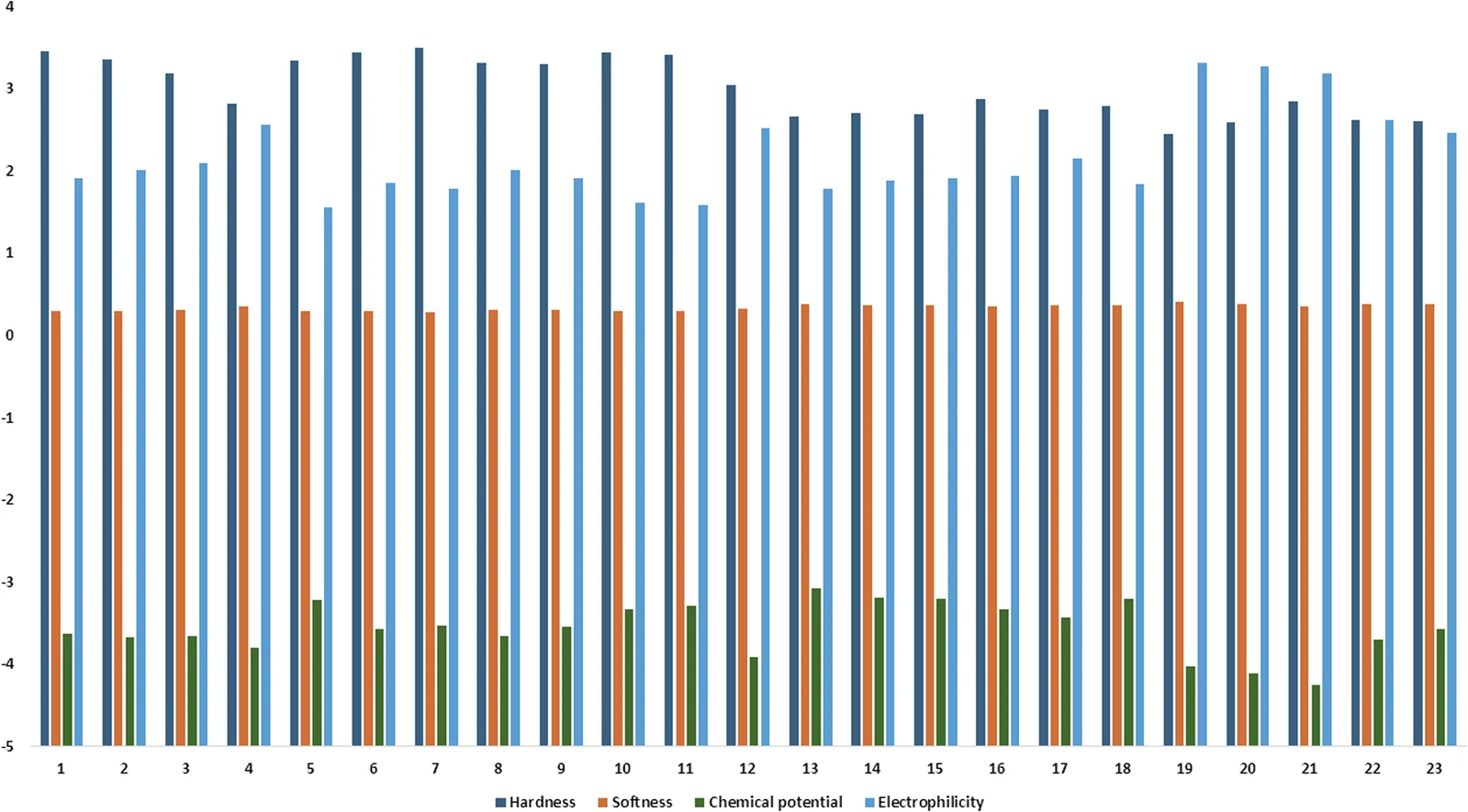
Frontier molecular orbital (FMO) energies and global reactivity indices of the deprotected products obtained in this study.

According to the calculated parameters, it is possible to determine that the overall reactivity of each substrate, compared to the experimental differences observed between substrates under water conditions, probably depends on local factors, such as the polarization of the carbamate motif, solubility, and conformational accessibility, as well as solvent-temperature coupling in subcritical water. When contrasting the amino acids *N*-Boc (1–4) with aromatic substrates derived from protected anilines (15–18), it is observed that the global descriptors derived from HOMO/LUMO do not markedly separate both groups. On the one hand, anilines exhibit *ω* in the range 1.46–2.10 eV and *µ* in the range −2.82 to −3.39 eV. At the same time, Boc-glycine (1) shows *ω* ≈ 1.87 and *µ* ≈ −3, 18 eV, and Boc-alanine (2) *ω* ≈ 2.59 and *µ* ≈ −3.70 eV.

This behavior indicates that reaction times depend not only on the overall substrate reactivity, but also on local effects and the surrounding reaction medium; accordingly, the rapid conversion of 3 and 8 aligns with reports that microwave-assisted *N*-Boc deprotection in water is especially efficient for substrates containing a free carboxylic acid group, likely because it facilitates proton-transfer steps in the absence of an external acid catalyst.^90^

In addition, protected amino acid substrates have a more polar and ionizable character than aniline derivatives, which favors both their dielectric coupling to the microwave and their interaction with the reaction medium; in contrast, *N*-Boc anilines, which are less polar and without an intrinsic acid function, rely more heavily on substrate activation by the reaction medium to access sufficiently polarized states, leading to longer reaction times.

Altogether, these results provide a quantitative electronic characterization of the molecular species involved and establish a meaningful correlation between reactivity descriptors and experimental behavior. This theoretical insight complements experimental observations and reinforces the use of water as a sustainable medium for deprotection processes in alignment with the Principles of Green Chemistry.

Representative substrates were selected from the main structural families examined experimentally during *N*-Boc deprotection (Table 4). These included carboxylic acid-containing compounds, represented by Boc-phenylalanine (3); constrained nitrogen heterocycles, represented by the azetidine (8) and oxopiperazinone (10) derivatives; and aromatic carbamates, represented by aniline (15), *p*-hydroxyaniline (16), and *p*-chloroaniline (17). The computational analysis was intended to compare relative energetic trends within a simplified Boc-cleavage model and not to provide a quantitative kinetic model of the experimental reaction at 120–160 °C. The reported Δ*G*, Δ*H*, and TΔS values correspond to standard thermochemical corrections evaluated at 298.15 K.

**Table 4:** Comparison of calculated activation parameters for the simplified *N*-Boc deprotection model using H_2_O as implicit solvent. Thermochemical corrections were evaluated at 298.15 K

Entry	*E* ^‡^_a_ (kcal mol^−1^)	Δ*G*^‡^ (kcal mol^−1^)	Δ*H*^‡^ (kcal mol^−1^)	*T*Δ*S*^‡^ (kcal mol^−1^)
3	32.34	27.40	28.29	0.88
8	33.64	28.36	29.49	1.13
10	35.22	30.33	31.26	0.93
15	33.70	29.18	29.01	−0.17
16	33.18	28.18	29.14	0.96
17	32.82	27.90	28.83	0.93

Within this model, the calculated activation free energies in implicit water ranged from 27.40 to 30.33 kcal mol^−1^, indicating moderate substrate-dependent variations in the modeled Boc-cleavage step. These differences should therefore be interpreted as relatively energetic descriptors within a consistent computational framework, rather than as direct predictors of the reaction times observed under the experimental high-temperature microwave-assisted conditions. Within the aromatic series, the unsubstituted aniline derivative (15) showed a higher calculated barrier than the *p*-hydroxy- (16) and *p*-chloro-substituted (17) analogues. The decrease from 29.18 kcal mol^−1^ for 15 to 28.18 and 27.90 kcal mol^−1^ for 16 and 17, respectively, suggests that *para* substitution can moderately affect the modeled cleavage step. However, because the OH group and Cl atom exert different electronic effects, this trend cannot be rationalized solely in terms of a simple electron-donating or electron-withdrawing argument. Instead, it likely reflects the combined influence of ring polarization, aqueous solvation, and the local environment of the carbamate group. The small *T*Δ*S*^‡^ values obtained at 298.15 K suggest that, within this model, the differences in Δ*G*^‡^ mainly arise from enthalpic contributions (Fig. 9).

**Fig. 9:**
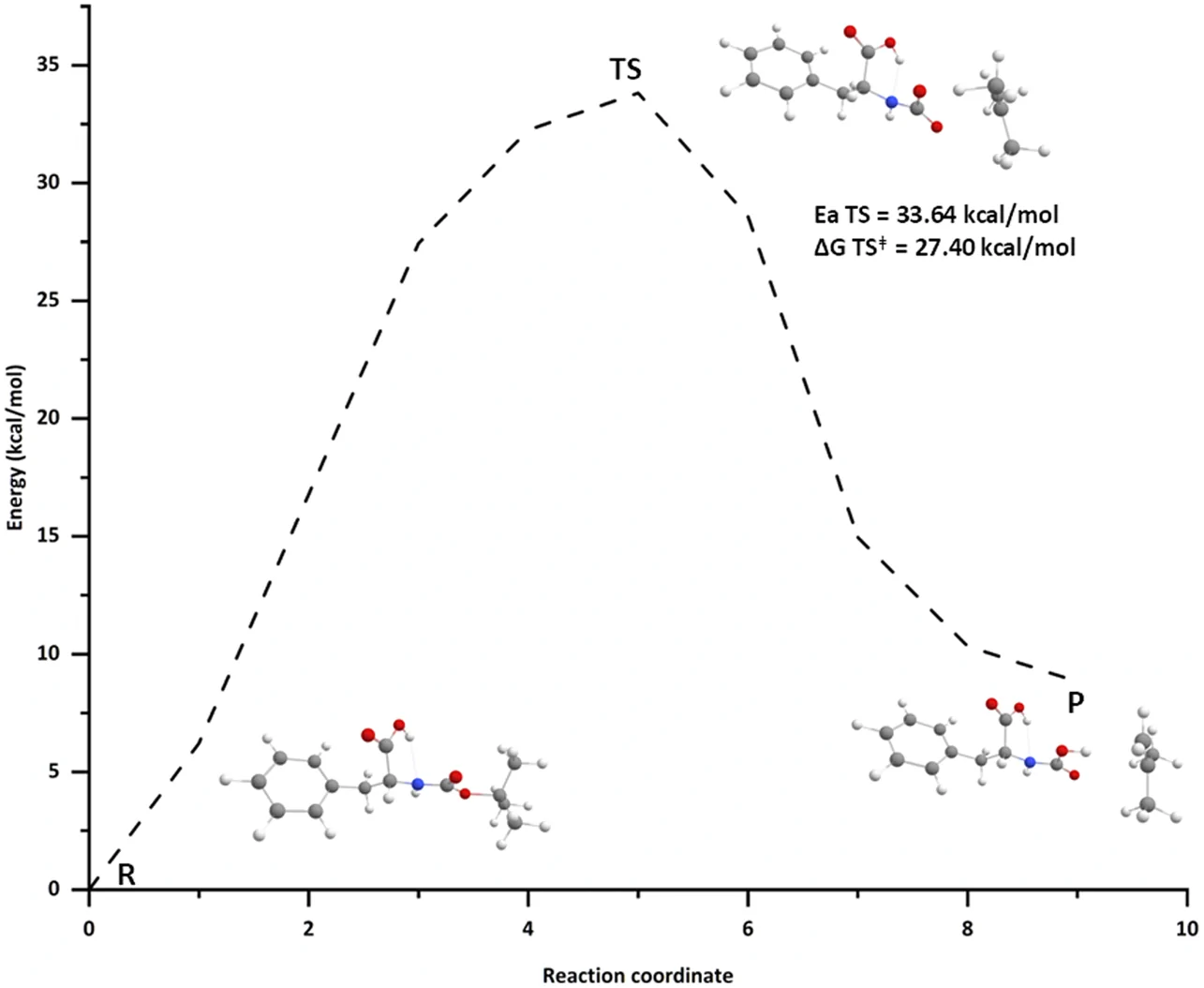
Representative minimum-energy profile for *N*-Boc deprotection of (3) computed at B3LYP/def2-TZVP-D4 with CPCM(water).

The simplified reaction free energies were positive for all representative substrates (Table 5), ranging from 4.19 to 6.95 kcal mol^−1^. The azetidine (8) and oxopiperazinone (10) derivatives showed the lowest Δ*G*_Rxn_ values, whereas the *p*-hydroxy- and *p*-chloroaniline derivatives 16 and 17 exhibited the highest values. However, these Δ*G*_Rxn_ values should be interpreted only as internal relative comparisons within the reduced R → P model. The absolute values should not be taken as direct estimates of the experimental thermodynamic driving force, because acid–base equilibria, proton-transfer events, solvent participation, and the formation of Boc-derived coproducts were not explicitly included.

**Table 5:** Energy comparison for the simplified global R → P reaction model of *N*-Boc deprotection. Thermochemical corrections were evaluated at 298.15 K

Entry	Δ*E* (kcal mol^−1^)	Δ*G*_Rxn_ (kcal mol^−1^)	Δ*H*_Rxn_ (kcal mol^−1^)	*T*Δ*S* (kcal mol^−1^)
3	8.81	5.43	8.02	2.59
8	7.61	4.19	6.14	1.95
10	7.58	4.28	6.86	2.58
15	8.80	4.50	8.63	4.13
16	9.21	6.84	7.91	1.07
17	9.31	6.95	8.00	1.05

It is worth noting that compound 10 exhibited the highest calculated activation barrier and one of the lowest reaction free energies, indicating that the kinetic and thermodynamic descriptors obtained from the simplified model do not follow the same structural trend. This observation further supports the use of the calculated values as qualitative mechanistic descriptors rather than as quantitative predictors of experimental reactivity.

Overall, the calculated activation barriers support a thermally activated *N*-Boc cleavage process whose relative energetic requirements depend on the substrate's electronic, conformational, and solvation environments. Because the thermochemical corrections were evaluated at 298.15 K and microwave irradiation was not explicitly included in the computational model, these calculations should not be used to quantitatively rationalize the experimentally observed temperature-dependent reaction times. Instead, they provide molecular-level support for the role of aqueous solvation in stabilizing polarized structures along the modeled Boc-cleavage pathway.

## 4. Conclusions

In conclusion, we have developed a rapid and efficient methodology for the *N*-Boc deprotection of structurally diverse amines in aqueous media under microwave-assisted conditions. The protocol provides quantitative conversions and high isolated yields across a broad substrate scope, including amino acid derivatives, aniline derivatives, heterocyclic amines, sulfonamide-containing substrates, and complex drug-relevant scaffolds. Additional functional-group studies showed useful, although substrate-dependent, compatibility with ester-containing substrates, with methyl and benzyl esters being retained under the optimized conditions, whereas more labile ethyl and *tert*-butyl esters underwent competitive hydrolysis. These results define both the synthetic utility and the chemoselectivity limits of the aqueous high-temperature protocol. From a sustainability perspective, the methodology reduces the use of corrosive acids, organic solvents, and catalysts in the deprotection step, while enabling short reaction times in water. Although direct electrical energy consumption was not measured and no definitive energy-efficiency comparison with conventional heating was claimed, a nominal energy-input estimate was included to provide a more transparent evaluation of the microwave-assisted process. DFT calculations provided molecular-level insight into the reaction mechanism, highlighting the stabilizing role of water on polarized transition states and supporting the experimentally observed acceleration under aqueous microwave-assisted conditions.

Overall, the combination of water as the reaction medium, minimal auxiliary inputs, operational simplicity, and rapid microwave heating positions this methodology as a practical and more sustainable alternative for *N*-Boc deprotection in medicinal chemistry and related synthetic applications. While the present study was developed and validated under batch microwave conditions, the short reaction times observed here suggest that future translation to a pressurized continuous-flow platform is feasible, in line with previous reports on high-temperature continuous-flow *N*-Boc deprotection.

## Author contributions

Conceptualization, J. V. N., S. M. G. and F. C. Z.; methodology, J. V. N., S. M. G. and F. C. Z.; software, J. V. N. and E. S.; validation, J. V. N, S. M. G., and F. C. Z. and F. C. Z.; formal analysis, J. V. N., S. M. G.; investigation, J. V. N, and S. M. G.; resources, E. S and F. C. Z.; data curation, J. V. N., and S. M. G.; writing—original draft preparation, J. V. N., and S. M. G. and F. C. Z.; writing—review and editing, J. V. N., S. M. G., E. S., L. R., and F. C. Z.; visualization, J. V. N., S. M. G., E. S., and F. C. Z.; supervision, F. C. Z.; project administration, F. C. Z.; funding acquisition, F. C. Z. All authors have read and agreed to the published version of the manuscript.

## Conflicts of interest

The authors declare no conflict of interest.

## Supplementary Material

RA-016-D6RA08533D-s001

## Data Availability

The data supporting this article have been included as part of the supplementary information (SI). Supplementary information is available. See DOI: https://doi.org/10.1039/d6ra08533d.
