# Supplementary material for: Greener deprotection pathways: microwave assisted aqueous deprotection of N-Boc amines
Source: RSC Adv. 2026 Oct 26. Online ahead of print. doi: 10.1039/d6ra08533d (PMC13701105; doi:10.1039/d6ra08533d)
Supplement: Supplementary file 1 [file RA-OLF-D6RA08533D-s001.pdf]

Supplementary Information (SI) for RSC Advances.
This journal is © The Royal Society of Chemistry

Supporting Information

Greener deprotection pathways:

Microwave assisted aqueous deprotection of *N*-Boc amines

José Vidal-Núñez[a]; Sebastián Morales-Guerrero[a]; Lars Ratjen[b]; Eduardo Schott[c]; Flavia C. Zacconi[a,d,*]

[a] Departamento de Química Orgánica, Facultad de Química y de Farmacia, Pontificia Universidad Católica de Chile, Vicuña Mackenna 4860, Santiago 7820436, Chile.

[b] Center for Green Chemistry and Green Engineering, School of the Environment, Yale University, New Haven, Connecticut 06511, United States.

[c] Departamento de Química Inorgánica, Facultad de Química y de Farmacia, CE-UC, CIEN-UC, Pontificia Universidad Católica de Chile, Avenida Vicuña Mackenna, 4860, Santiago, Chile.

[d] Institute for Biological and Medical Engineering, Schools of Engineering, Medicine and Biological Sciences, Pontificia Universidad Católica de Chile, Av. Vicuña Mackenna 4860, Macul, Santiago 7820436, Chile

Contents

General Procedure for *N*-Boc Deprotection 4
General procedure for 13 and 14 derivatives[1] 5
General procedure of the peptide coupling reaction for 22 and 24 derivatives[1,2] 5
Characterization Data 6
Computational details 32

Index of figures

Figure 1. ^{1}H-NMR spectrum of *L*-alanine (**2**) in D_2O 9
Figure 2. ^{1}H-NMR spectrum of *L*-phenylalanine (**3**) in D_2O 9
Figure 3. ^{1}H-NMR spectrum of *L*-histidine (**4**) in D_2O 10
Figure 4. ^{1}H-NMR spectrum of *L*-lysine (**5**) in D_2O 10
Figure 5. ^{1}H-NMR spectrum of GABA (**6**) in D_2O 11
Figure 6. ^{1}H-NMR spectrum of 11-aminoundecanoic acid (**7**) hydrochloride salt in D_2O 11
Figure 7. ^{13}C-NMR spectrum of 11-aminoundecanoic acid (**7**) hydrochloride salt in D_2O 12
Figure 8. ^{1}H-NMR spectrum of azetidine-3-carboxylic acid (**8**) in D_2O 12
Figure 9. ^{1}H-NMR spectrum of pyrrolidine-3-carboxylic acid (**9**) in D_2O 13
Figure 10. ^{1}H-NMR spectrum of piperazin-2-one (**10**) in DMSO-d_6 13
Figure 11. ^{1}H-NMR spectrum of *N*-methoxy-*N*-methylpiperidine-4-carboxamide (**11**) in DMSO-d_6 14
Figure 12. ^{1}H-NMR spectrum of (1*R*,5*S*)-8-azabicyclo[3.2.1]octan-3-one (**12**) in $CDCl_3$ 14
Figure 13. ^{1}H-NMR spectrum of 1-(4-aminophenyl)piperazin-2-one (**13**) 15
Figure 14. 1H-NMR spectrum of 1-(4-amino-3-fluorophenyl)piperazin-2-one (**14**) 15
Figure 15. ^{1}H-NMR spectrum of aniline (**15**) in DMSO-d_6 16
Figure 16. Stacked ^{1}H-NMR spectrum of *N*-Boc aniline (top) and aniline (**15**) (bottom) 17
Figure 17. Superimposed ^{1}H-NMR spectrum of *N*-Boc aniline (Red) and aniline (**15**) (blue) .. 17
Figure 18. ^{1}H-NMR spectrum of 4-aminophenol (**16**) in DMSO-d_6 18
Figure 19. ^{1}H-NMR spectrum of 4-chloroaniline (**17**) in $CDCl_3$ 18
Figure 20. ^{1}H-NMR spectrum of 4-methylaniline (**18**) in DMSO-d_6 19
Figure 21. ^{1}H-NMR spectrum of 4-aminobenzoic acid (**19**) in DMSO-d_6 20
Figure 22. ^{1}H-NMR spectrum of Methyl 6-aminonicotinate (**20**) in DMSO-d_6 20
Figure 23. ^{1}H-NMR spectrum of 4-methylbenzenesulfonamide (**21**) in DMSO-d_6 21
Figure 24. ^{1}H-NMR spectrum of *N*-(2-fluoro-4-(2-oxopiperazin-1-yl)phenyl)-2-(4-phenyl-1*H*-1,2,3-triazol-1-yl)acetamide (**22**) in DMSO-d_6. 22
Figure 25. ^{13}C-NMR spectrum of *N*-(2-fluoro-4-(2-oxopiperazin-1-yl)phenyl)-2-(4-phenyl-1*H*-1,2,3-triazol-1-yl)acetamide (**22**) in DMSO-d_6. 23

Figure 26. ^{19}F-NMR spectrum of *N*-(2-fluoro-4-(2-oxopiperazin-1-yl)phenyl)-2-(4-phenyl-1*H*-1,2,3-triazol-1-yl)acetamide (**22**) in DMSO-d_6. 24
Figure 27. HRMS ESI Spectrum compound **22**.......... 24
Figure 28. ^{1}H-NMR spectrum of 2-(2-Amino-3-(1*H*-imidazol-4-yl)propanamido)-2-methylpropanoic acid (**23**) in DMSO-d_6. 25
Figure 29. ^{1}H-NMR spectrum of 2-(3-(4-chlorophenyl)isoxazol-5-yl)-*N*-(2-fluoro-4-(2-oxopiperazin-1-yl)phenyl)acetamide (**24**) in DMSO-d_6. 26
Figure 30. ^{13}C-NMR spectrum of 2-(3-(4-chlorophenyl)isoxazol-5-yl)-N-(2-fluoro-4-(2-oxopiperazin-1-yl)phenyl)acetamide (24) in DMSO-d_6. 26
Figure 31. ^{1}H-NMR spectrum of *N*-((9-fluorenyl)methoxycarbonyl)glycine (**25**) in DMSO-d_6.. 27
Figure 32. ^{1}H-NMR spectrum of [3-(4-nitrophenyl)isoxazol-5-yl]methanol. 28
Figure 33. ^{1}H NMR spectrum of 5-(((*tert*-butyldiphenylsilyl)oxy)methyl)-3-(4-nitrophenyl)isoxazole 29
Figure 34. ^{1}H NMR spectrum (400 MHz, $CDCl_3$) of 5-(((*tert*-butyldiphenylsilyl)oxy)methyl)-3-(4-nitrophenyl)isoxazole after treatment with water under microwave irradiation (140 °C, 2 min).... 30
Figure 35. Stacked ^{1}H NMR spectra (400 MHz, $CDCl_3$) of **(a)** [3-(4-nitrophenyl)isoxazol-5-yl]methanol (I), **(b)** the corresponding TBDPS-protected derivative (II), and **(c)** compound II after treatment under the optimized microwave-assisted *N*-Boc deprotection conditions (H_2O, 140 °C, 2 min). The isoxazole CH, CH_2, and *tert*-butyl resonances appear as two sets of signals in an approximate 65:35 ratio, suggesting hindered rotation of the silyl ether and the presence of rotamers.[3] 31
Figure 36. Input example optimization reagent and product 33
Figure 37. Input file NEB TS 33

Index of tables

Table 1. Global reactivity descriptors, and HOMO LUMO orbital energies of the reactants. Global reactivity descriptors, and HOMO LUMO orbital energies of the reactants. 26
Table 2. HOMO/LUMO orbital representation of the calculated reagents 26
Table 3. Global reactivity descriptors, and HOMO LUMO orbital energies of the products. 31
Table 4. HOMO/LUMO orbital representation of the calculated products 32
Table 5. Global termochemistry (P-R) 37
Table 6. Path summary for the NEB-TS calculation obtained from ORCA (**3**) 38
Table 7. Path summary for the NEB-TS calculation obtained from ORCA (**8**) 38
Table 8. Path summary for the NEB-TS calculation obtained from ORCA (**9**) 38
Table 9. Path summary for the NEB-TS calculation obtained from ORCA (**10**) 39
Table 10. Path summary for the NEB-TS calculation obtained from ORCA (**11**) 39
Table 11. Path summary for the NEB-TS calculation obtained from ORCA (**13**) 40
Table 12. Path summary for the NEB-TS calculation obtained from ORCA (**14**) 40
Table 13. Path summary for the NEB-TS calculation obtained from ORCA (**15**) 40
Table 14. Path summary for the NEB-TS calculation obtained from ORCA (**16**) 41
Table 15. Path summary for the NEB-TS calculation obtained from ORCA (**17**) 41

Table 16. Path summary for the NEB-TS calculation obtained from ORCA (**18**) 41
Table 17. Path summary for the NEB-TS calculation obtained from ORCA (**20**) 42
Table 18. Path summary for the NEB-TS calculation obtained from ORCA (**22**) 42

General Procedure for *N*-Boc Deprotection

For the deprotection of *N*-Boc derivatives, 1.0 mmol of substrate was placed in a G10 microwave reaction tube with 2.0 mL of HPLC-grade water. The tube was sealed and subjected to microwave irradiation at an initial power of 850 watts (W) and a target temperature of 140 °C for 2 minutes.

Upon completion, the reaction mixture was cooled to room temperature, diluted with 20.0 mL of ethanol, and subsequently evaporated under reduced pressure (70 mbar, 40 °C) to yield the corresponding free amine. The isolated products were obtained quantitatively without the need for further purification.

General procedure for 13 and 14 derivatives[1]

A mixture of CuI (0.5 mmol, 95.0 mg), 3-fluoro-4-iodoaniline (3.0 mmol, 711 mg), *δ*-valerolactame (2.5 mmol, 248 mg), K_3PO_4 (5.0 mmol, 1061 mg), *N,N'*-dimethylethylenediamine (DMEDA) (1.0 mmol, 108 µL) and anhydrous 2-MeTHF (6 mL) was placed in the microwave reactor (Synthesis Reactor Monowave 400, Anton Paar) and irradiated continuously with initial power (850 W) at 120 °C for 2 h. The crude residue was purified by flash column chromatography on hand-packed columns, with silica gel and hexane/ethyl acetate as the solvent mixture, to obtain the corresponding isolated arylamines.

General procedure of the peptide coupling reaction for 22 and 24 derivatives[1,2]

A mixture of hexafluorophosphate azabenzotriazole tetramethyl uronium (HATU) (0.5 mmol, 190.1 mg), 1-ethyl-3-(3-dimethylaminopropyl)carbodiimide hydrochloride (EDC) (0.5 mmol, 95.9 mg), and 2-(4-phenyl-1*H*-1,2,3-triazol-1-yl)acetic acid (0.5 mmol, 101.6 mg) was weighed and transferred into a dried reaction tube. The tube was evacuated and backfilled with dry N_2. The reaction was stirred at 0 °C for 1 h in dry dichloromethane (DCM) (8 mL). Subsequently, the 1-(4-amino-2-fluorophenyl)piperidin-2-one (0.65 mmol, 135.4 mg) and pyridine (Py) (0.5 mmol, 40 µL) were added, and the reaction was placed in the microwave reactor (Synthesis Reactor Monowave 400, Anton Paar) and irradiated with initial power (850 W) at 70 °C for 30 min. The remaining mixture was washed once with an aqueous solution of citric acid (10% w/w), saturated aqueous $NaHCO_3$, brine and dried over anhydrous $MgSO_4$. The solvent was removed under vacuum, and the remaining product purified by flash column chromatography on silica gel, with *n*-hexane/ethyl acetate as the solvent mixture.

Additional purification procedure for compound 23

Following the microwave-assisted deprotection of compound 23 under the optimized reaction conditions, the reaction mixture was subjected to the general work-up procedure described above. The resulting crude solid was subsequently suspended in diethyl ether to dissolve the triphenylmethyl-derived impurity. The suspension was then filtered, and the retained solid was collected to afford the desired product.

Synthesis of the Alcohol Precursor for TBDPS Protection and Subsequent Stability Studies

Synthesis of [3-(4-nitrophenyl)isoxazol-5-yl]methanol

The compound was synthesized via a copper(I) iodide-catalyzed 1,3-dipolar cycloaddition, following a procedure adapted from Vieira et al. (2009)[1].

Initially, 4-nitrobenzaldehyde oxime was prepared from 4-nitrobenzaldehyde (5.00 g, 33.11 mmol), hydroxylamine hydrochloride (2.80 g, 39.73 mmol), and sodium hydroxide (1.60 g, 23.83 mmol) in

an ethanol/water mixture (1:1, v/v, 66 mL) at room temperature. The resulting aldoxime was subsequently treated with *N*-chlorosuccinimide (4.40 g, 33.11 mmol) in *N,N*-dimethylformamide (133 mL) to afford the corresponding 4-nitrobenzohydroximoyl chloride, which was used in the subsequent reaction without further purification.

The crude hydroximoyl chloride was subjected to an *in situ base*-mediated elimination to generate the corresponding nitrile oxide, which underwent a 1,3-dipolar cycloaddition with propargyl alcohol (1.95 mL, 33.71 mmol) in the presence of copper(I) iodide (0.45 g, 1.44 mmol) and potassium bicarbonate (3.37 g, 33.71 mmol) in a *tert*-butanol/THF mixture (3:1, v/v, 120 mL). All reactions were performed at room temperature. The crude product was obtained in 86% yield and subsequently purified by recrystallization using solvent diffusion (ethyl acetate/hexane), affording compound I in 50% yield.

Characterization data: Yield: 50%; m.p. 162–163 °C; FT-IR (v_max, cm^{-1}): 3522, 3313, 3144, 1597, 1508, 1432, 1344, 1037; ^1H NMR (400 MHz, $CDCl_3$) δ 8.30 (d, J = 8.8 Hz, 2H), 7.96 (d, J = 8.8 Hz, 2H), 6.63 (s, 1H), 4.84 (d, J = 5.4 Hz, 2H), 2.15 (t, 1H); ^{13}C NMR (101 MHz, $CDCl_3$) δ 172.90, 160.69, 148.77, 134.98, 127.70, 124.26, 100.15, 56.65.

Synthesis of 5-(((*tert*-butyldiphenylsilyl)oxy)methyl)-3-(4-nitrophenyl)isoxazole

The TBDPS-protected derivative II was prepared following a modified literature procedure[2]. Briefly, *tert*-butyldiphenylsilyl chloride (TBDPSCl, 245 mg, 0.890 mmol) was slowly added to a solution of [3-(4-nitrophenyl)isoxazol-5-yl]methanol (I, 200 mg, 0.908 mmol) and imidazole (124 mg, 1.817 mmol) in anhydrous dichloromethane (7.8 mL) under a nitrogen atmosphere. The reaction mixture was stirred at room temperature for 4 h. Subsequently, methanol (78 µL) was added, and the mixture was stirred for an additional 10 min. The reaction mixture was then washed with water, and the organic phase was dried over anhydrous $MgSO_4$, filtered, and concentrated under reduced pressure. Yield: 98% ^1H NMR (400 MHz, $CDCl_3$) δ 8.33 (d, J = 8.9 Hz, 1H), 8.14 – 7.90 (m, 2H), 7.82 – 7.63 (m, 4H), 7.60 – 7.33 (m, 6H), 6.66 (s, 0.35H), 6.52 (s, 0.65H), 4.87 (s, 0.65H), 4.85 (s, 1.35H), 1.11 (s, 3.49H), 1.07 (s, 5.45H).

Characterization Data

L-alanine (**2**) ^1H NMR (400 MHz, D_2O) δ 3.80 (q, *J* = 7.2 Hz, 1H), 1.49 (d, *J* = 7.3 Hz, 3H). $[\alpha]_D^{25}: +2.4°$ (c= 10.0, H_2O).

L-phenylalanine (**3**) ^1H NMR (400 MHz, D_2O) δ 7.49 – 7.31 (m, 5H), 4.00 (dd, *J* = 8.0, 5.2 Hz, 1H), 3.22 (ddd, *J* = 66.0, 14.8, 8.2 Hz, 2H). $[\alpha]_D^{25}: -33°$ (c= 2.0, H_2O)

L-histidine (**4**) ^1H NMR (400 MHz, D_2O) δ 7.76 (ddd, *J* = 43.8, 15.5, 4.6 Hz, 1H), 7.04 (s, 1H), 3.97 (dd, *J* = 8.0, 4.8 Hz, 1H), 3.16 (ddd, *J* = 44.0, 15.6, 4.7 Hz, 2H).$[\alpha]_D^{20}: +11.4°$ (c= 11.0, HCl aq.)

L-lysine (**5**) ^1H NMR (400 MHz, D_2O) δ 3.79 (t, *J* = 6.1 Hz, 1H), 3.06 (t, *J* = 7.6 Hz, 2H), 2.02 – 1.84 (m, 2H), 1.75 (p, *J* = 7.7 Hz, 2H), 1.51 (dtt, *J* = 23.3, 9.4, 4.4 Hz, 2H). $[\alpha]_D^{20}: +14.6°$ (c=6.5, H_2O)

GABA (**6**) ^1H NMR (400 MHz, D_2O) δ 3.01 (t, *J* = 7.6 Hz, 2H), 2.29 (t, *J* = 7.4 Hz, 2H), 1.90 (p, *J* = 7.4 Hz, 2H).

11-Aminoundecanoic acid (**7**) ^{1}H NMR (400 MHz, D_2O) δ 3.46 (t, *J* = 7.6 Hz, 1H), 2.83 (t, *J* = 7.4 Hz, 1H), 2.08 (dp, *J* = 22.1, 6.8 Hz, 2H), 1.85 – 1.65 (m, 7H). ^{13}C NMR (101 MHz, D_2O) δ 179.29, 40.19, 34.30, 28.83, 28.80, 28.68, 28.65, 28.55, 27.15, 25.98, 24.77.

Azetidine-3-carboxylic acid (**8**) ^{1}H NMR (400 MHz, D_2O) δ 4.30 – 4.18 (m, 4H), 3.62 (p, *J* = 9.2 Hz, 1H).

Pyrrolidine-3-carboxylic acid (**9**) ^{1}H NMR (400 MHz, MeOH-d_4) δ 3.38 – 3.30 (m, 1H), 3.24 – 3.05 (m, 3H), 2.87 (p, *J* = 6.9 Hz, 1H), 2.06 (dh, *J* = 13.4, 7.0 Hz, 2H).

Piperazin-2-one (**10**) ^{1}H NMR (400 MHz, DMSO-d_6) δ 7.57 (s, 1H), 3.16 – 3.06 (m, 4H), 2.75 (t, *J* = 5.4 Hz, 2H).

N-methoxy-*N*-methylpiperidine-4-carboxamide (**11**) ^{1}H NMR (400 MHz, DMSO-d_6) δ 3.21 (s, 3H), 2.62 (s, 3H), 2.50 (t, *J* = 12.5 Hz, 2H), 2.34 – 2.21 (m, 1H), 2.04 (t, *J* = 11.3 Hz, 2H), 1.15 – 0.85 (m, 4H).

(1*R*,5*S*)-8-Azabicyclo[3.2.1]octan-3-one (**12**) ^{1}H NMR (400 MHz, $CDCl_3$) δ 3.87 (s, 2H), 2.56 (dd, *J* = 16.3, 4.5 Hz, 2H), 2.34 (d, *J* = 15.9 Hz, 2H), 1.92 – 1.85 (m, 2H), 1.73 – 1.65 (m, 2H).

1-(4-Aminophenyl)piperazin-2-one (**13**) ^{1}H NMR (400 MHz, DMSO-d_6) δ 6.88 (d, *J* = 8.6 Hz, 2H), 6.53 (d, *J* = 8.6 Hz, 2H), 5.06 (s, 2H), 3.45 (t, *J* = 5.4 Hz, 2H), 3.31 (s, 2H), 2.95 (t, *J* = 5.4 Hz, 2H).

1-(4-amino-3-fluorophenyl)piperazin-2-one (**14**) ^{1}H NMR (400 MHz, DMSO-d_6) δ 6.96 (dd, *J* = 12.7, 2.3 Hz, 1H), 6.83 – 6.70 (m, 2H), 5.12 (s, 2H), 3.49 (t, *J* = 5.4 Hz, 2H), 3.34 (s, 2H), 3.16 (s, broad, 2H), 2.97 (t, *J* = 5.4 Hz, 2H).

Aniline (**15**) ^{1}H NMR (400 MHz, DMSO-d_6) δ 7.08 – 6.98 (m, 1H), 6.64 – 6.52 (m, 2H), 4.48 – 4.06 (m, 2H).

4-Aminophenol (**16**) ^{1}H NMR (400 MHz, DMSO-d_6) δ 8.31 (s, 1H), 6.47 (d, *J* = 8.6 Hz, 2H), 6.41 (d, *J* = 8.7 Hz, 2H), 4.36 (s, 2H).

4-Chloroaniline (**17**) ^{1}H NMR (400 MHz, $CDCl_3$) δ 7.14 – 7.06 (m, 2H), 6.64 – 6.56 (m, 2H), 3.65 (s, 2H).

4-Methylaniline (**18**) ^{1}H NMR (400 MHz, DMSO-d_6) δ 6.81 (d, *J* = 7.9 Hz, 2H), 6.46 (d, *J* = 8.2 Hz, 2H), 4.75 (s, 2H), 2.12 (s, 3H).

4-Aminobenzoic acid (**19**) ^{1}H NMR (400 MHz, DMSO-d_6) δ 11.93 (s, 1H), 7.61 (d, *J* = 8.6 Hz, 2H), 6.54 (d, *J* = 8.5 Hz, 2H), 5.85 (s, 2H).

Methyl 6-aminonicotinate (**20**) ^{1}H NMR (400 MHz, DMSO-d_6) δ 8.50 (d, *J* = 2.3 Hz, 1H), 7.81 (dd, *J* = 8.8, 2.2 Hz, 1H), 6.81 (s, 2H), 6.45 (d, *J* = 8.7 Hz, 1H), 3.75 (s, 3H).

4-Methylbenzenesulfonamide (**21**) ^{1}H NMR (400 MHz, DMSO-d_6) δ 7.71 (d, *J* = 8.0 Hz, 2H), 7.36 (d, *J* = 8.0 Hz, 2H), 7.26 (s, 2H), 2.37 (s, 3H).

N-(2-fluoro-4-(2-oxopiperazin-1-yl)phenyl)-2-(4-phenyl-1*H*-1,2,3-triazol-1-yl)acetamide (**22**) ^{1}H NMR (400 MHz, DMSO-d_6) δ 10.52 (s, 1H), 9.85 (s broad, 1H), 8.61 (s, 1H), 7.94 (t, *J* = 8.7 Hz, 1H), 7.88 (d, *J* = 7.6 Hz, 2H), 7.47 (t, *J* = 7.6 Hz, 2H), 7.41 – 7.31 (m, 2H), 7.19 (d, *J* = 8.8 Hz, 1H), 5.50 (s, 2H), 3.90 (t, *J* = 5.6 Hz, 2H), 3.85 (s, 2H), 3.51 (t, *J* = 5.6 Hz, 2H). ^{13}C NMR (101 MHz, DMSO-d_6) δ

165.40, 162.70, 153.56 (d, J = 245.0 Hz), 146.68, 138.79 (d, J = 9.8 Hz), 131.16, 129.41, 128.35, 125.62, 124.53 (d, J = 12.1 Hz), 124.45 (d, J = 2.4 Hz), 123.56, 122.14 (d, J = 2.7 Hz), 113.98 (d, J = 21.9 Hz), 52.57, 46.60, 45.62, 40.51. ^{19}F NMR (376 MHz, DMSO-d_6) δ -122.77.

2-(2-Amino-3-(1*H*-imidazol-4-yl)propanamido)-2-methylpropanoic acid (**23**) ^{1}H NMR (400 MHz, D_2O) δ 8.71 (s, 1H), 7.37 (s, 1H), 4.58 (t, J = 4.7 Hz, 1H), 3.44 (dd, J = 15.7, 4.4 Hz, 1H), 3.24 (dd, J = 15.5, 4.7 Hz, 1H), 1.44 (s, 3H), 1.08 (s, 3H).

2-(3-(4-Chlorophenyl)isoxazol-5-yl)-*N*-(2-fluoro-4-(2-oxopiperazin-1-yl)phenyl)acetamide (**24**) ^{1}H NMR (400 MHz, DMSO-d_6) δ 10.20 (s, 1H), 7.89 (t, J = 9.0 Hz, 3H), 7.58 (d, J = 8.1 Hz, 2H), 7.35 (d, J = 12.7 Hz, 1H), 7.16 (d, J = 8.8 Hz, 1H), 6.99 (s, 1H), 4.12 (s, 2H), 3.60 (t, J = 5.4 Hz, 2H), 3.39 (s, 2H), 3.01 (t, J = 5.4 Hz, 2H). ^{13}C NMR (100 MHz, DMSO-d_6) δ 168.19, 165.71, 162.15, 161.03, 154.43, 151.98, 138.30, 138.20, 134.83, 129.20, 128.36, 127.48, 124.45, 124.34, 124.29, 122.27, 121.65, 121.62, 113.59, 113.37, 101.55, 46.10, 45.04, 34.17.

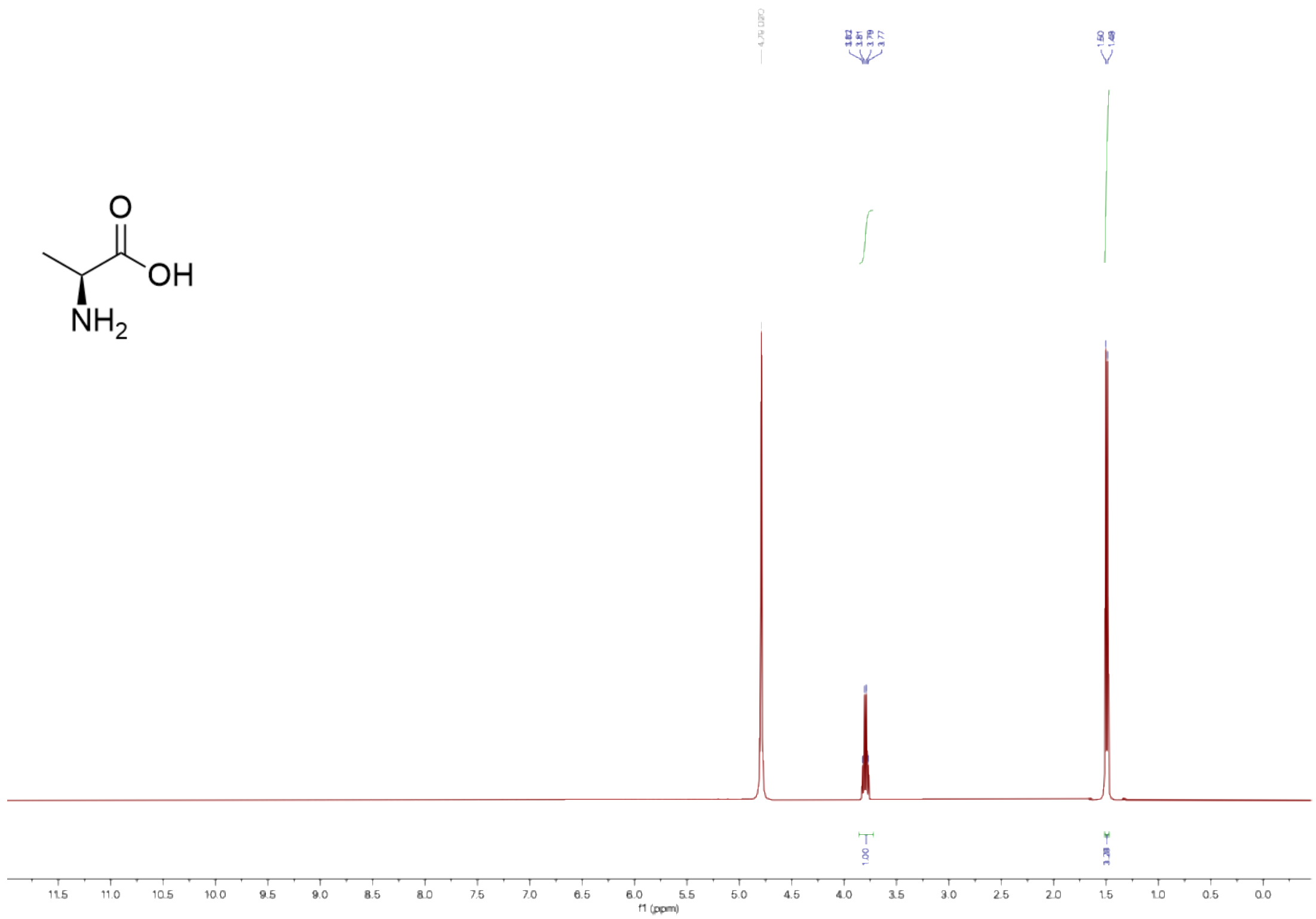

Figure 1. ^{1}H-NMR spectrum of *L*-alanine (**2**) in D_2O

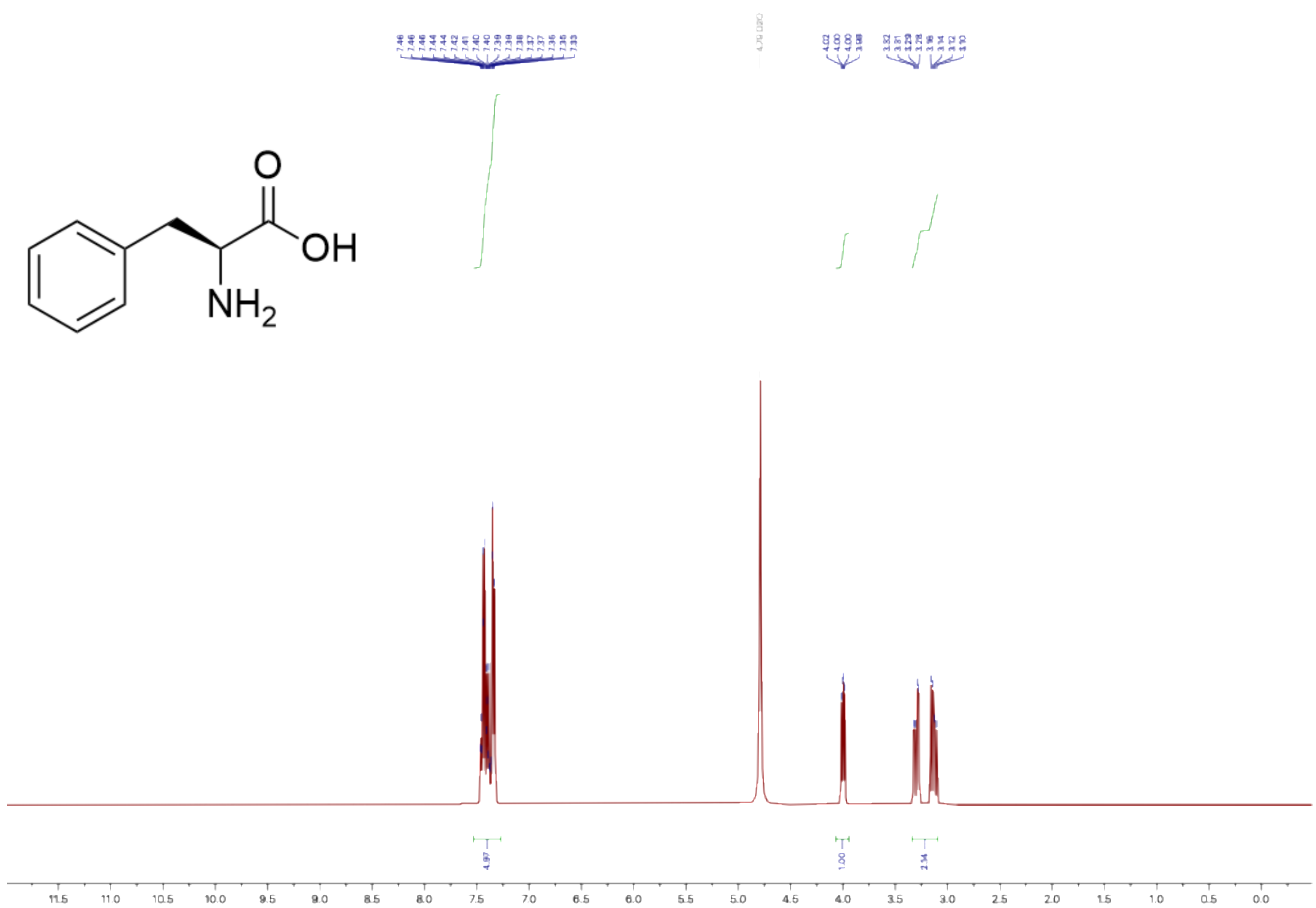

Figure 2. ^{1}H-NMR spectrum of *L*-phenylalanine (**3**) in D_2O

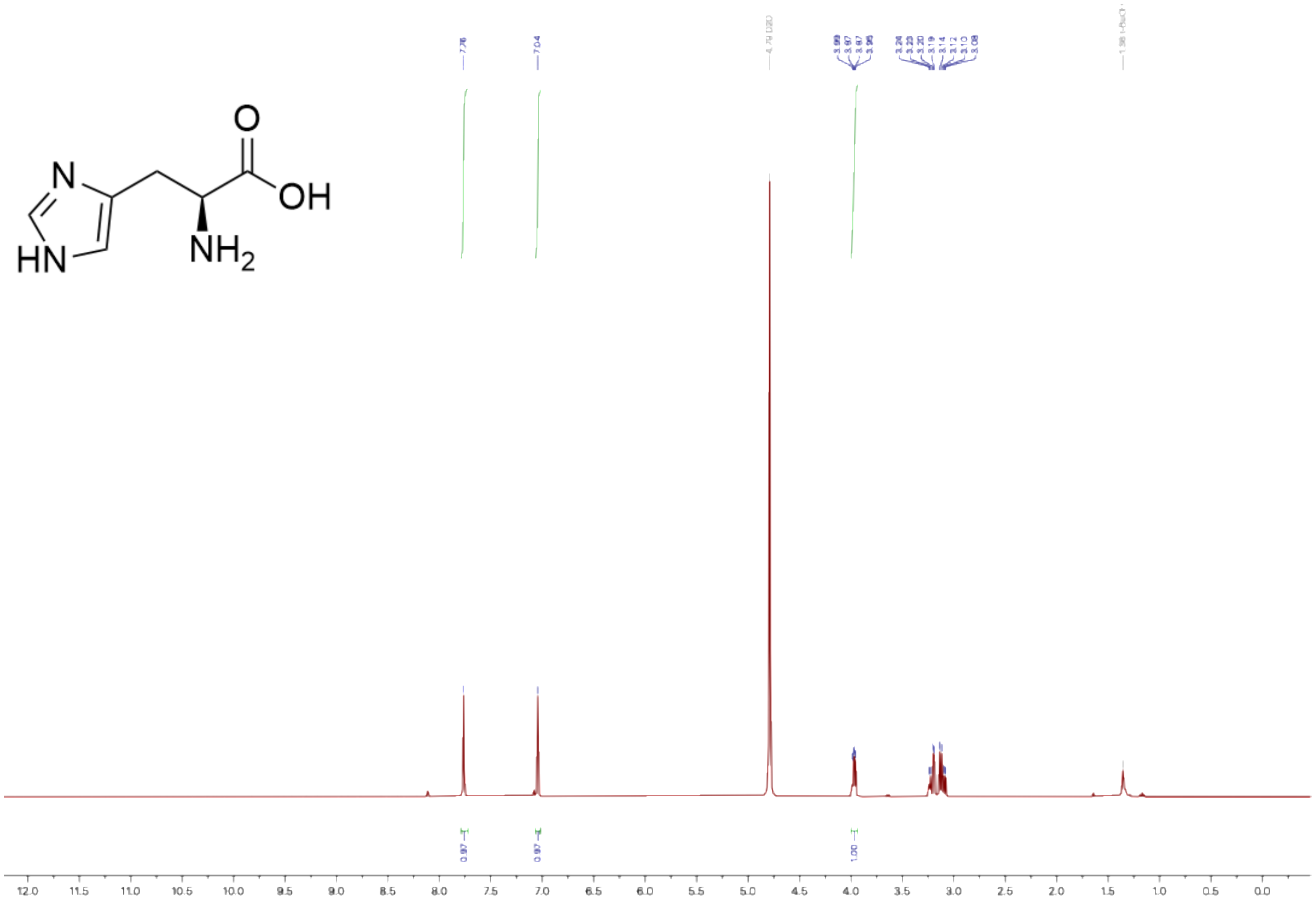

Figure 3. ^{1}H-NMR spectrum of *L*-histidine (**4**) in D_2O

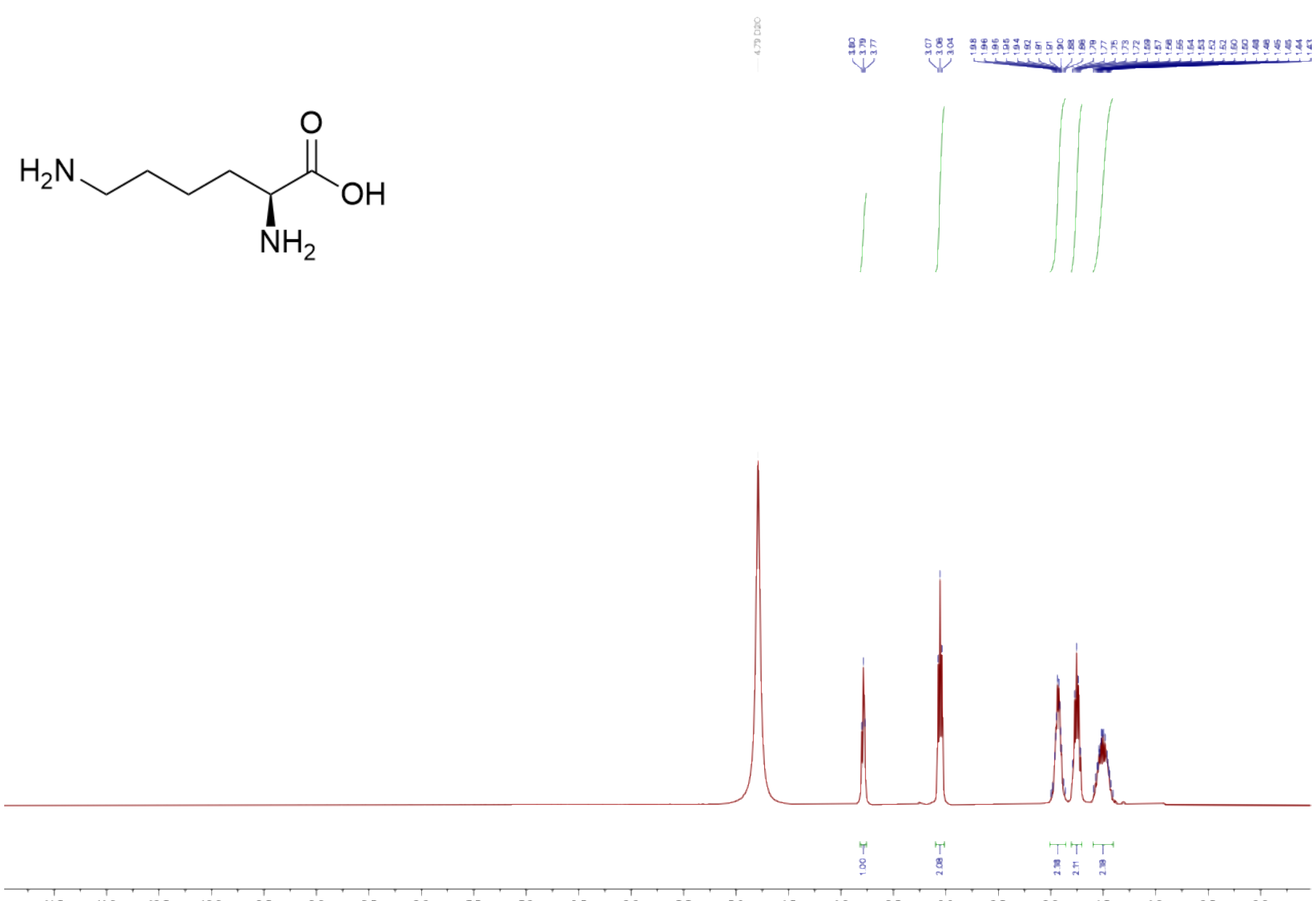

Figure 4. ^{1}H-NMR spectrum of *L*-lysine (**5**) in D_2O

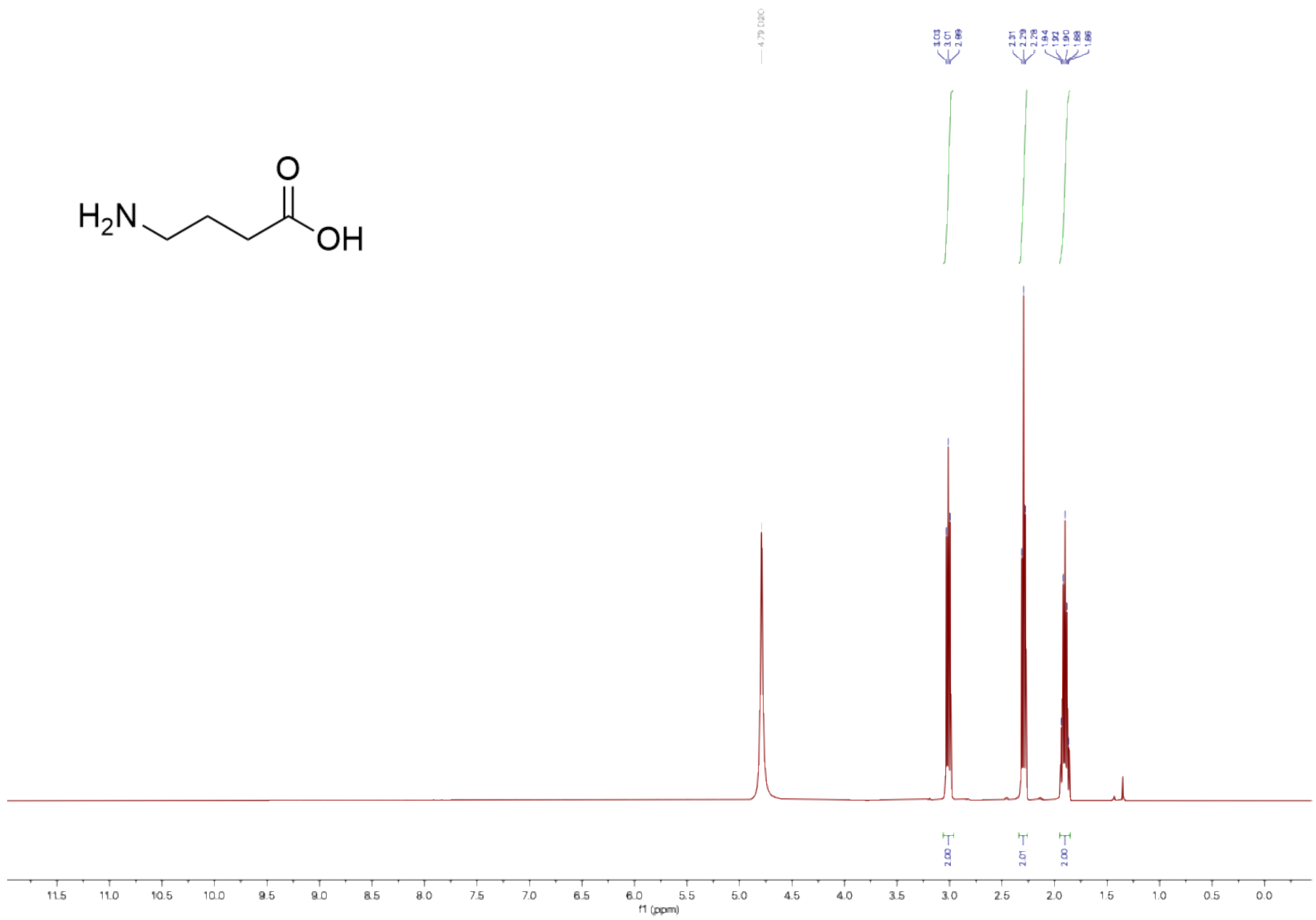

Figure 5. ^{1}H-NMR spectrum of GABA (**6**) in D_2O

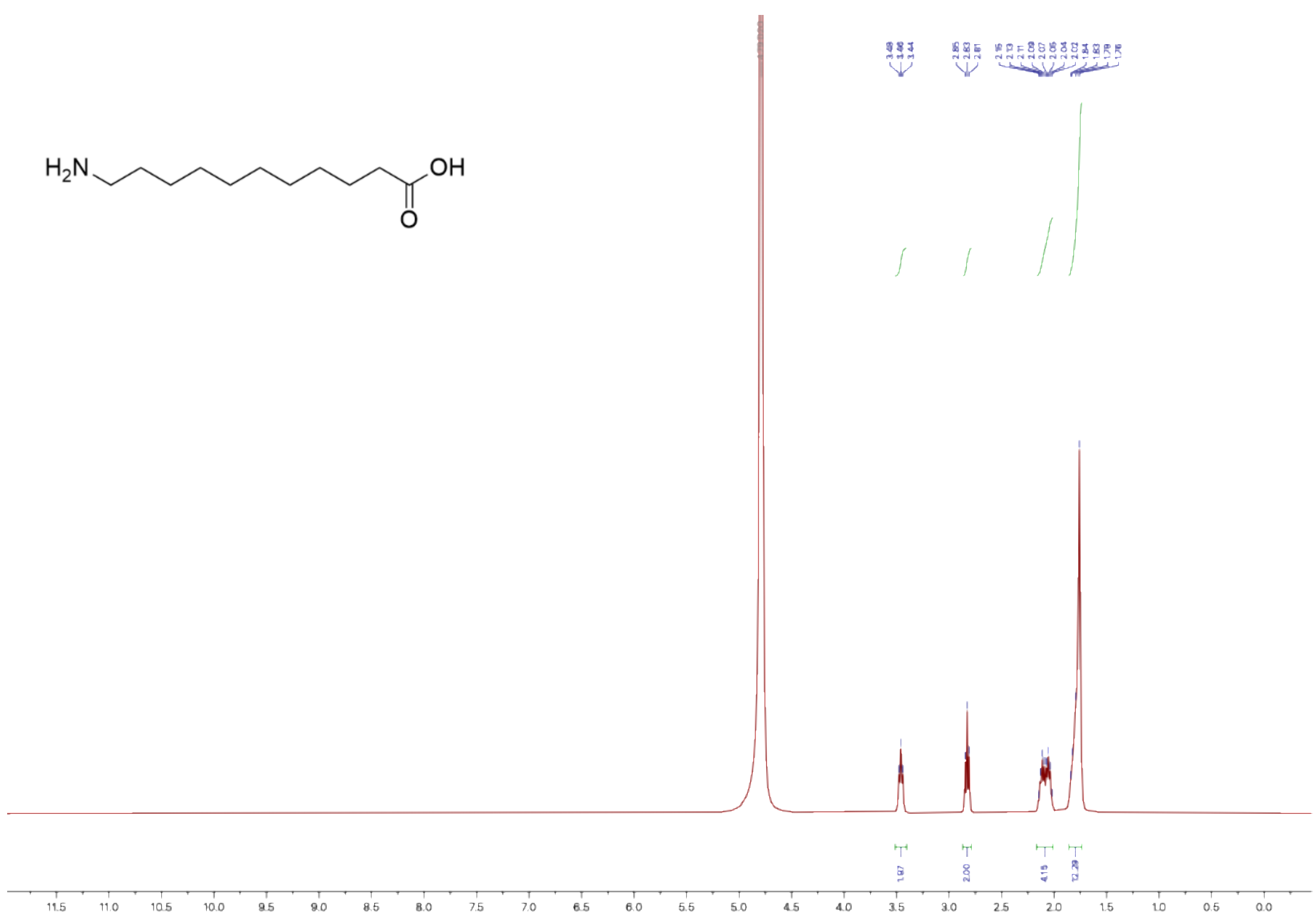

Figure 6. ^{1}H-NMR spectrum of 11-aminoundecanoic acid (**7**) hydrochloride salt in D_2O

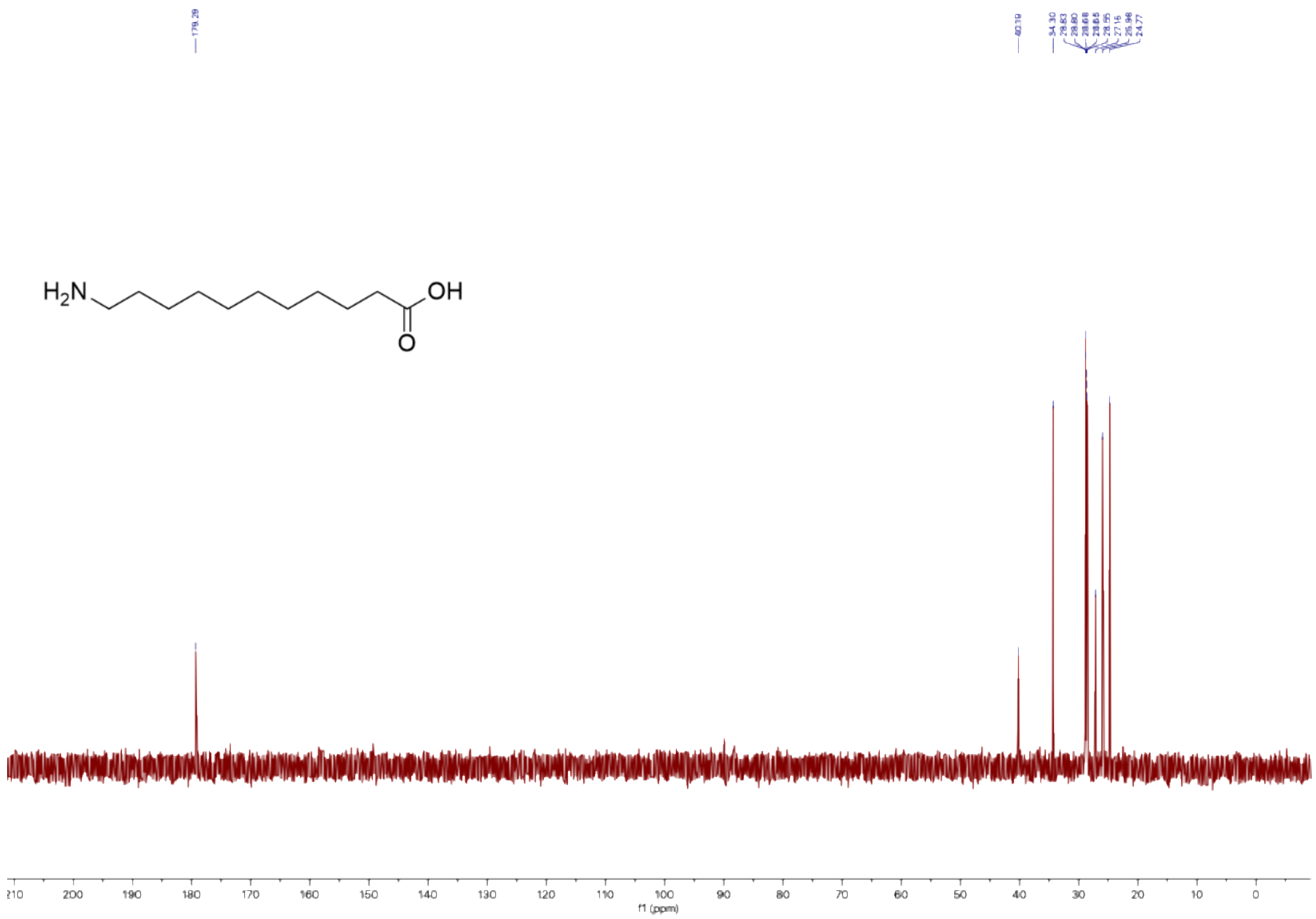

Figure 7. ^{13}C-NMR spectrum of 11-aminoundecanoic acid (**7**) hydrochloride salt in D_2O

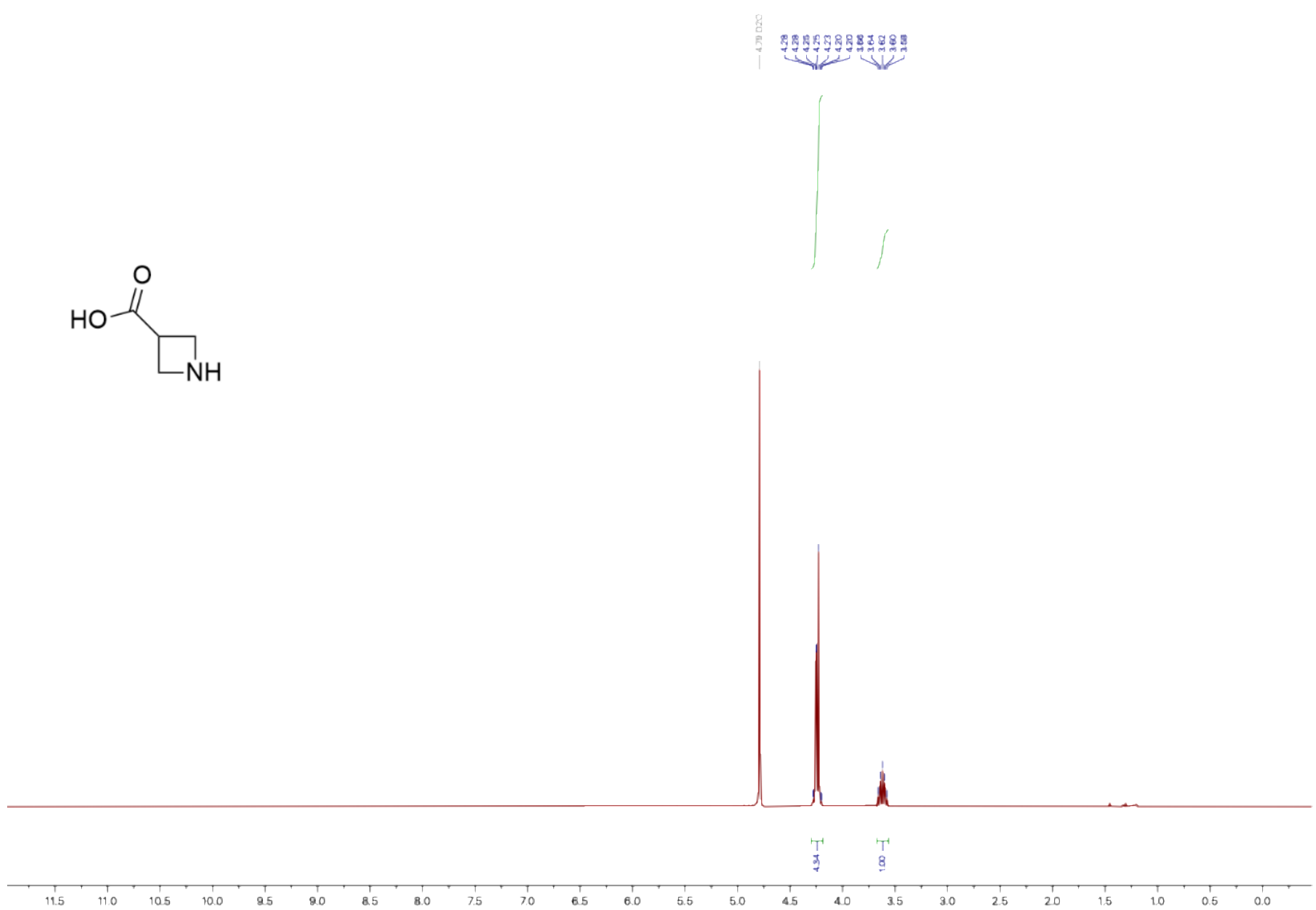

Figure 8. ^{1}H-NMR spectrum of azetidine-3-carboxylic acid (**8**) in D_2O

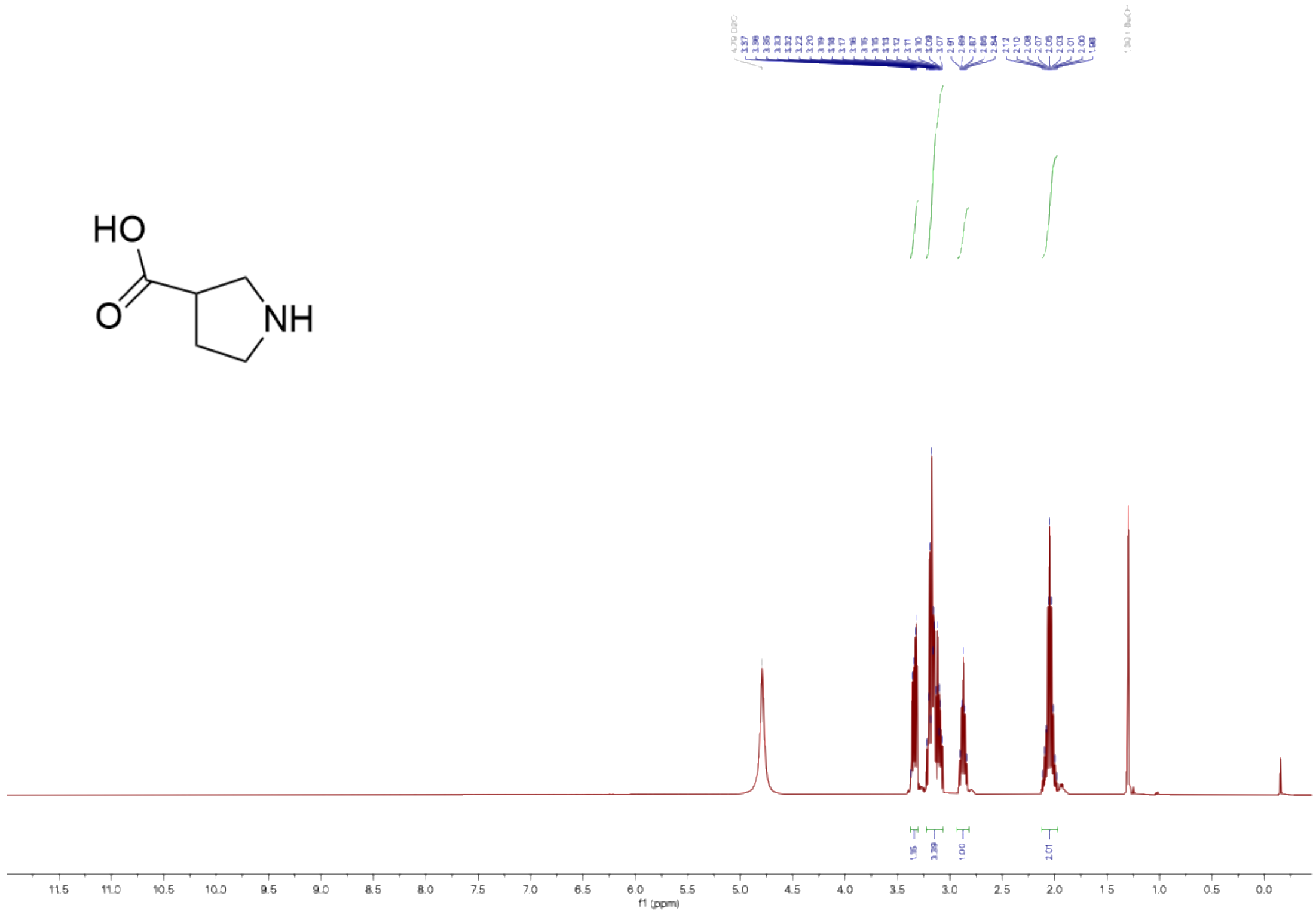

Figure 9. ^{1}H-NMR spectrum of pyrrolidine-3-carboxylic acid (**9**) in D_2O

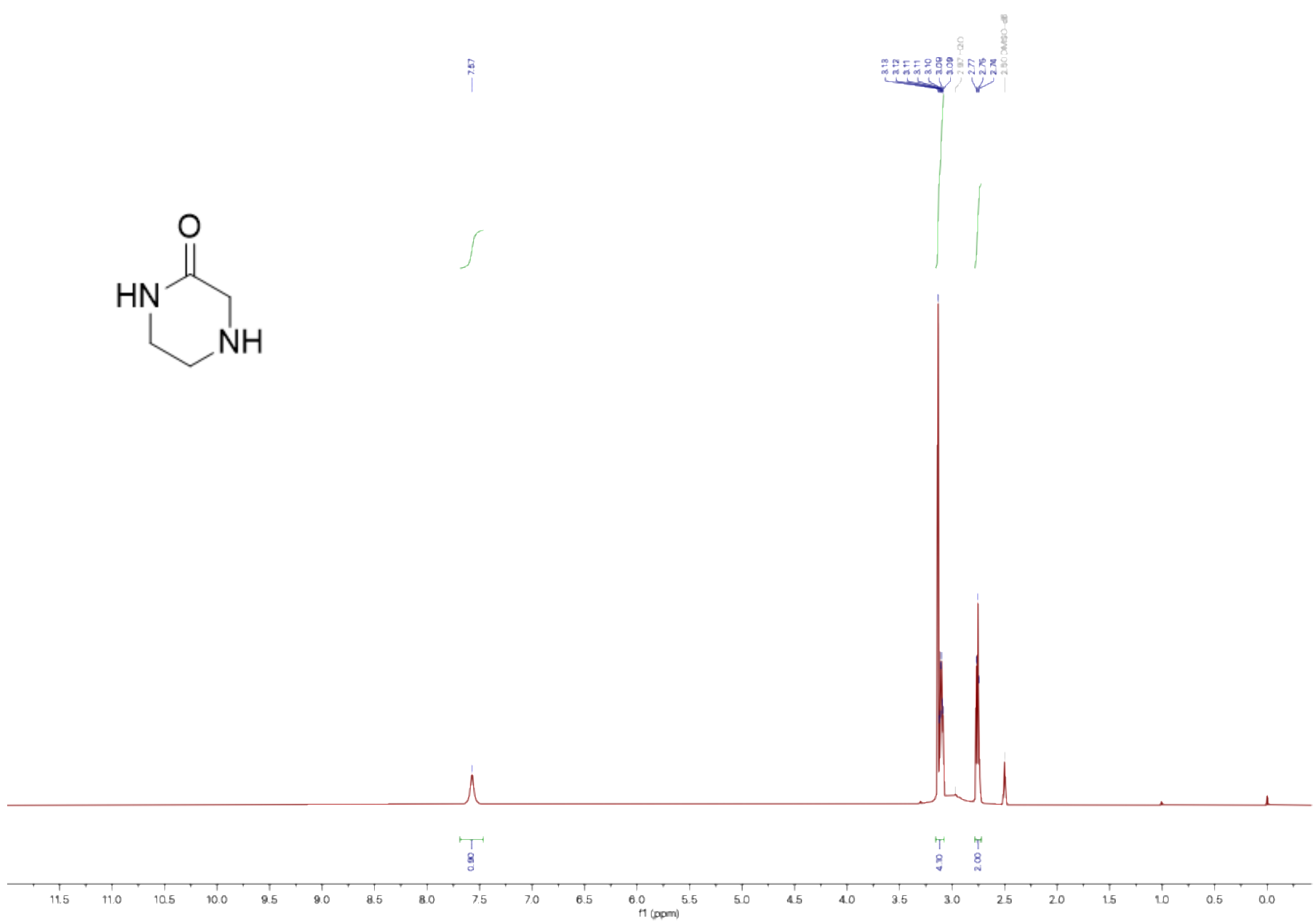

Figure 10. ^{1}H-NMR spectrum of piperazin-2-one (**10**) in DMSO-d_6

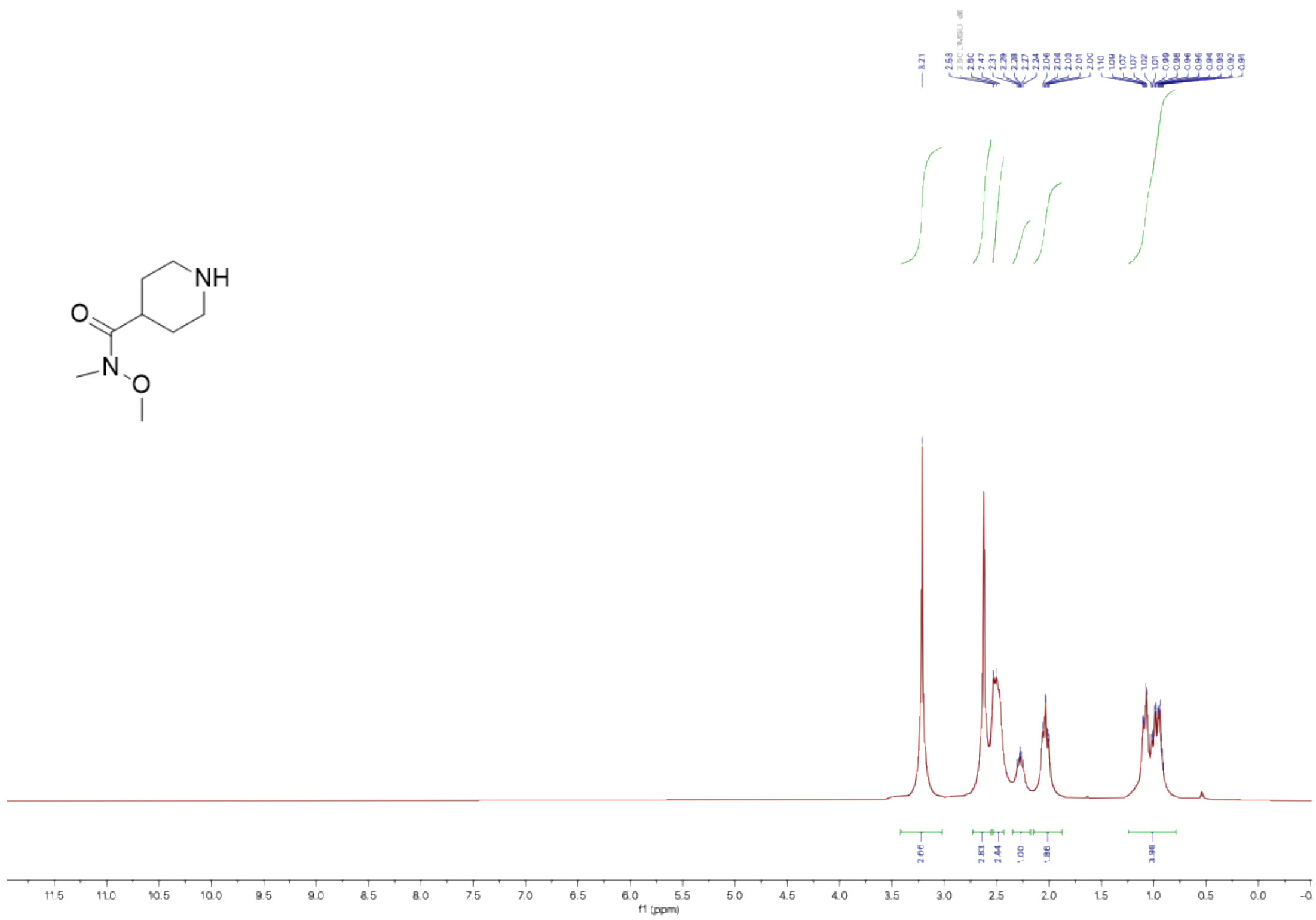

Figure 11. ^{1}H-NMR spectrum of *N*-methoxy-*N*-methylpiperidine-4-carboxamide (**11**) in DMSO-d_6

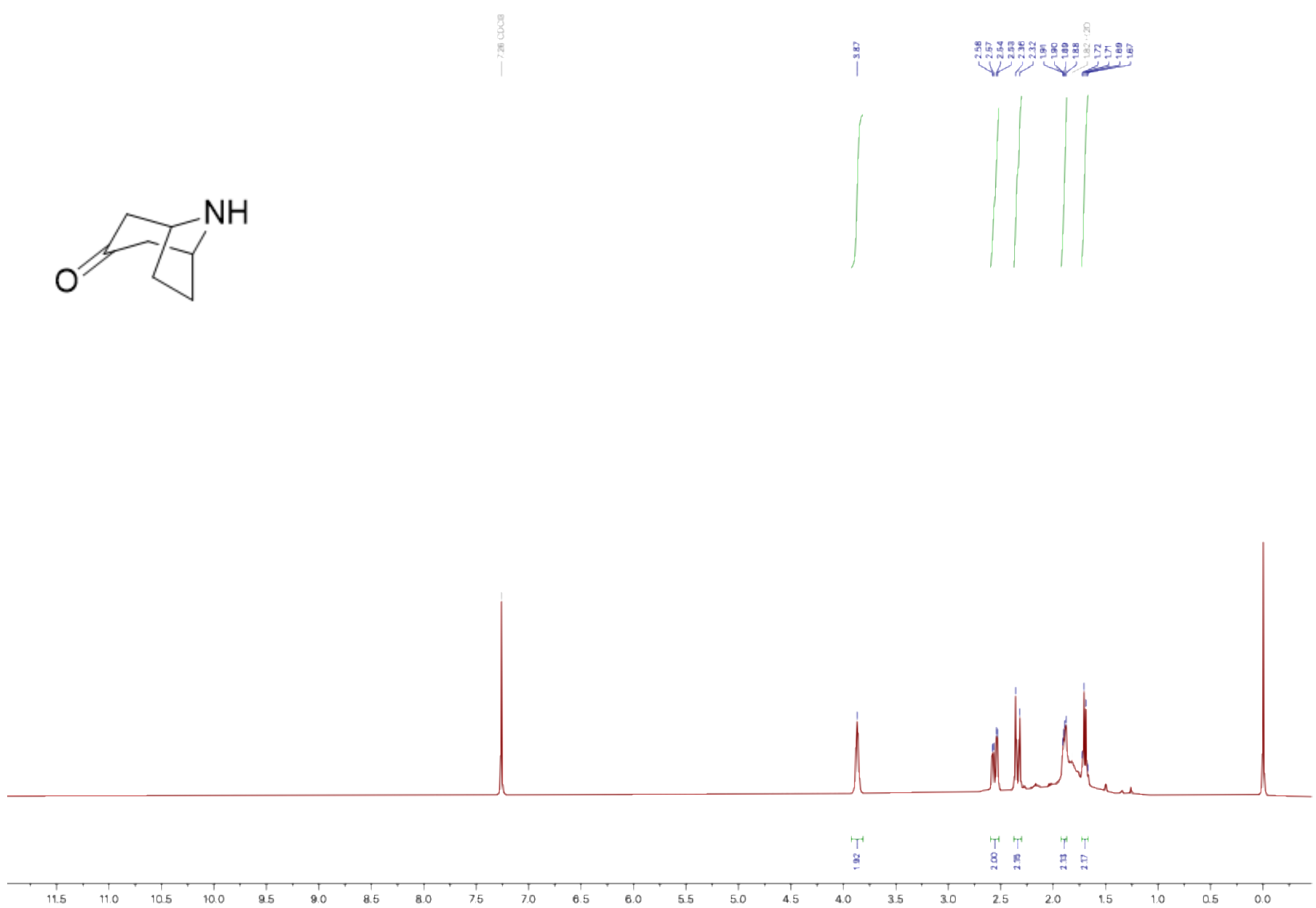

Figure 12. ^{1}H-NMR spectrum of (1*R*,5*S*)-8-azabicyclo[3.2.1]octan-3-one (**12**) in $CDCl_3$

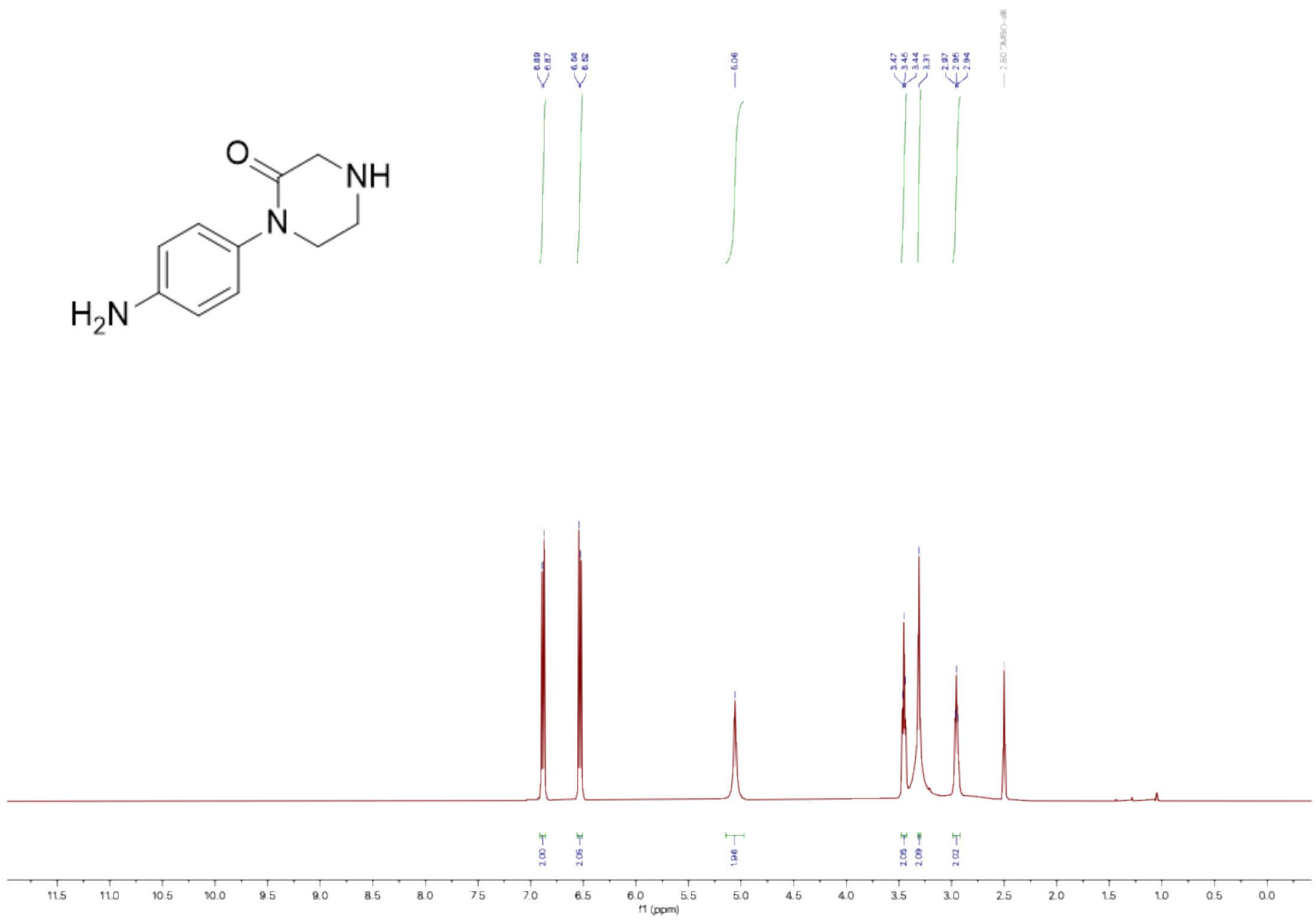

Figure 13. ^{1}H-NMR spectrum of 1-(4-aminophenyl)piperazin-2-one (**13**)

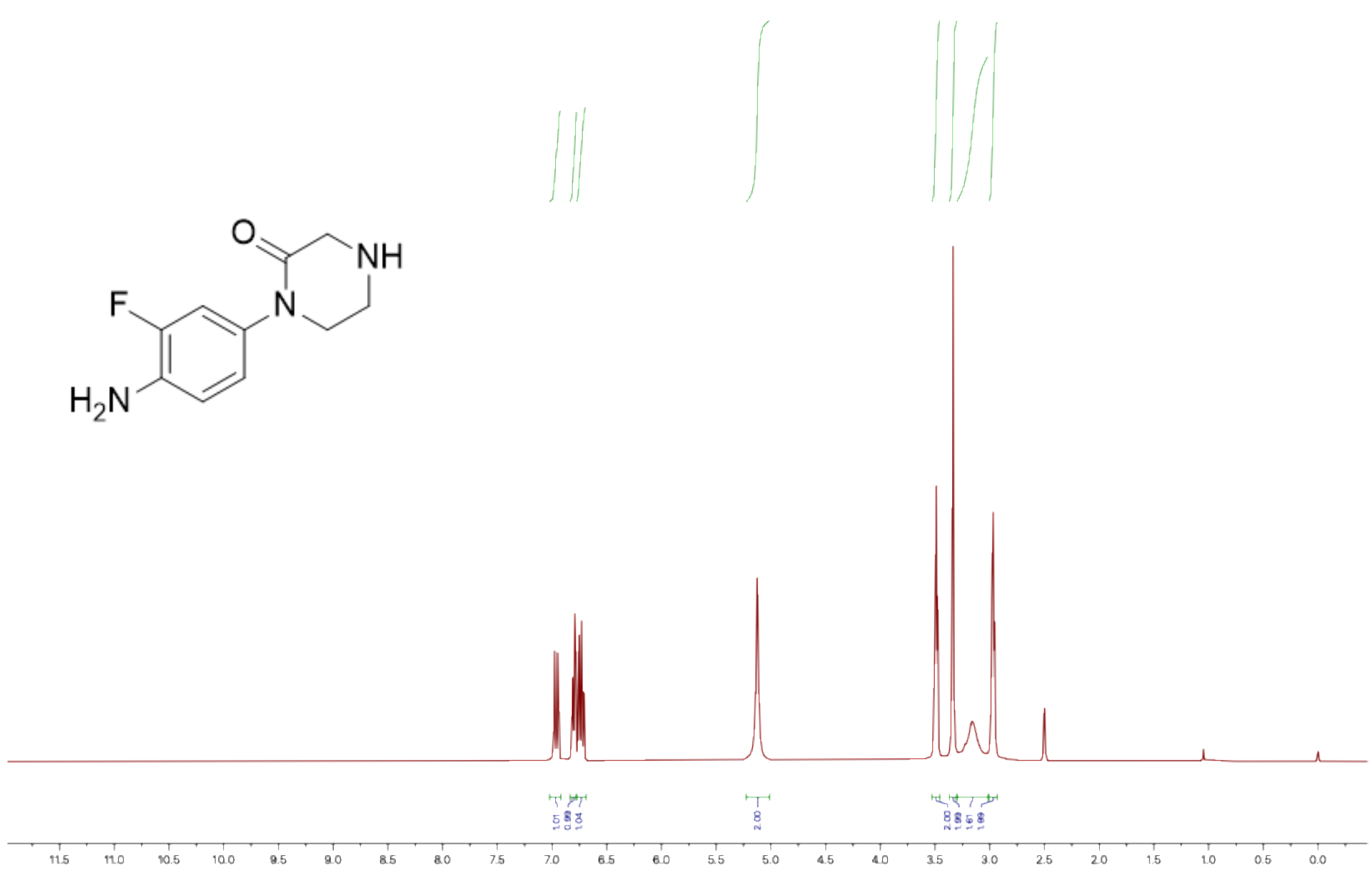

Figure 14. 1H-NMR spectrum of 1-(4-amino-3-fluorophenyl)piperazin-2-one (**14**)

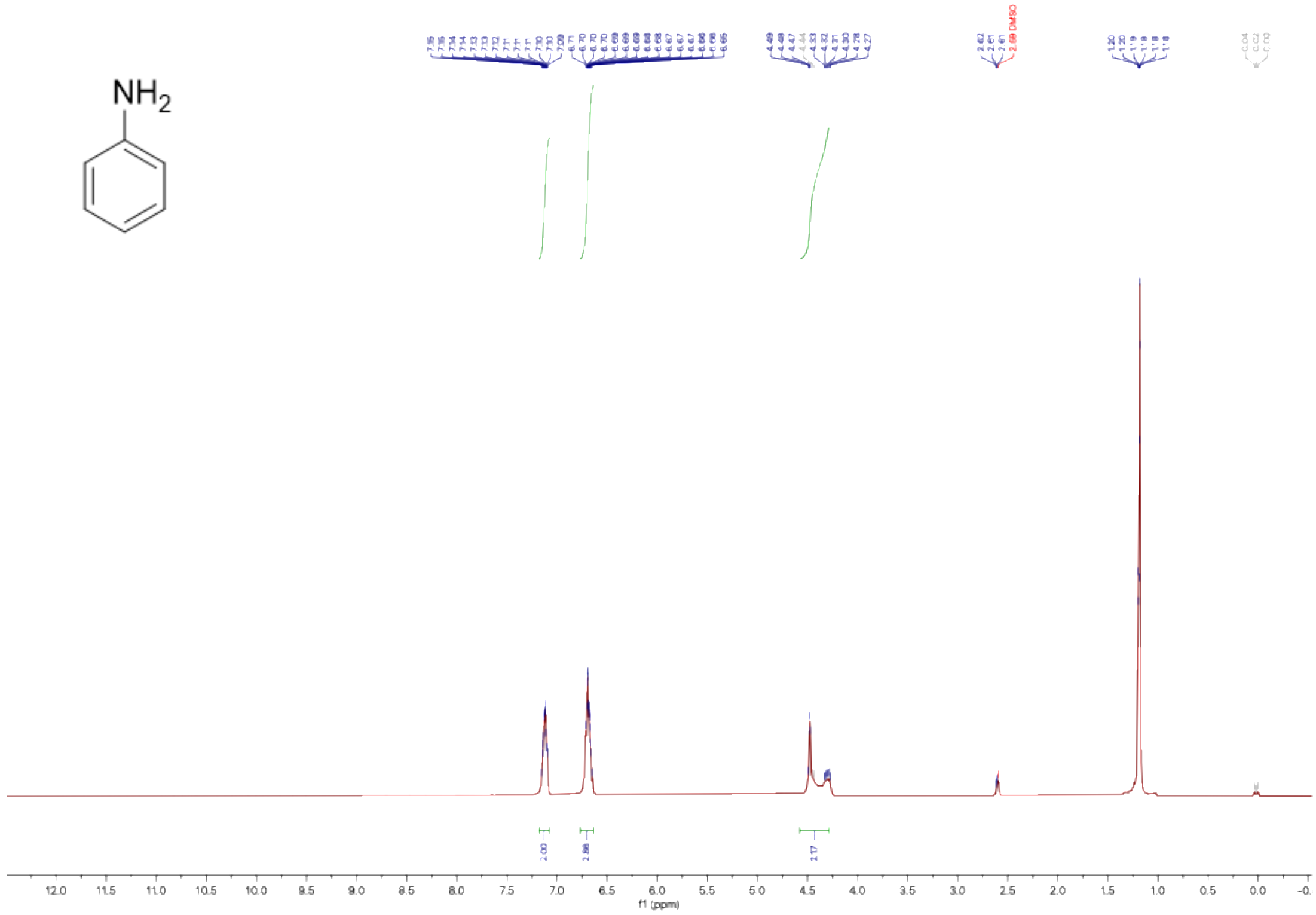

Figure 15. ^{1}H-NMR spectrum of aniline (**15**) in DMSO-d_6

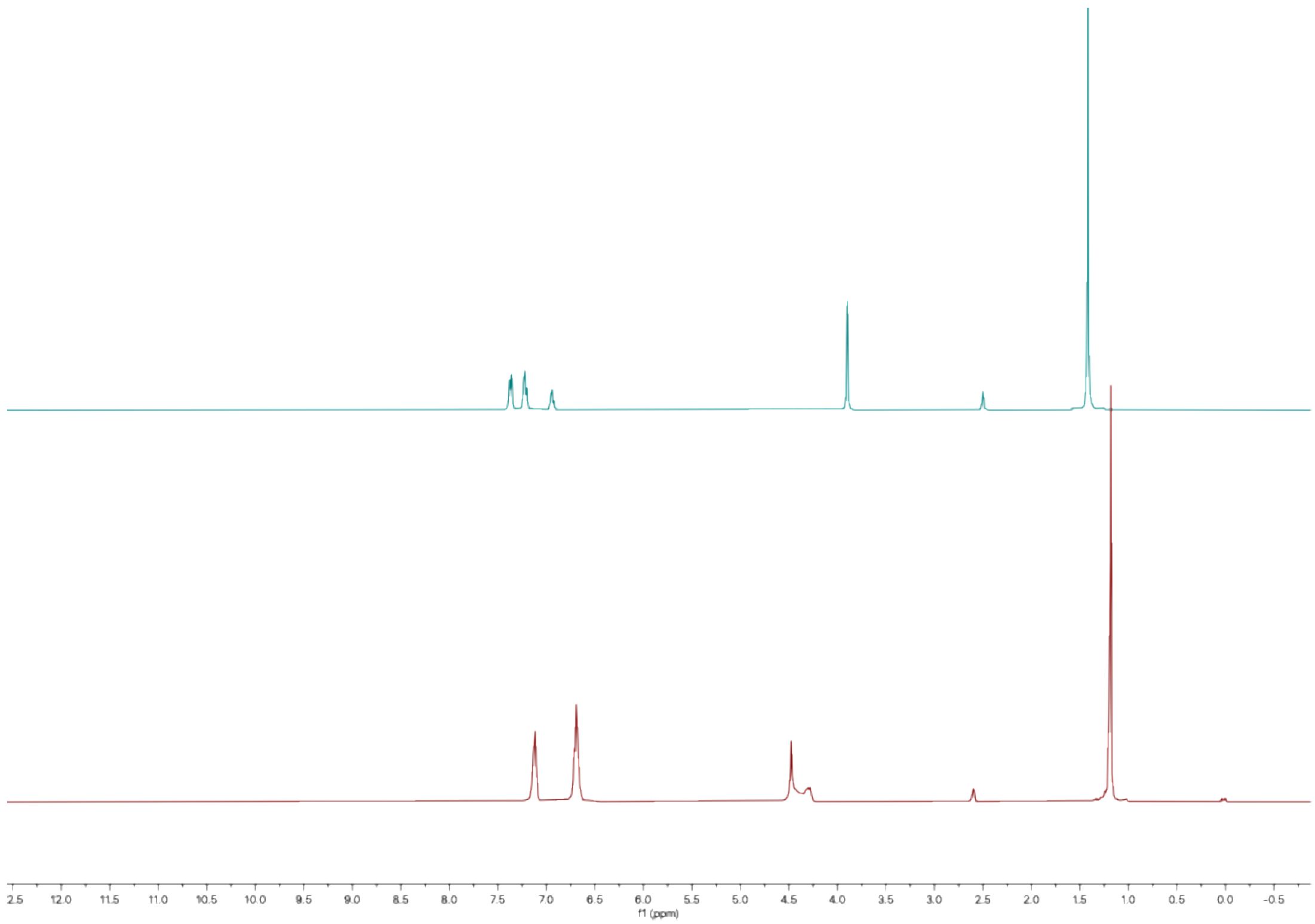

Figure 16. Stacked ^{1}H-NMR spectrum of *N*-Boc aniline (top) and aniline (**15**) (bottom)

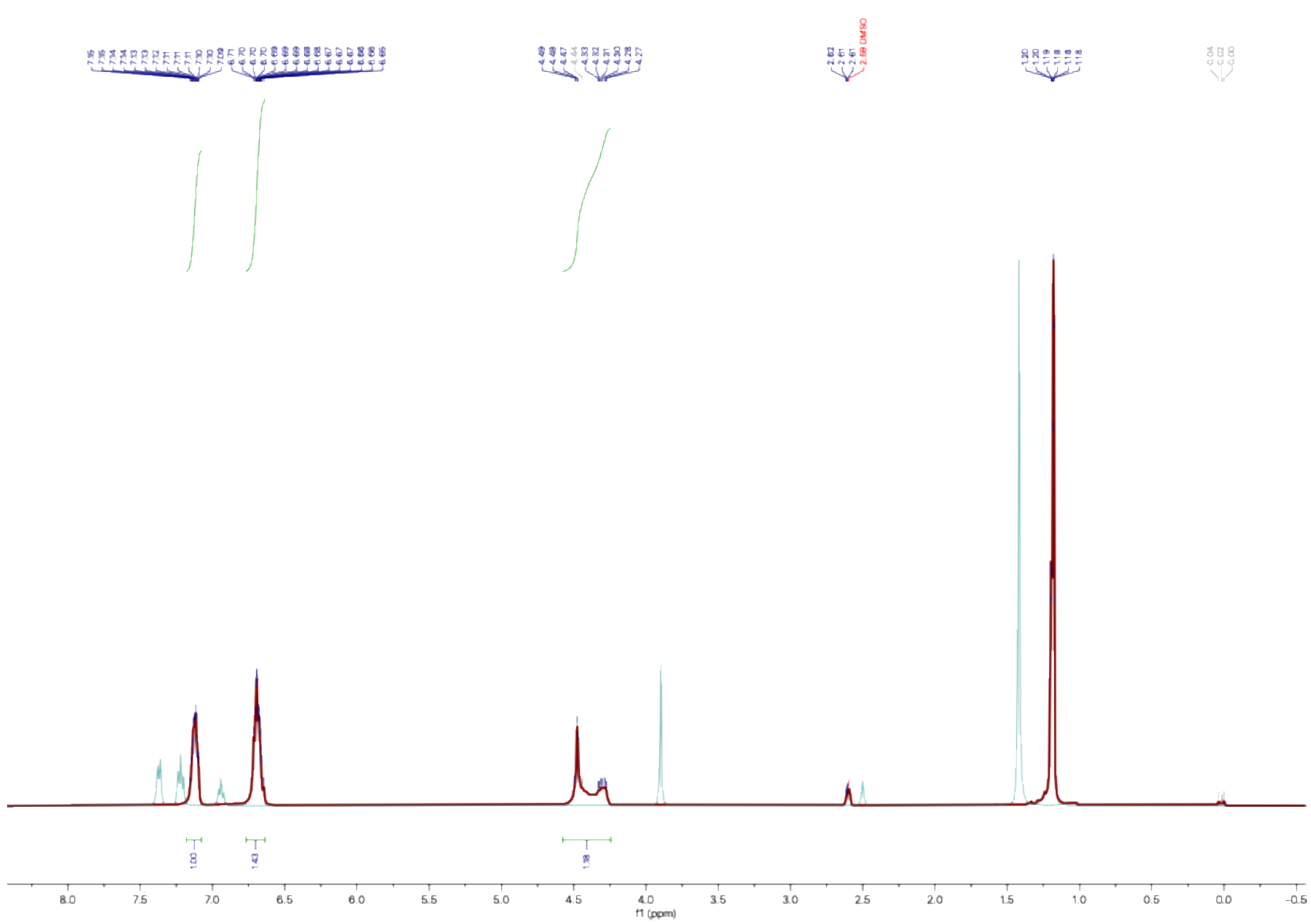

Figure 17. Superimposed ^{1}H-NMR spectrum of *N*-Boc aniline (Red) and aniline (**15**) (blue)

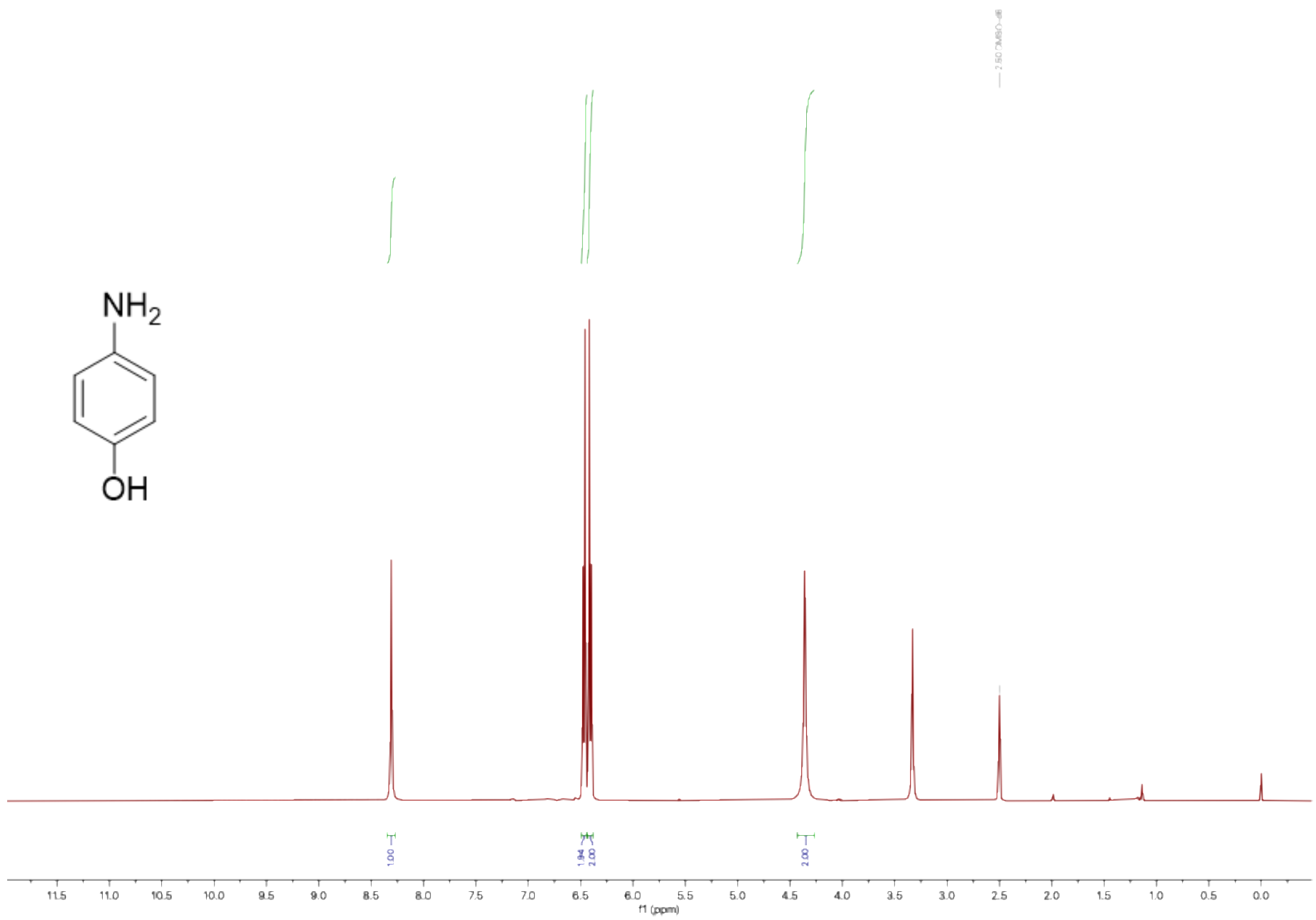

Figure 18. ^{1}H-NMR spectrum of 4-aminophenol (**16**) in DMSO-d_6

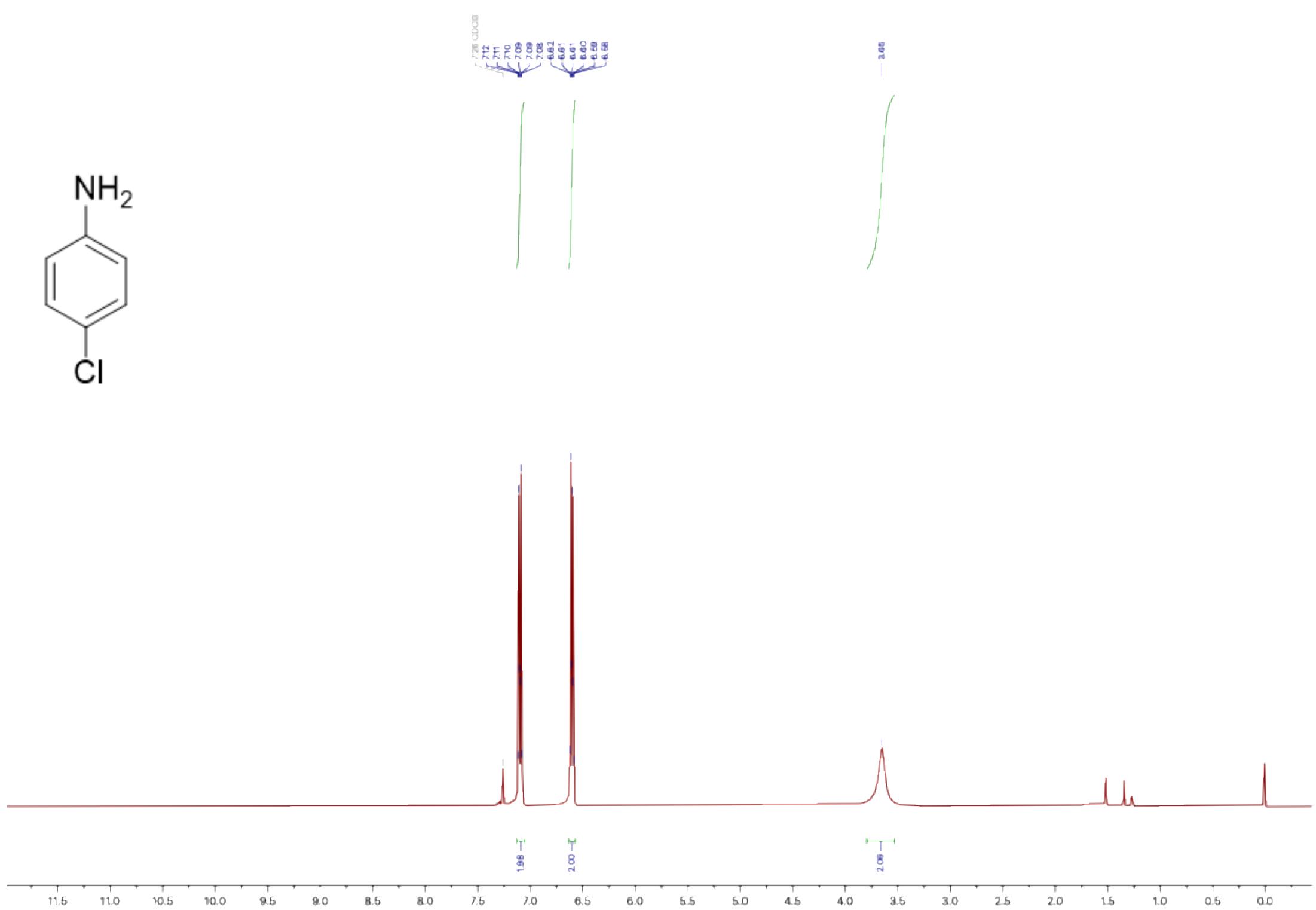

Figure 19. ^{1}H-NMR spectrum of 4-chloroaniline (**17**) in $CDCl_3$

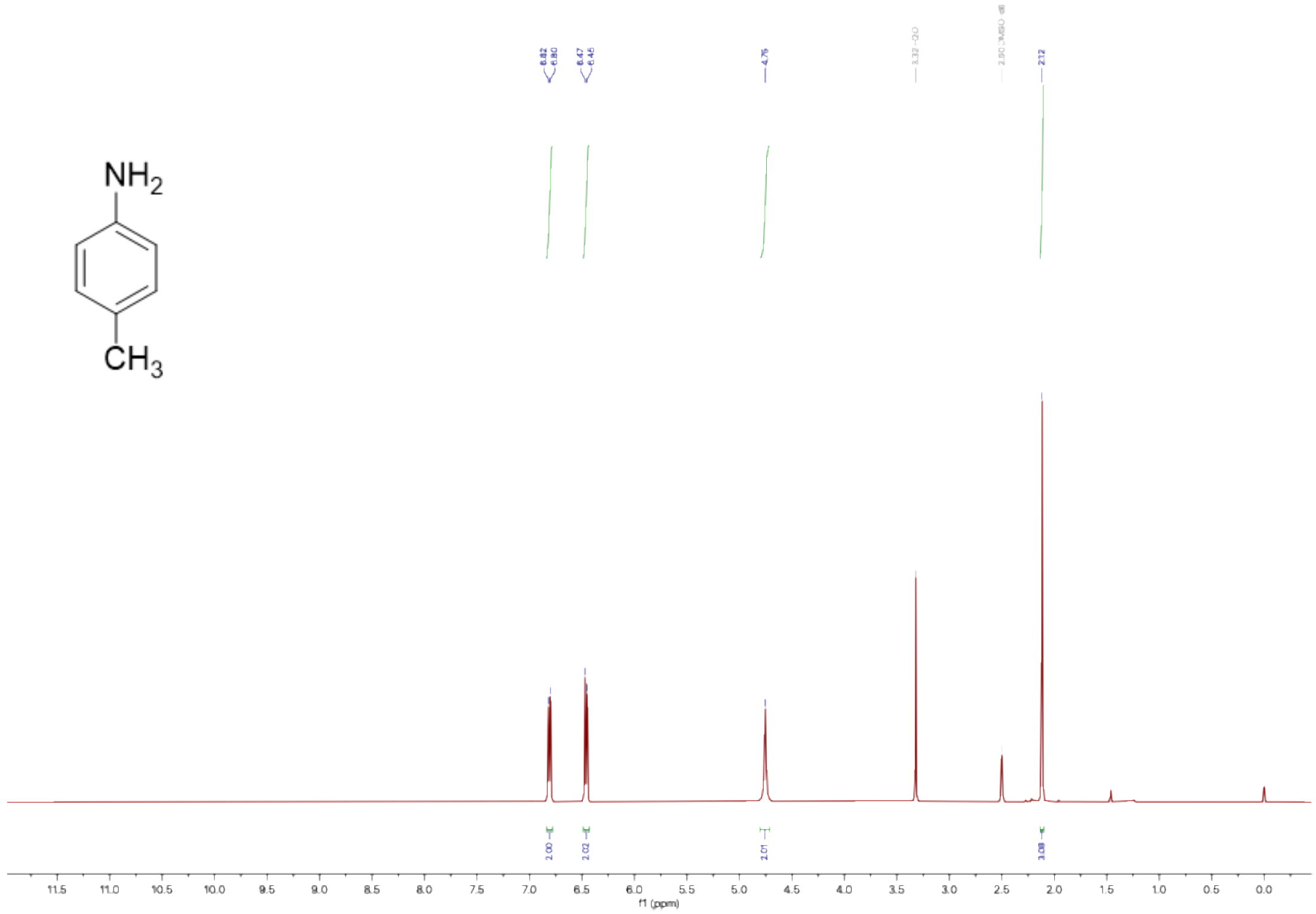

Figure 20. ^{1}H-NMR spectrum of 4-methylaniline (**18**) in DMSO-d_6

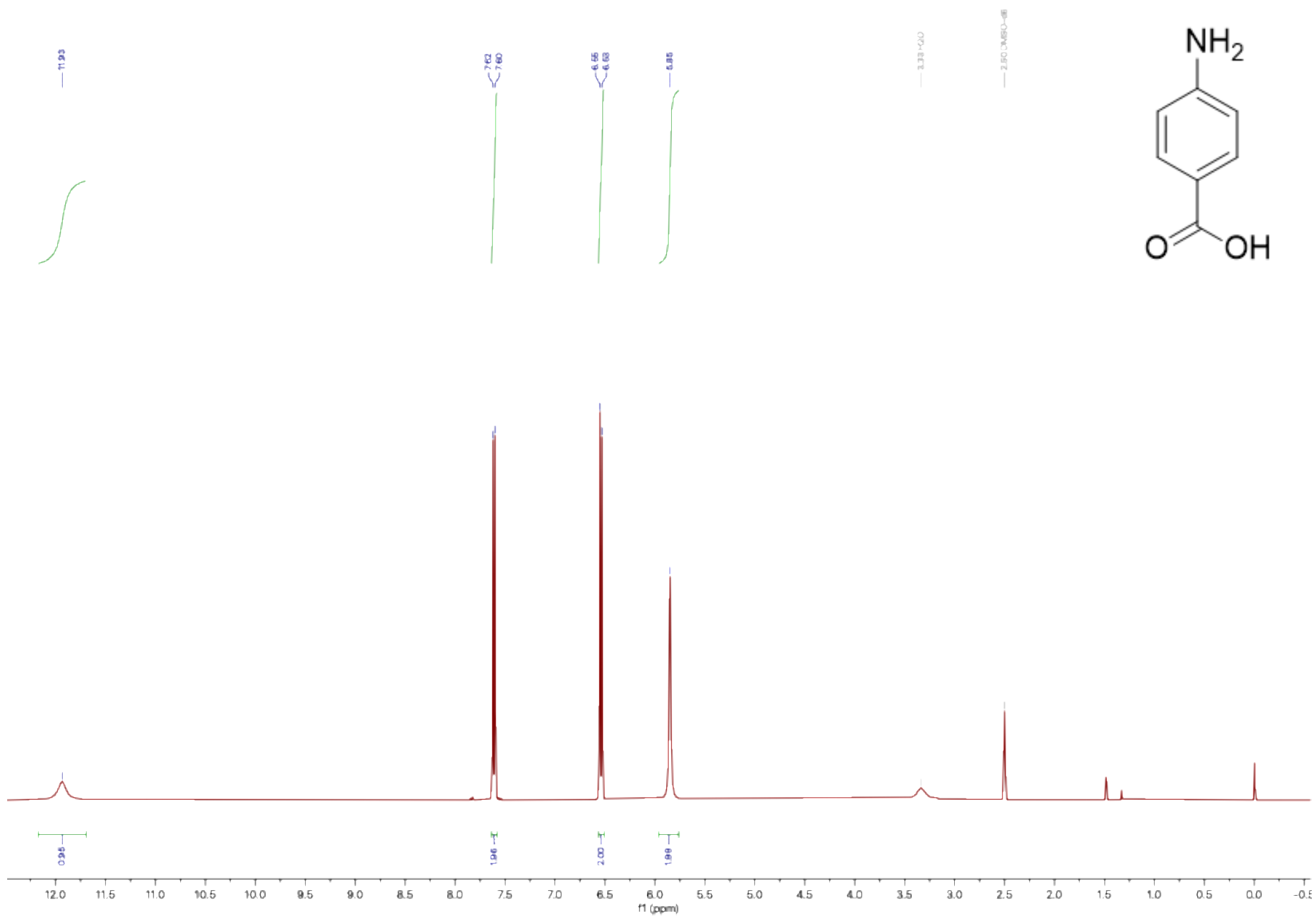

Figure 21. ^{1}H-NMR spectrum of 4-aminobenzoic acid (**19**) in DMSO-d_6

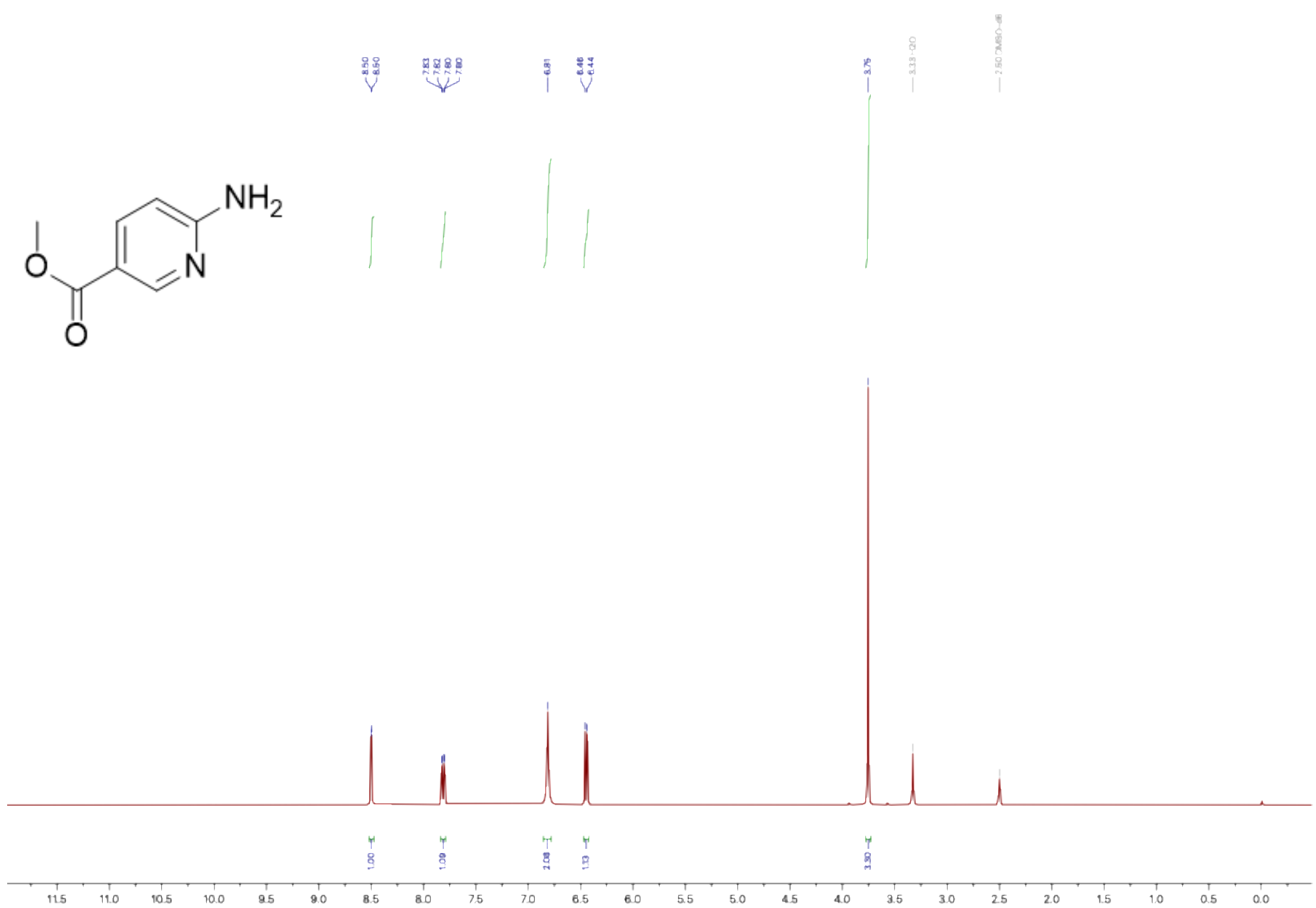

Figure 22. ^{1}H-NMR spectrum of Methyl 6-aminonicotinate (**20**) in DMSO-d_6

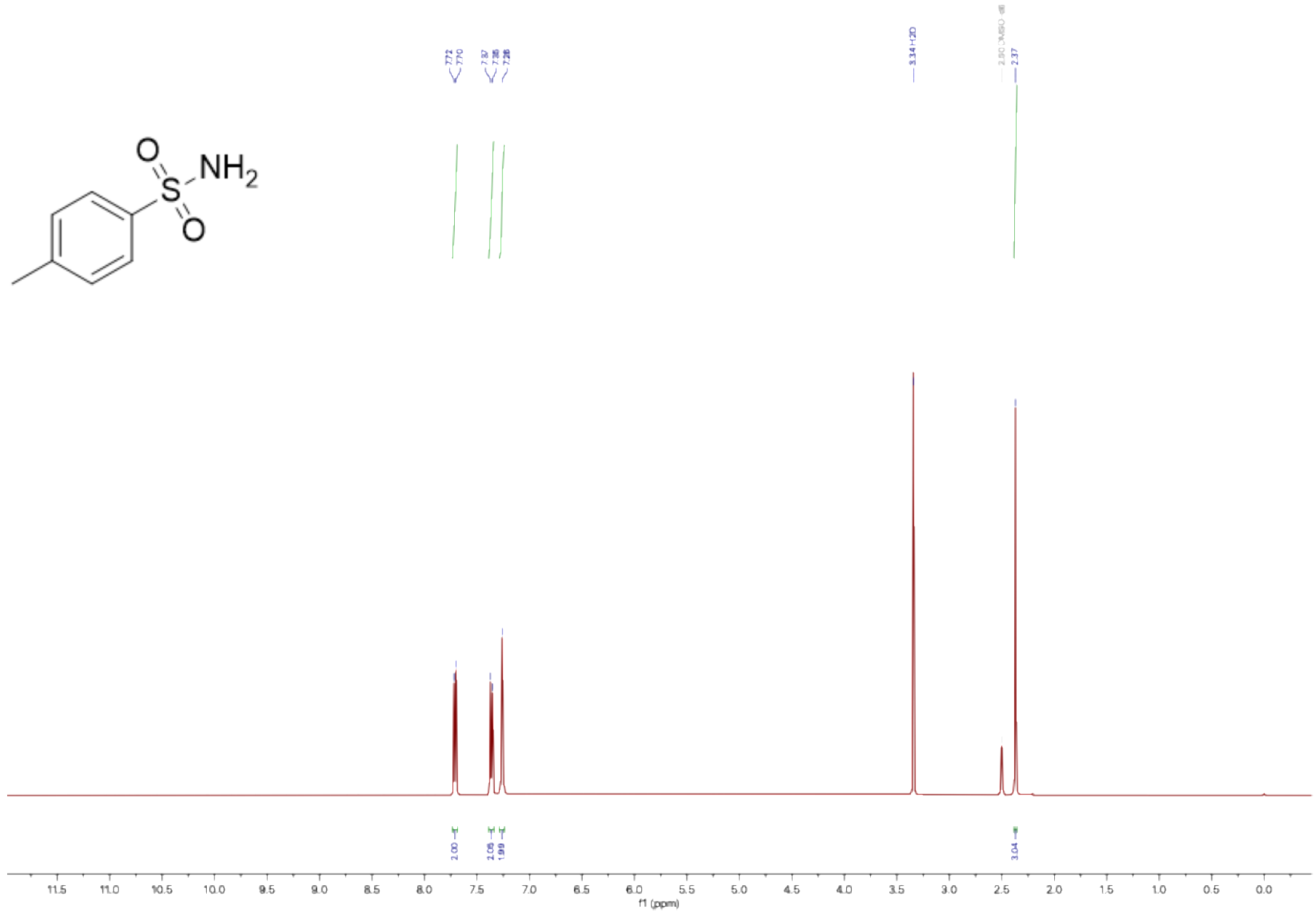

Figure 23. ^{1}H-NMR spectrum of 4-methylbenzenesulfonamide (**21**) in DMSO-d_6

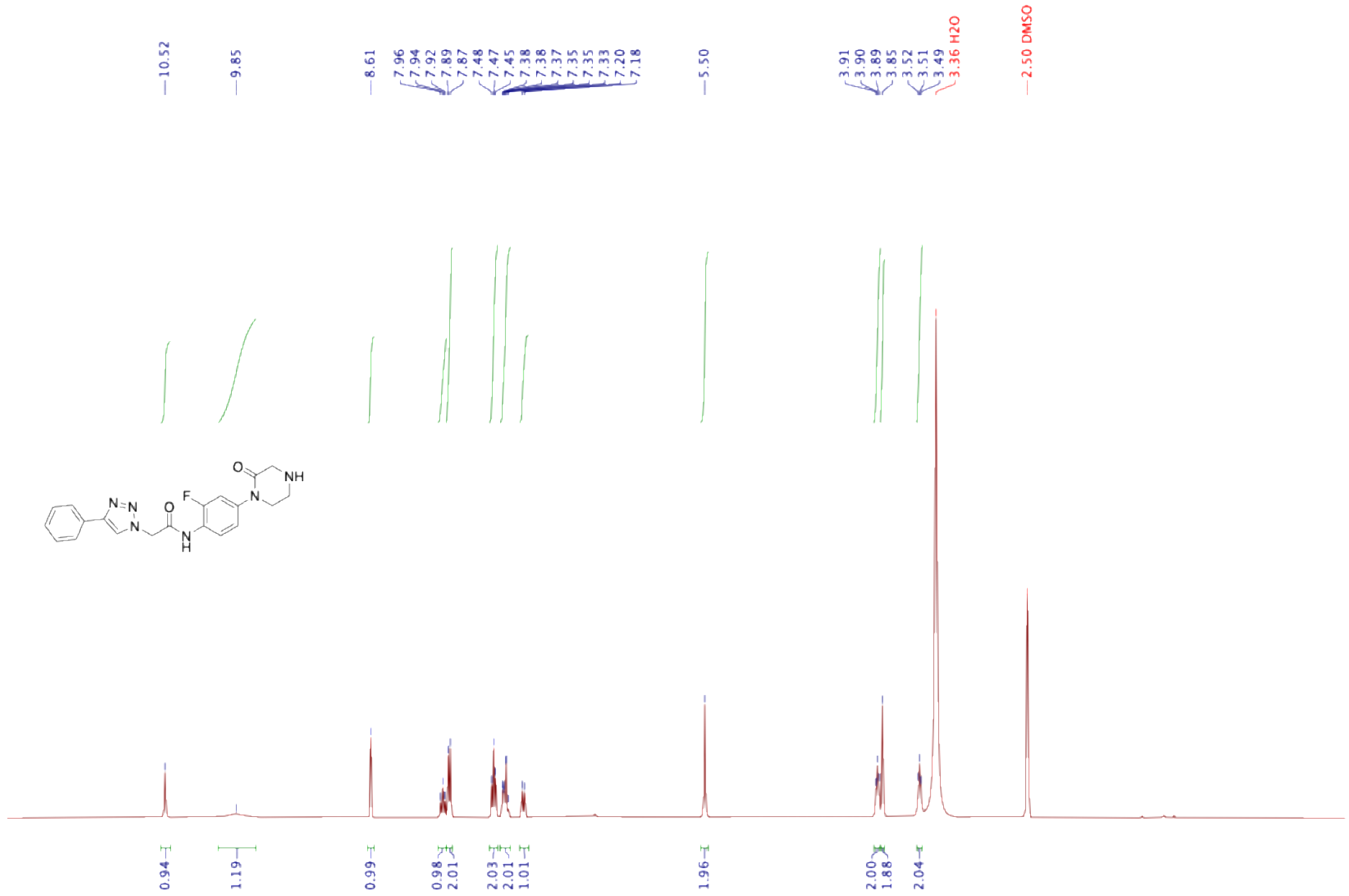

Figure 24. ^{1}H-NMR spectrum of *N*-(2-fluoro-4-(2-oxopiperazin-1-yl)phenyl)-2-(4-phenyl-1*H*-1,2,3-triazol-1-yl)acetamide (**22**) in DMSO-d_6.

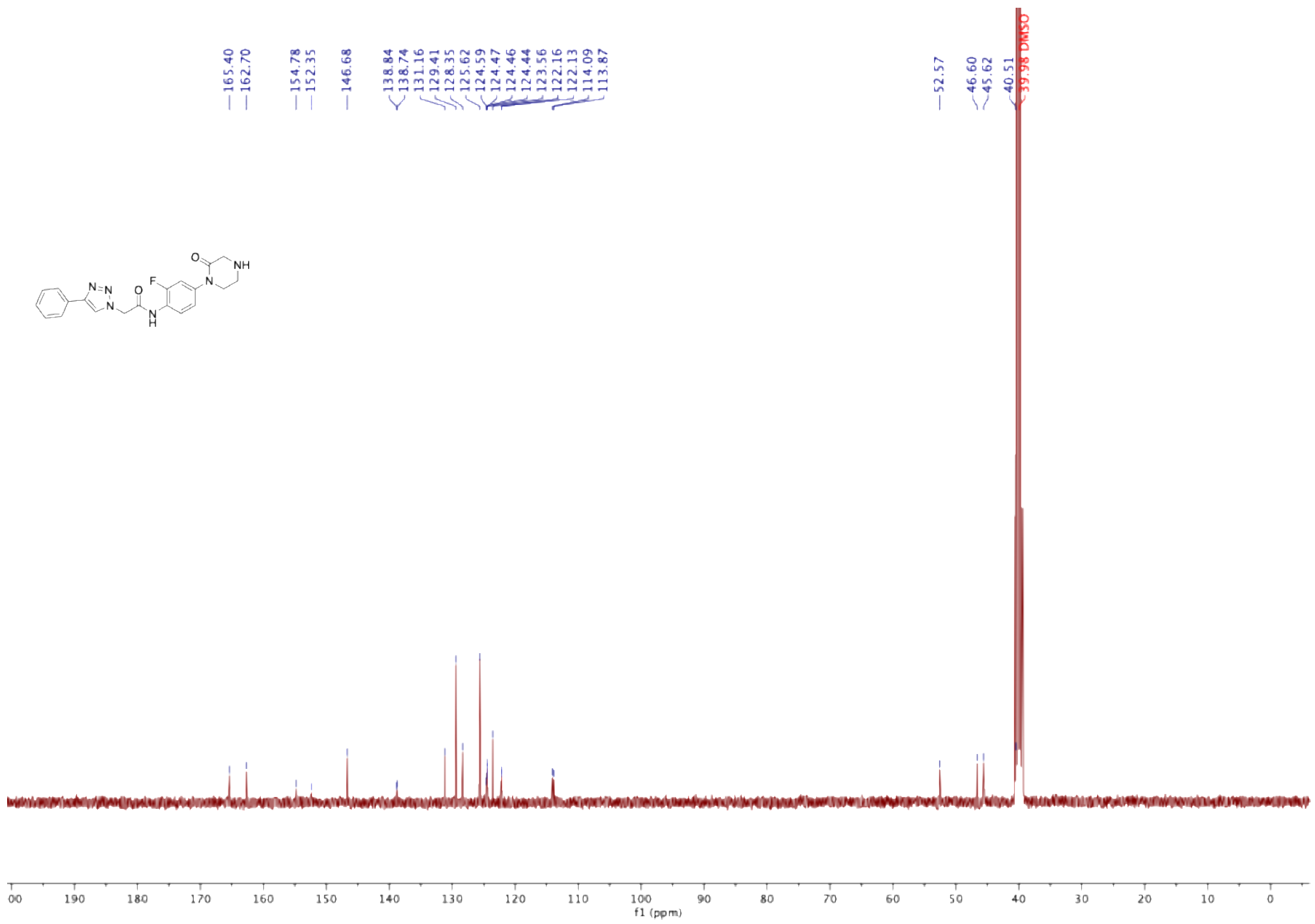

Figure 25. ^{13}C-NMR spectrum of *N*-(2-fluoro-4-(2-oxopiperazin-1-yl)phenyl)-2-(4-phenyl-1*H*-1,2,3-triazol-1-yl)acetamide (**22**) in DMSO-d_6.

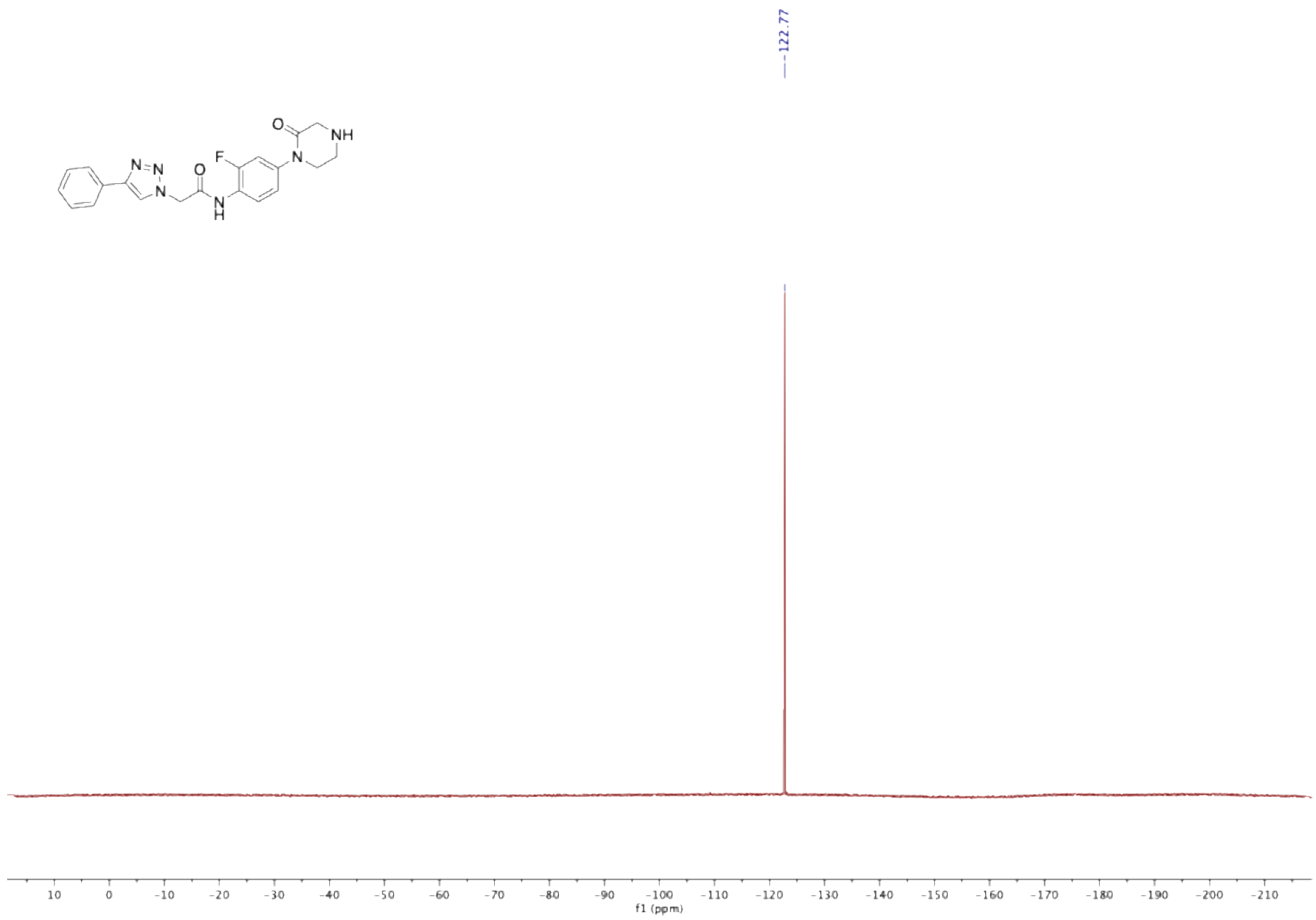

Figure 26. ^{19}F-NMR spectrum of *N*-(2-fluoro-4-(2-oxopiperazin-1-yl)phenyl)-2-(4-phenyl-1*H*-1,2,3-triazol-1-yl)acetamide (**22**) in DMSO-d_6.

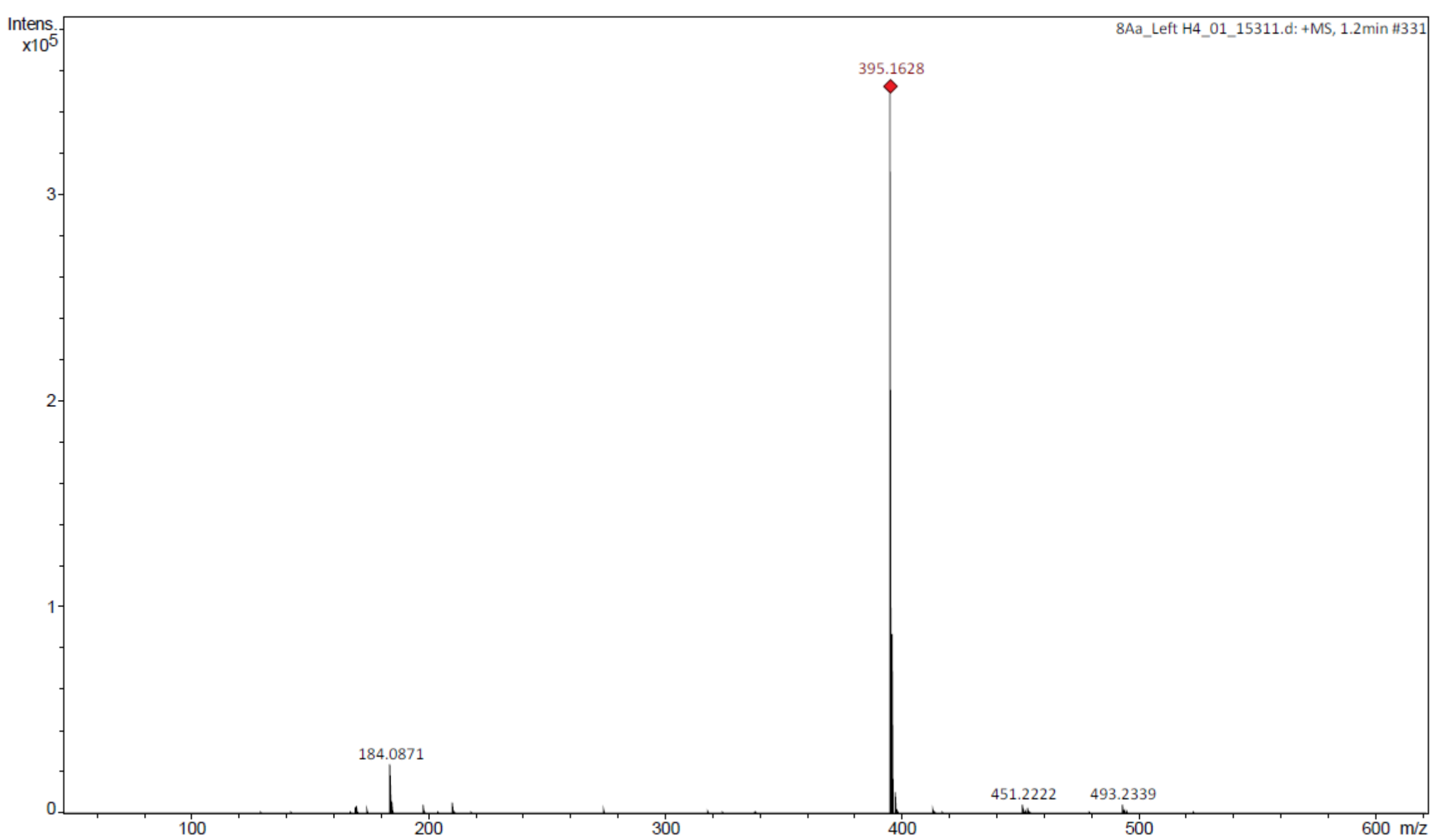

Figure 27. HRMS ESI Spectrum compound **22**.

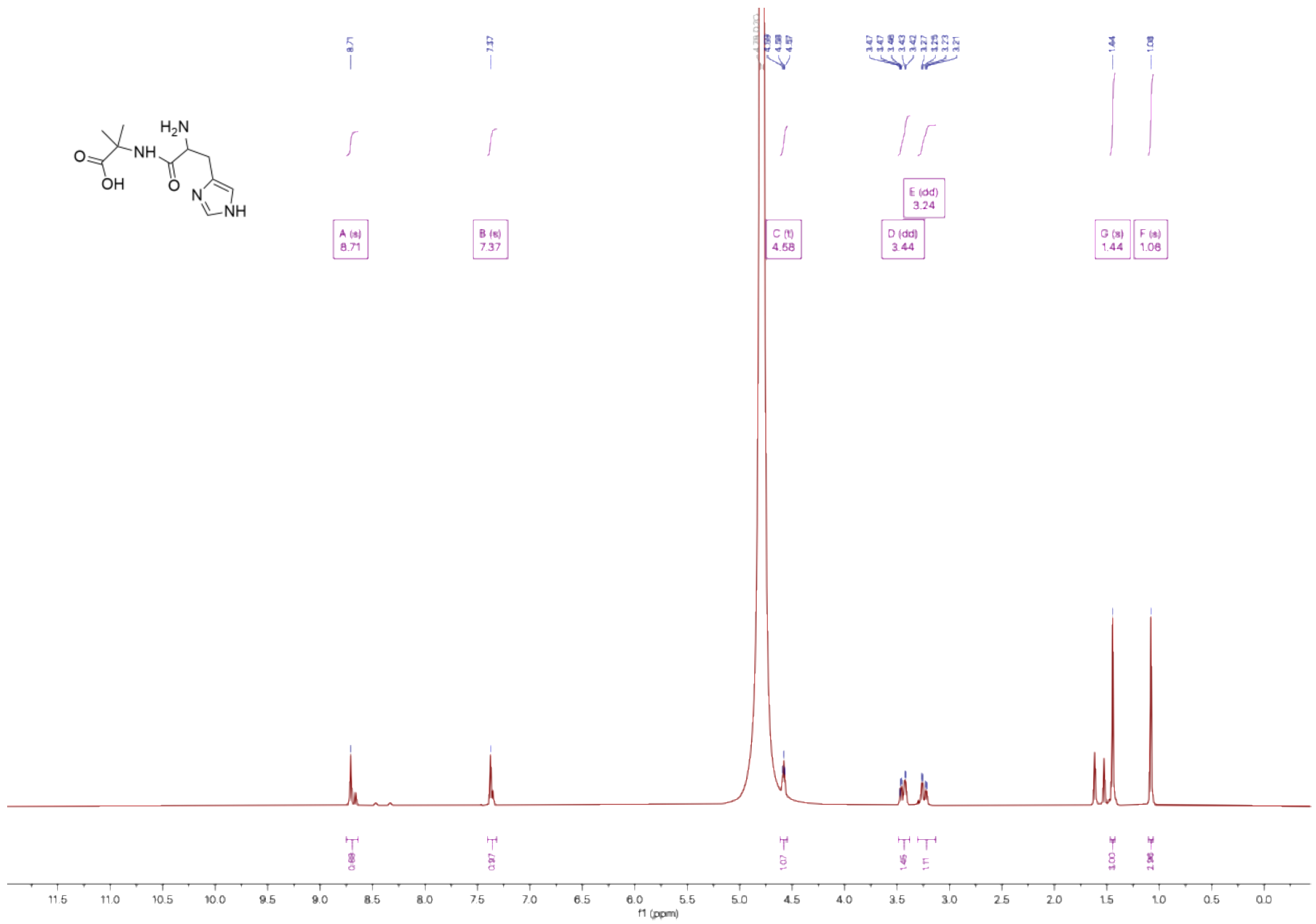

Figure 28. ^{1}H-NMR spectrum of 2-(2-Amino-3-(1*H*-imidazol-4-yl)propanamido)-2-methylpropanoic acid (**23**) in DMSO-d_6.

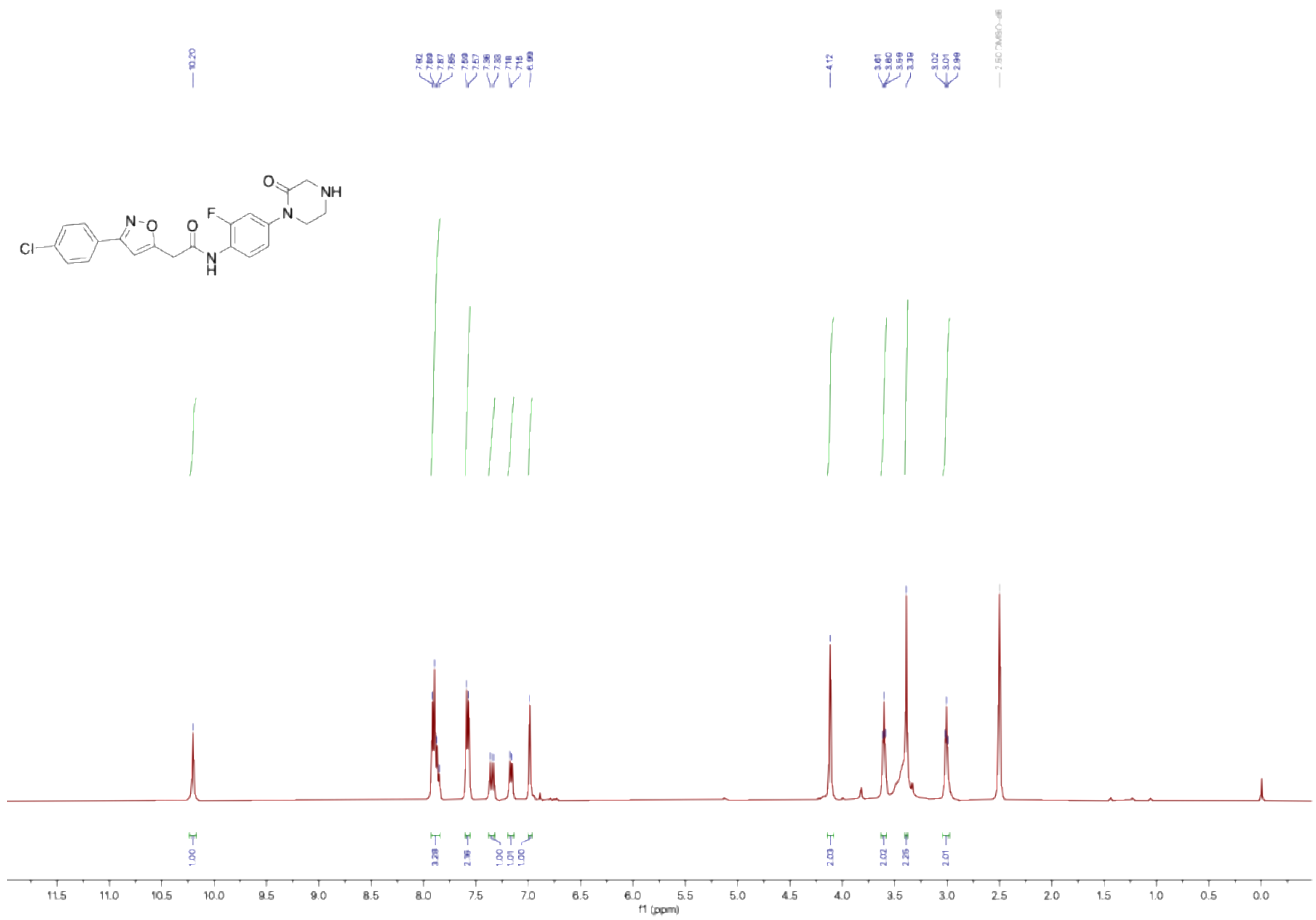

Figure 29. ^{1}H-NMR spectrum of 2-(3-(4-chlorophenyl)isoxazol-5-yl)-*N*-(2-fluoro-4-(2-oxopiperazin-1-yl)phenyl)acetamide (**24**) in DMSO-d_6.

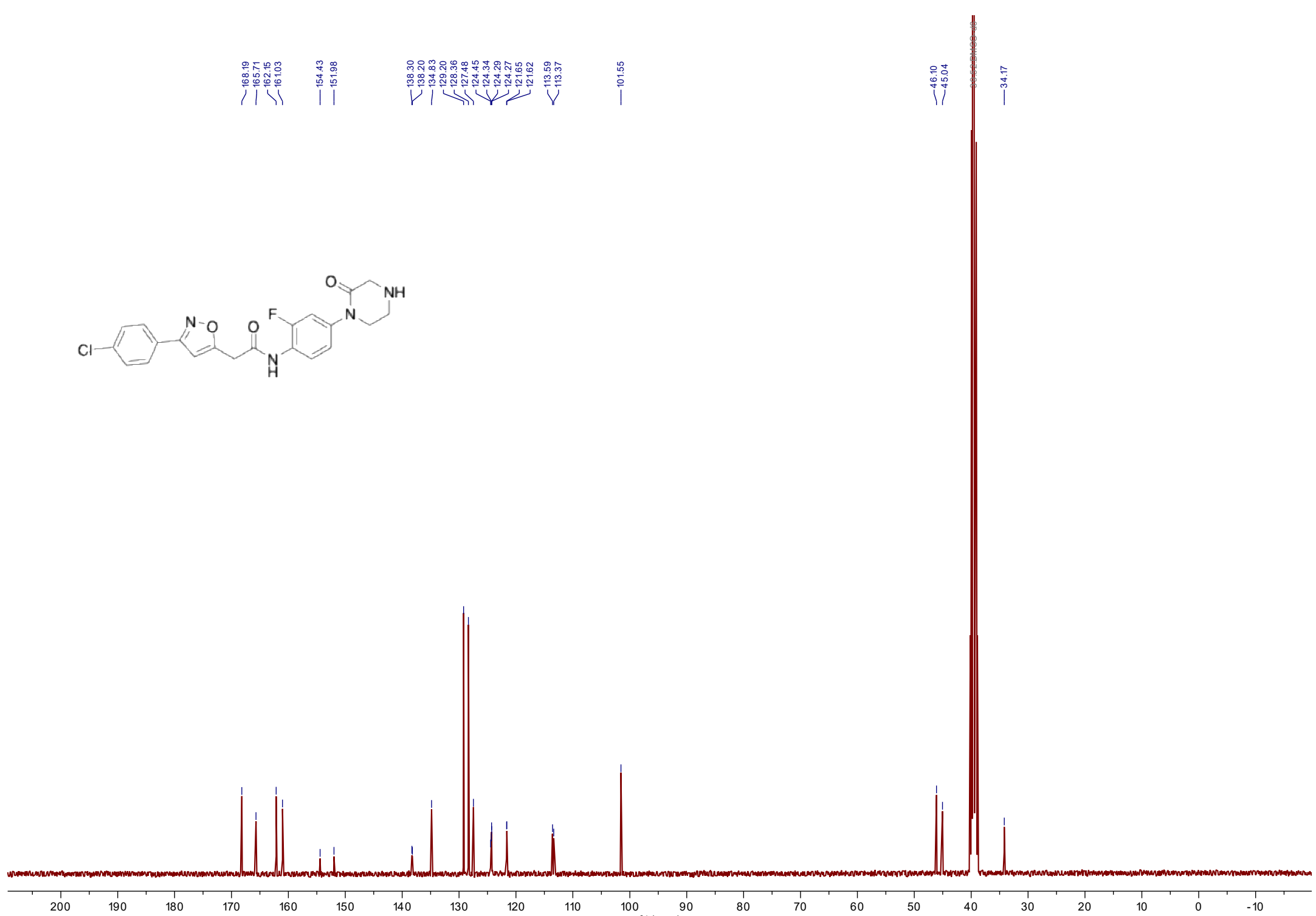

Figure 30. ^{13}C-NMR spectrum of 2-(3-(4-chlorophenyl)isoxazol-5-yl)-N-(2-fluoro-4-(2-oxopiperazin-1-yl)phenyl)acetamide (24) in DMSO-d_6.

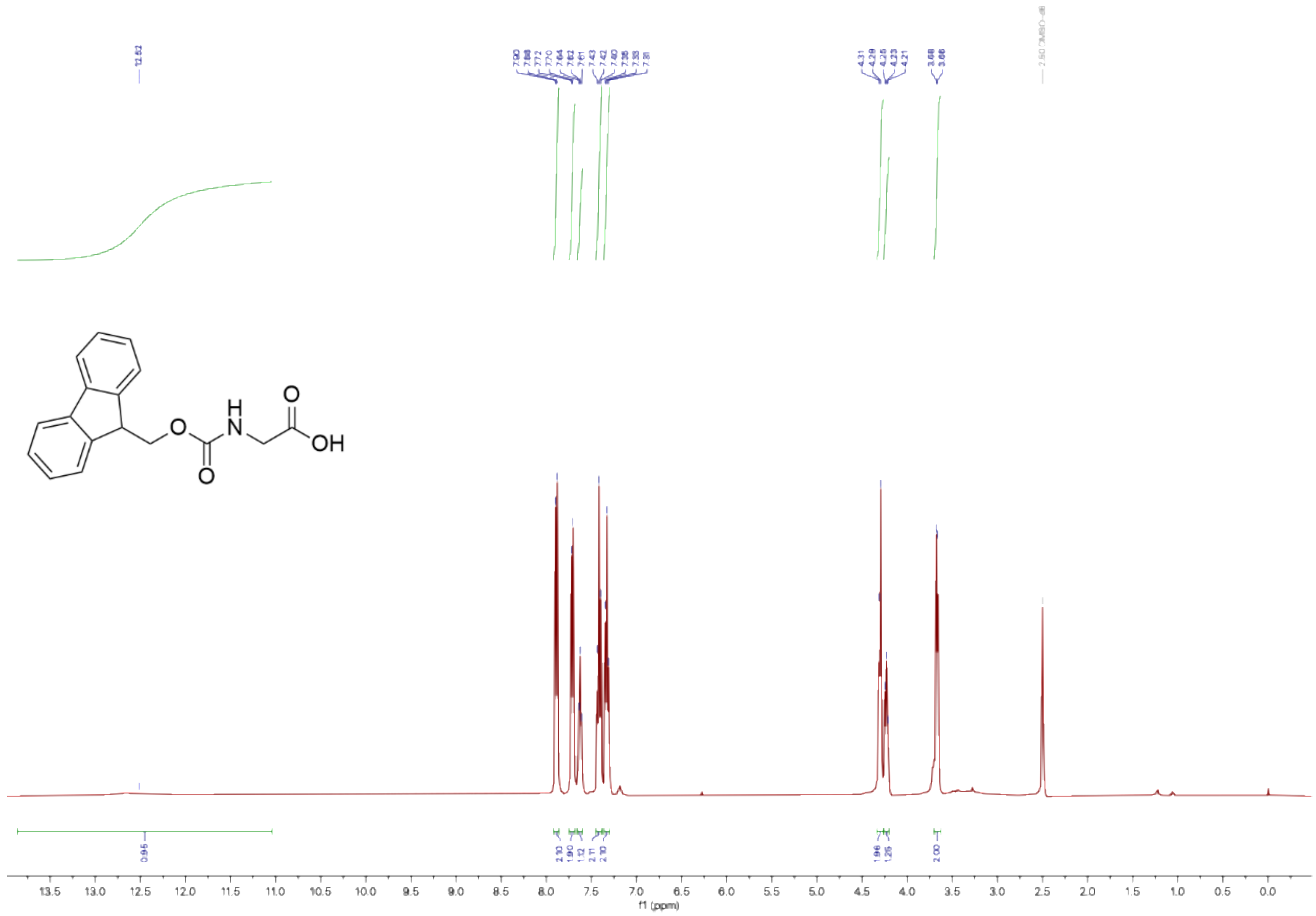

Figure 31. ^{1}H-NMR spectrum of *N*-((9-fluorenyl)methoxycarbonyl)glycine (**25**) in DMSO-d_6

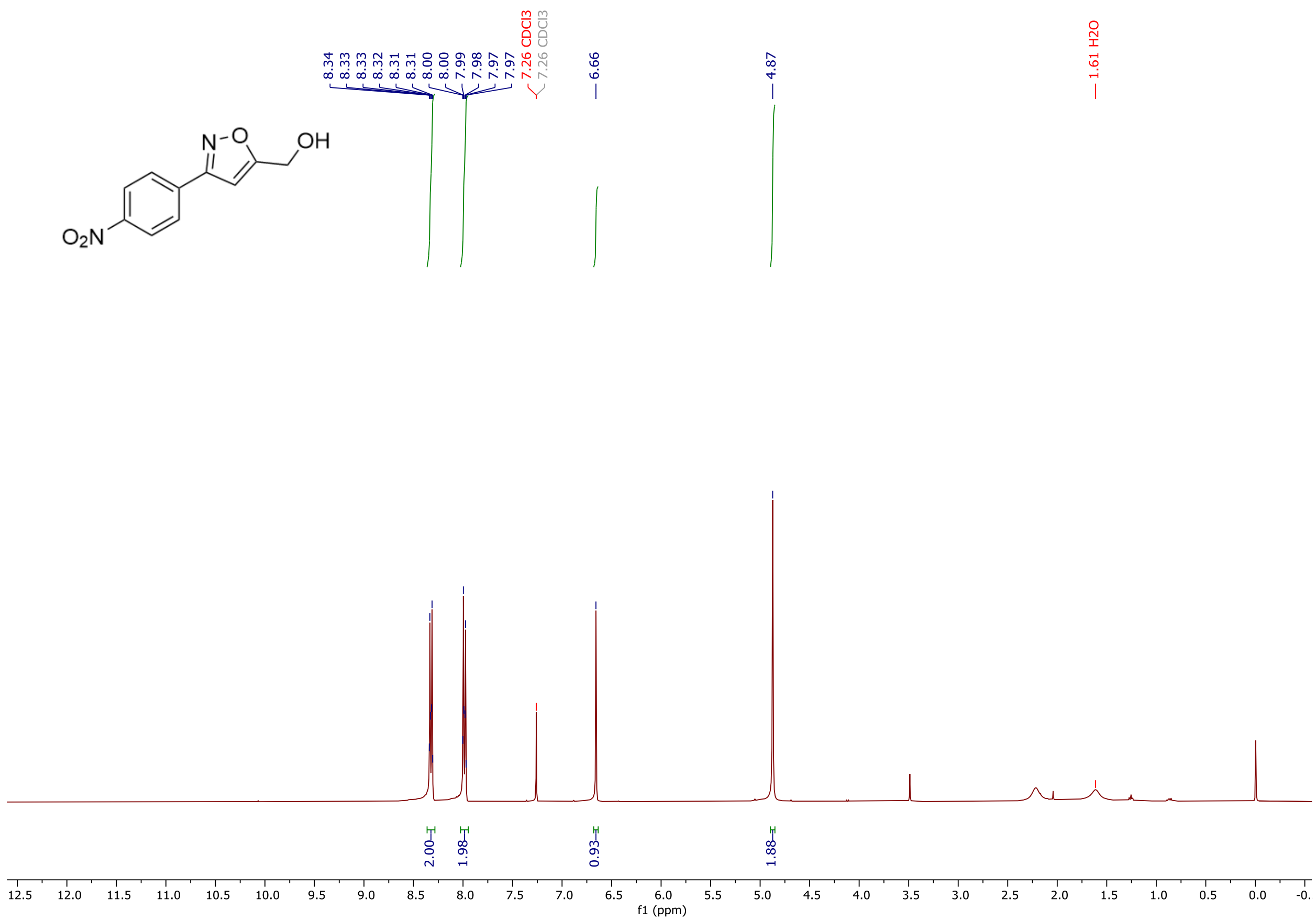

Figure 32. ^{1}H-NMR spectrum of [3-(4-nitrophenyl)isoxazol-5-yl]methanol.

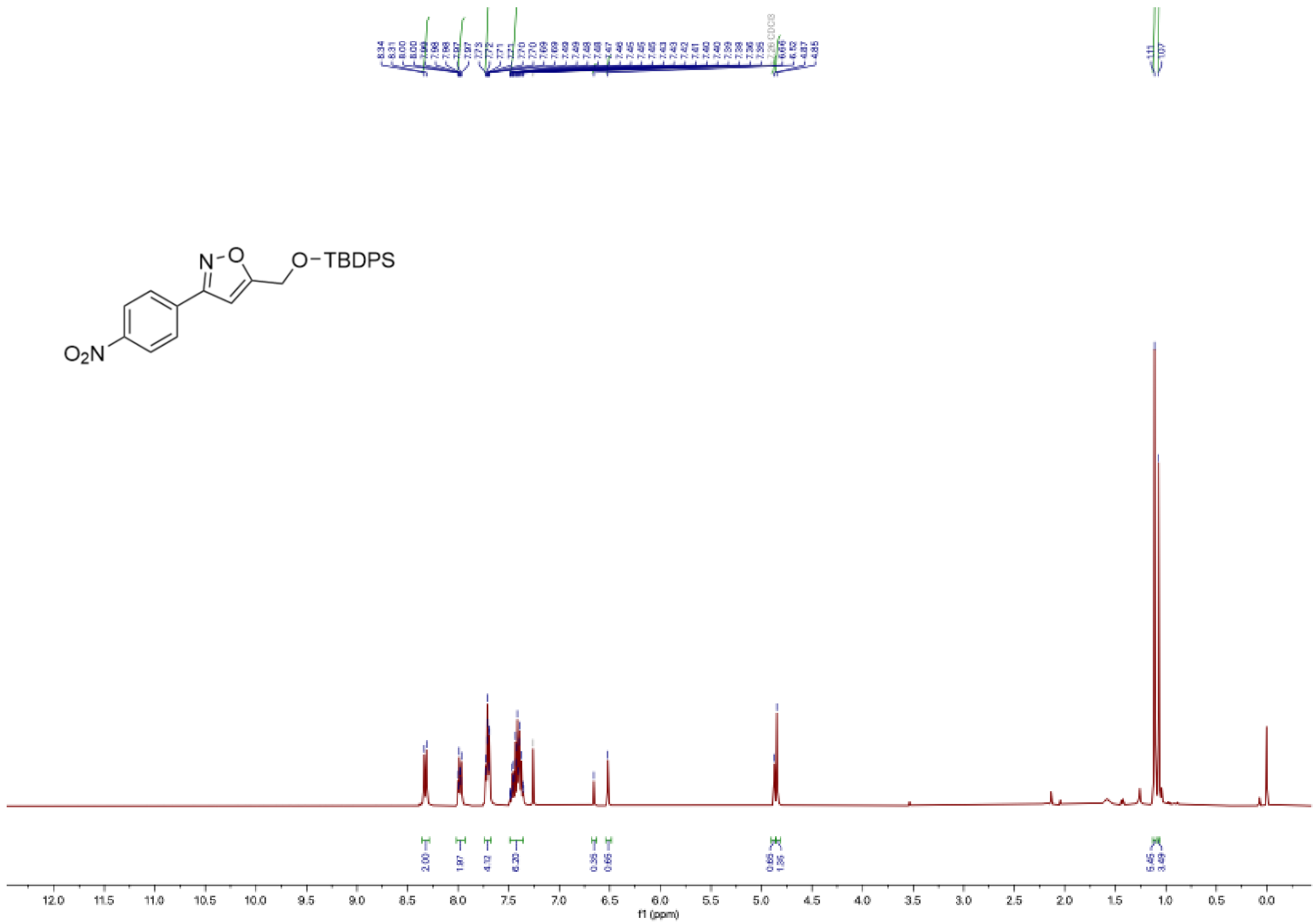

Figure 33. ^{1}H NMR spectrum of 5-(((tert-butyldiphenylsilyl)oxy)methyl)-3-(4-nitrophenyl)isoxazole

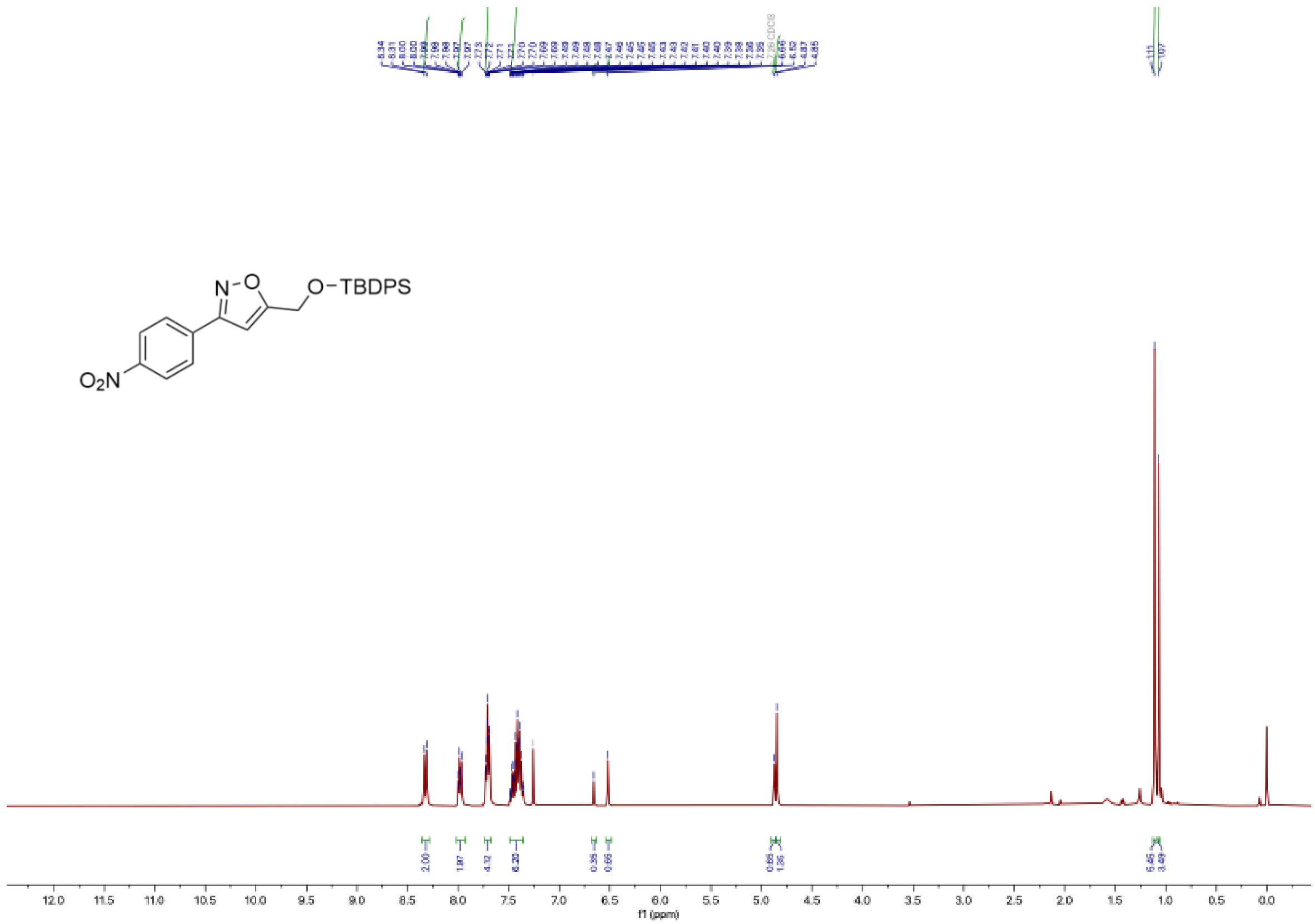

Figure 34. ^{1}H NMR spectrum (400 MHz, $CDCl_3$) of 5-(((tert-butyldiphenylsilyl)oxy)methyl)-3-(4-nitrophenyl)isoxazole after treatment with water under microwave irradiation (140 °C, 2 min).

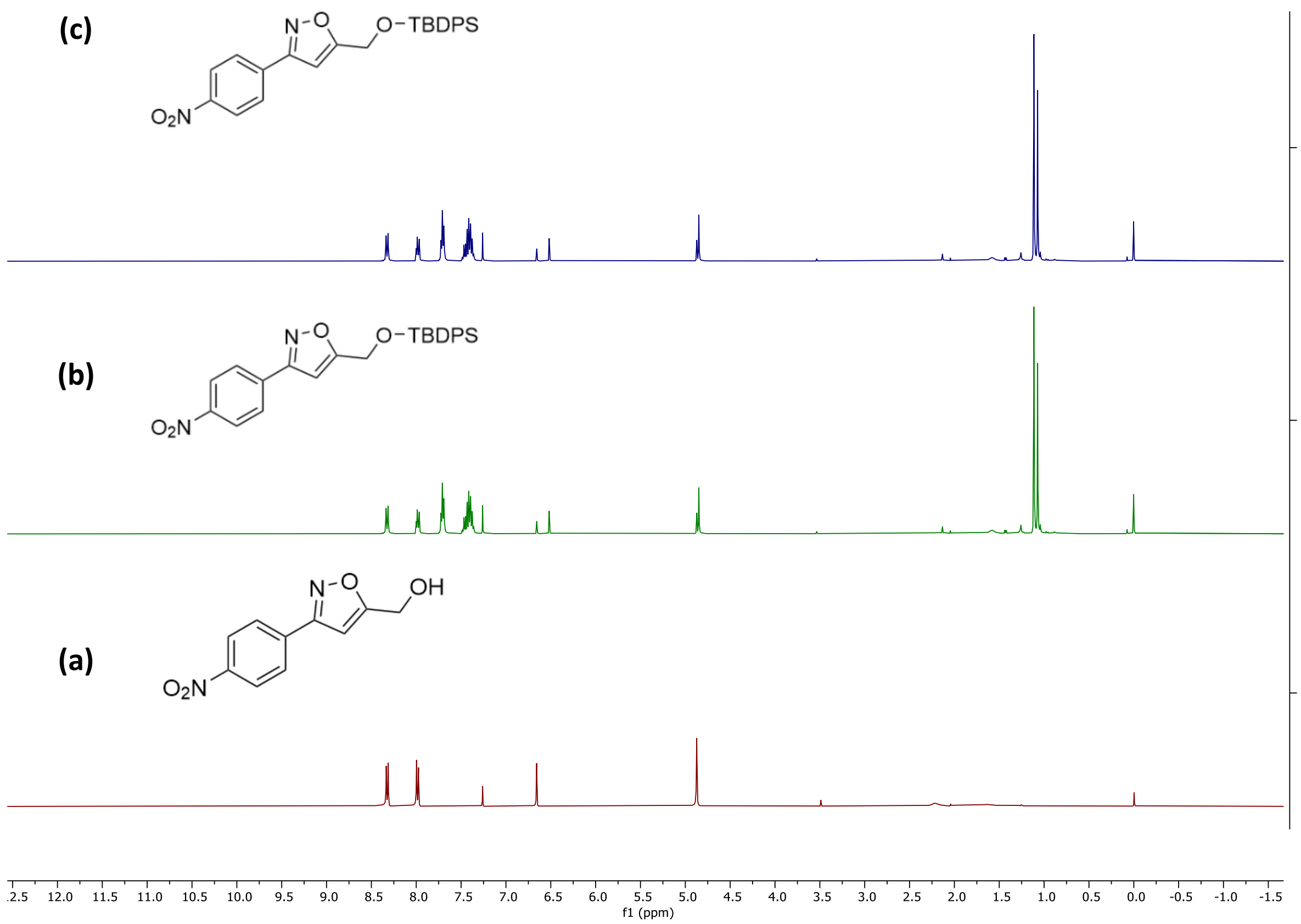

Figure 35. Stacked ^{1}H NMR spectra (400 MHz, $CDCl_3$) of **(a)** [3-(4-nitrophenyl)isoxazol-5-yl]methanol (I), **(b)** the corresponding TBDPS-protected derivative (II), and **(c)** compound II after treatment under the optimized microwave-assisted *N*-Boc deprotection conditions (H_2O, 140 °C, 2 min). The isoxazole CH, CH_2, and tert-butyl resonances appear as two sets of signals in an approximate 65:35 ratio, suggesting hindered rotation of the silyl ether and the presence of rotamers.[3]

Computational details

All geometry optimizations were conducted using density functional theory (DFT) with the B3LYP exchange–correlation functional and the def2-TZP basis set, as implemented in the ORCA 6.0 software package.[4,5] To improve the accuracy of the results, Grimme's D4 empirical dispersion correction was included, accounting for long-range dispersion interactions and providing corrections to the total energy, energy gradients, and vibrational frequencies.

Frequency calculations were performed at the same level of theory to confirm that all optimized structures correspond to true minimum on the potential energy surface, as indicated by the absence of imaginary frequencies. The diagonalization of the Hessian matrix yielded the vibrational eigenfrequencies and corresponding normal modes, which describe the harmonic motion of the molecular nuclei. All computational protocols and input files are available in the Supporting Information.

Molecular structures were initially constructed using Avogadro software (version 1.2.0),[6] followed by a preliminary optimization with the integrated "optimize geometry" extension. Subsequent optimizations were performed using density functional theory (DFT) methods, employing the selected functional and the Def2-TZP basis set with D4 dispersion corrections.[7] Optimized geometries were confirmed as true minima by vibrational frequency analysis, ensuring the absence of imaginary frequencies.

Reaction pathways were explored using the Nudged Elastic Band (NEB) method, connecting optimized reactant and product geometries. The minimum energy pathways obtained were further refined through transition state (TS) optimizations. The identity of the transition states was confirmed via frequency analysis, with the detection of a single imaginary frequency corresponding to the reaction coordinate.

```
NAME = opt.inp
|  1> ! b3lyp CPCM(water) def2-tzVP autoaux RIJCOSX normalprint printbasis printmos D4 Opt FREQ
|  2> %maxcore 4000
|  3> %pal nprocs 8
|  4> end
|  5> %geom
|  6> MaxIter 400
|  7> end
|  8>
|  9> * xyz 0 1
| 10> C        -2.64032       0.56836       0.11447
| 11> C        -3.14874      -0.71902       0.27512
| 12> C        -1.30700       0.75427      -0.25668
| 13> C        -0.47295      -0.34890      -0.48148
| 14> C        -0.98996      -1.63954      -0.29957
| 15> C        -2.32171      -1.82233       0.07600
| 16> C         0.94714      -0.15858      -0.95830
| 17> C         2.01582      -0.45726       0.10742
| 18> N         3.16094      -1.16725      -0.49202
| 19> C         4.04791      -1.87457       0.26694
| 20> O         4.01396      -1.94067       1.48241
| 21> O         5.00822      -2.36202      -0.54539
| 22> C         6.24324      -2.88579       0.01297
| 23> C         6.95434      -1.82765       0.86818
| 24> C         6.00465      -4.18354       0.78923
| 25> C         7.12615      -3.19129      -1.20680
| 26> C         2.48693       0.88873       0.69480
| 27> O         3.73857       1.24469       0.30346
| 28> O         1.78734       1.63768       1.36096
| 29> H        -3.28364       1.42992       0.27520
| 30> H        -4.18854      -0.86175       0.55735
| 31> H        -0.92962       1.76850      -0.37421
| 32> H        -0.36350      -2.51370      -0.46131
| 33> H        -2.71670      -2.82641       0.20661
| 34> H         1.06617       0.86049      -1.35127
| 35> H         1.07576      -0.81367      -1.83035
| 36> H         1.64177      -1.06972       0.93600
| 37> H         3.21422      -1.32850      -1.49085
| 38> H         6.42015      -1.62830       1.80207
| 39> H         7.01634      -0.87362       0.33201
| 40> H         7.96800      -2.14418       1.13556
| 41> H         5.36903      -4.02667       1.66547
| 42> H         6.94937      -4.61536       1.13782
| 43> H         5.49626      -4.92435       0.16214
| 44> H         7.29000      -2.28639      -1.80405
| 45> H         6.63968      -3.91520      -1.87060
| 46> H         8.10195      -3.59184      -0.91247
| 47> H         4.14273       0.52780      -0.22423
| 48> *
| 49>
| 50>
| 51>                          ****END OF INPUT****
================================================================================
```

Figure 36. Input example optimization reagent and product

```
================================================================================
                                       INPUT FILE
================================================================================
NAME = neb.inp
|  1> ! b3lyp CPCM(water) def2-tzVP autoaux RIJCOSX normalprint printbasis printmos D4 NEB-TS FREQ
|  2> %maxcore 4000
|  3> %pal nprocs 8
|  4> end
|  5>
|  6> %NEB NEB_END_XYZFILE "prod.xyz" END
|  7>
|  8> * XYZFILE 0 1 opt.xyz
|  9>
| 10>
| 11>                          ****END OF INPUT****
================================================================================
```

Figure 37. Input file NEB TS

Table 1. Global reactivity descriptors, and HOMO LUMO orbital energies of the reactants. Global reactivity descriptors, and HOMO LUMO orbital energies of the reactants.

Entry	Hardness	Softness	Chemical potential	Electrophilicity
1	3.62	0.28	-3.76	1.95
2	3.47	0.29	-3.76	2.04
3	3.19	0.31	-3.65	2.09
4	2.81	0.36	-3.78	2.55
5	3.34	0.30	-3.23	1.56
6	3.93	0.25	-3.10	1.23
7	3.45	0.29	-3.50	1.77
8	3.26	0.31	-3.59	1.98
9	3.27	0.31	-3.50	1.87
10	3.46	0.29	-3.16	1.44
11	3.37	0.30	-3.17	1.49
12	2.99	0.33	-3.83	2.46
13	2.67	0.38	-3.08	1.78
14	2.71	0.37	-3.18	1.87
15	2.82	0.36	-2.86	1.46
16	2.88	0.35	-3.27	1.86
17	2.74	0.37	-3.39	2.10
18	2.77	0.36	-3.17	1.81
19	2.43	0.41	-3.97	3.24
20	2.58	0.39	-4.07	3.21
21	2.99	0.33	-4.37	3.19
22	2.63	0.38	-3.70	2.59
23	2.63	0.38	-3.50	2.33

Table 2. HOMO/LUMO orbital representation of the calculated reagents

	HOMO	LUMO
**1**	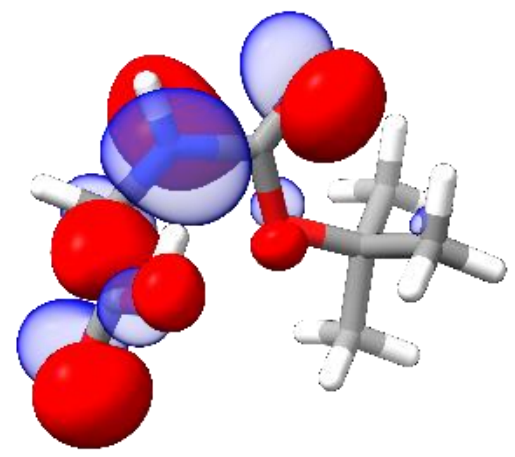	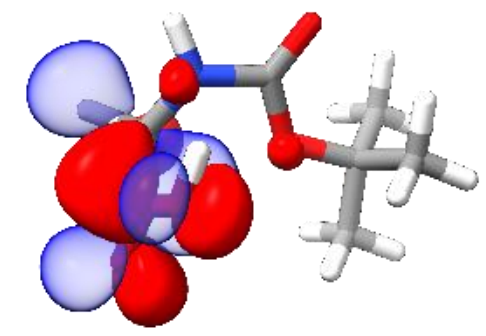
**2**	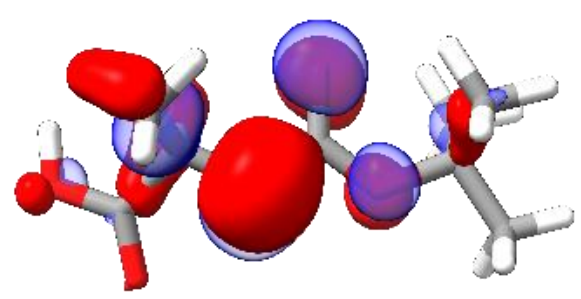	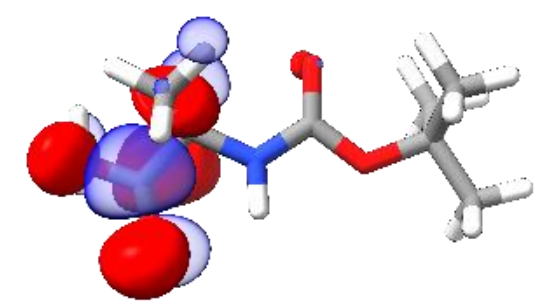
**3**	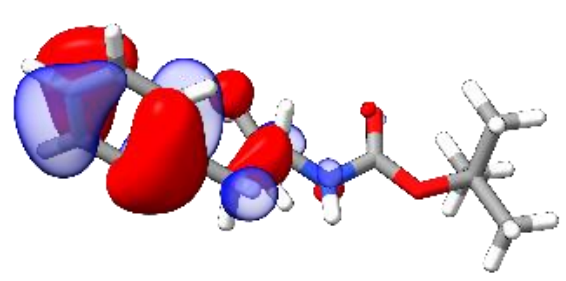	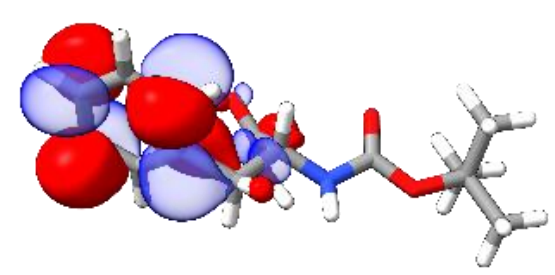
**4**	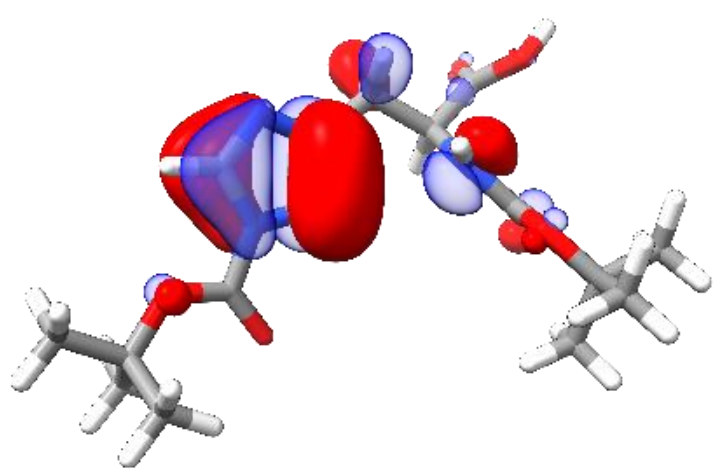	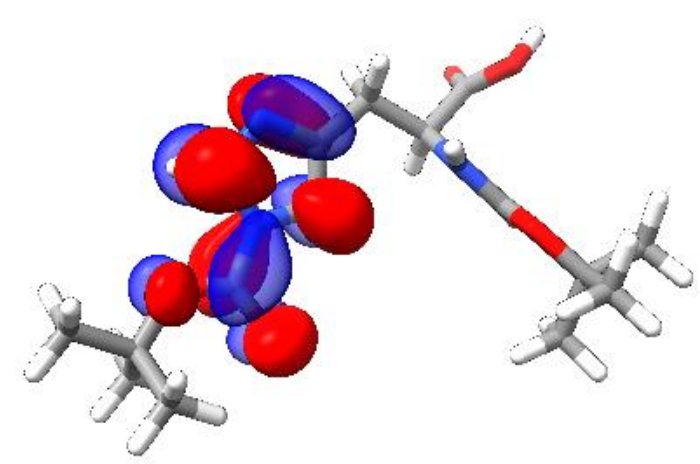
**5**	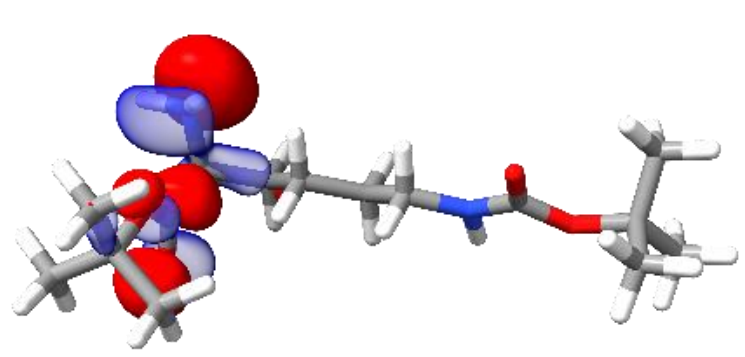	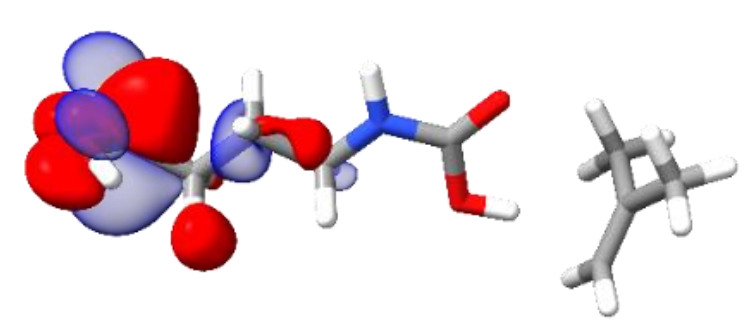

6

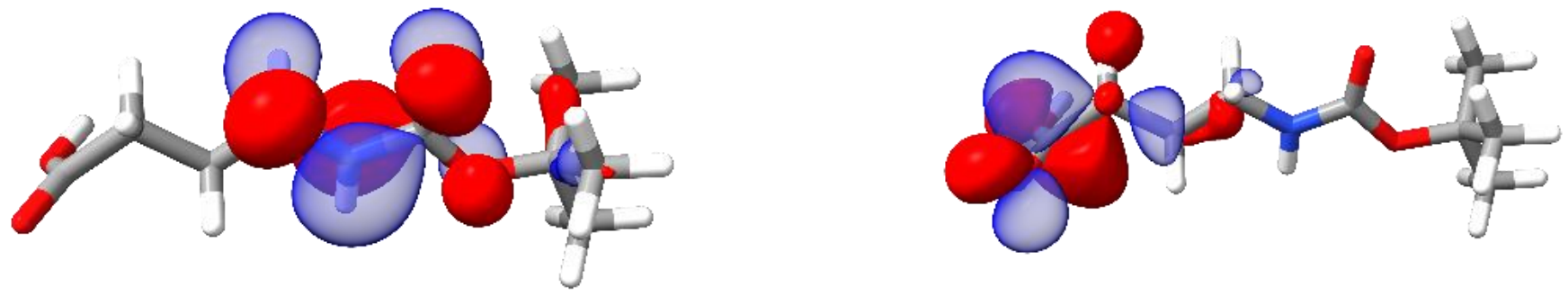

7

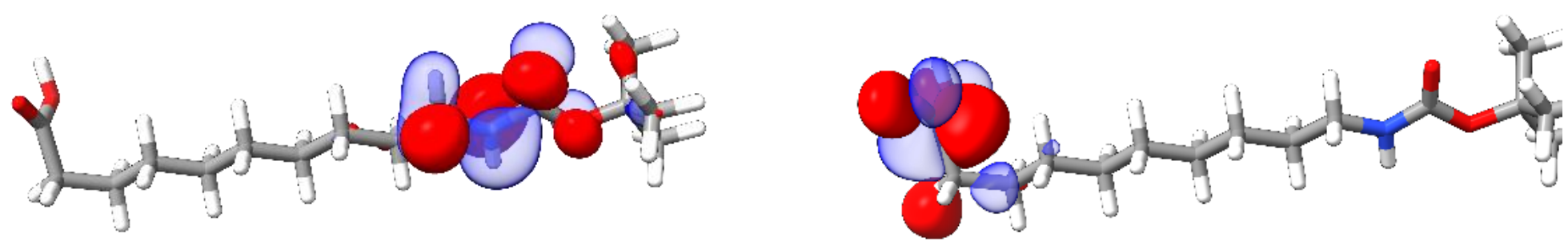

8

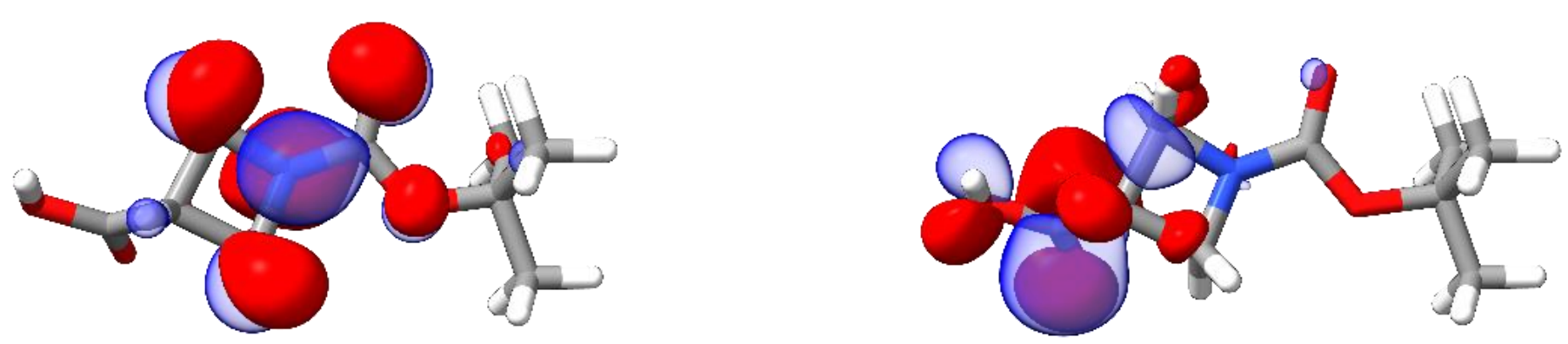

9

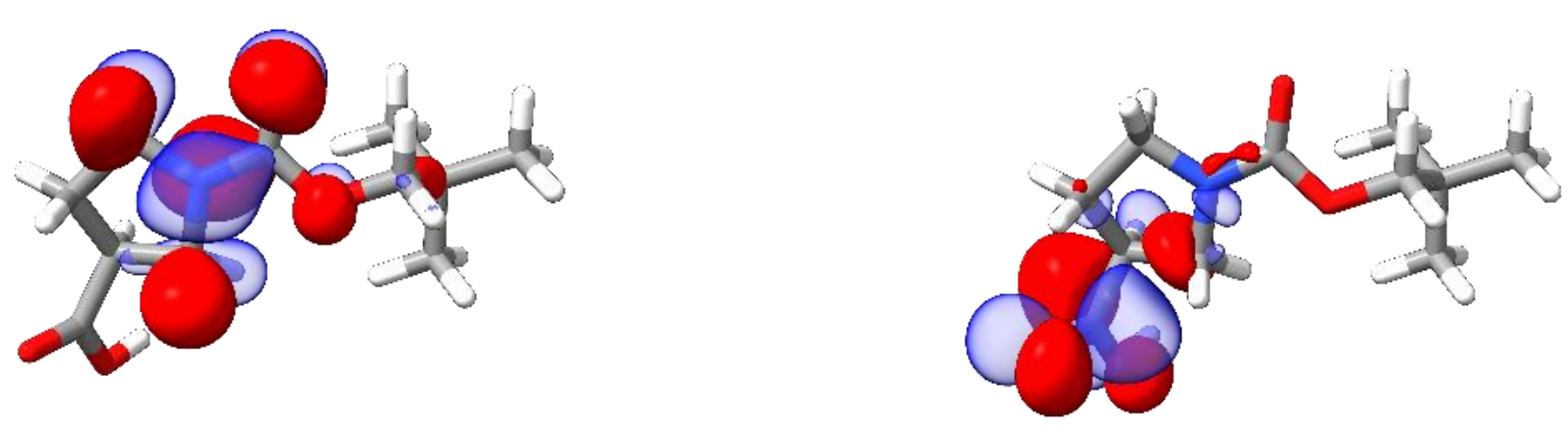

10

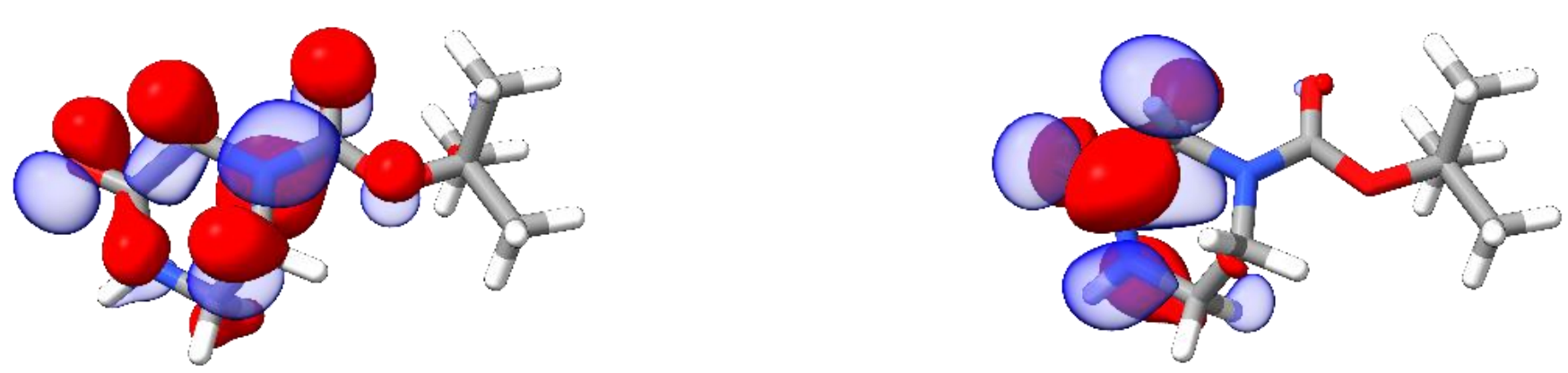

11

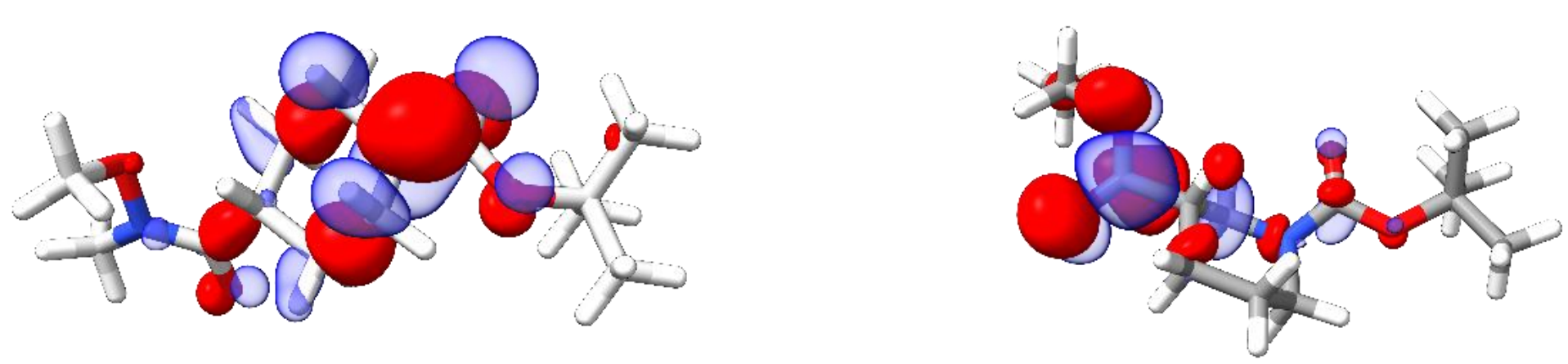

12

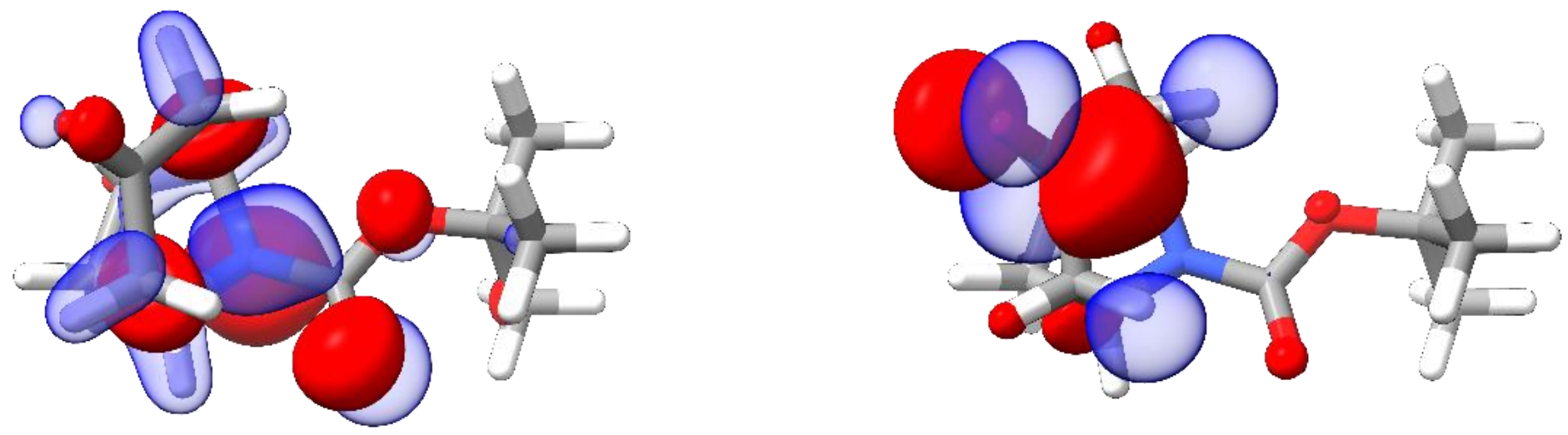

13

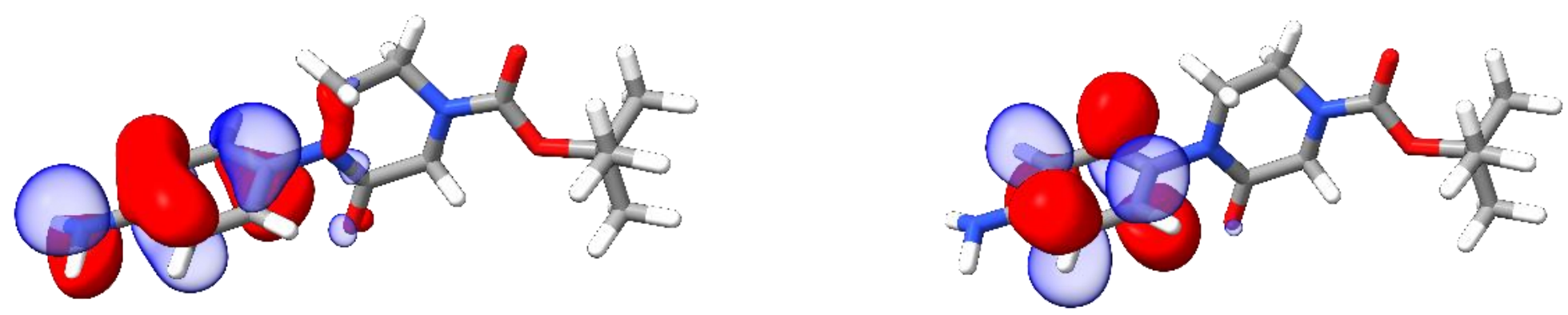

14

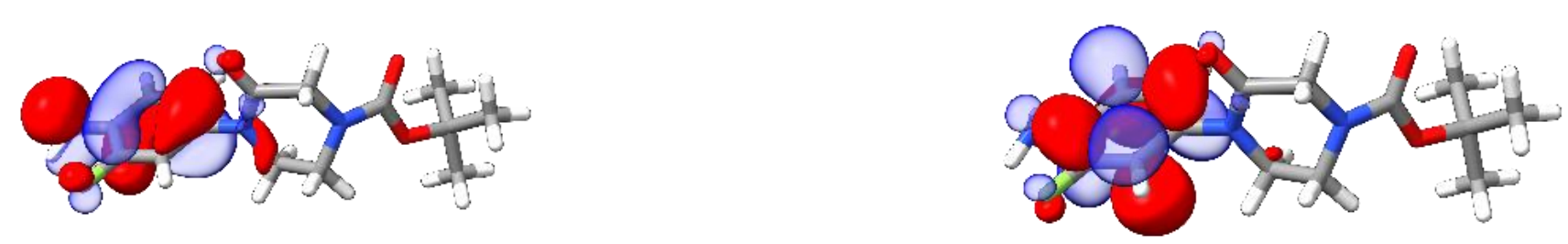

15

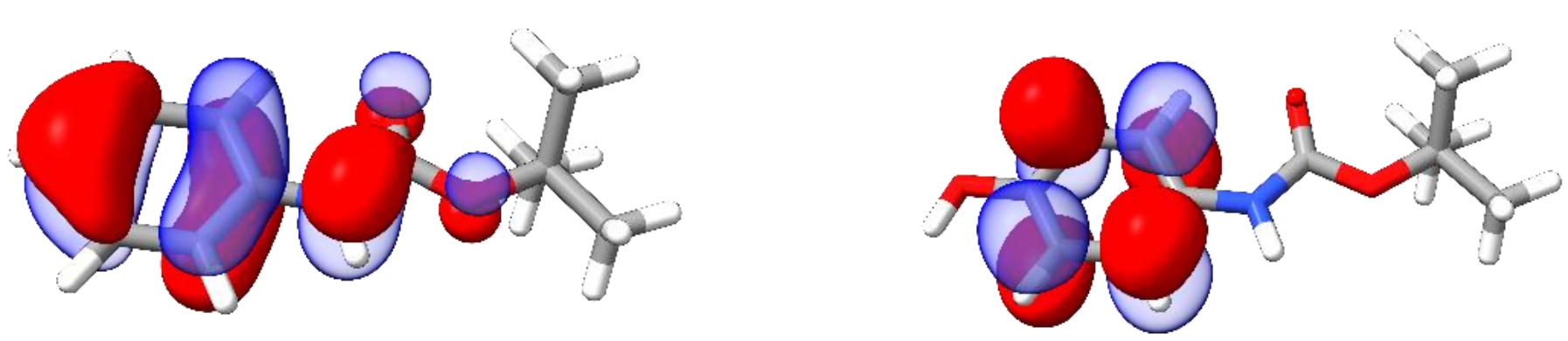

16

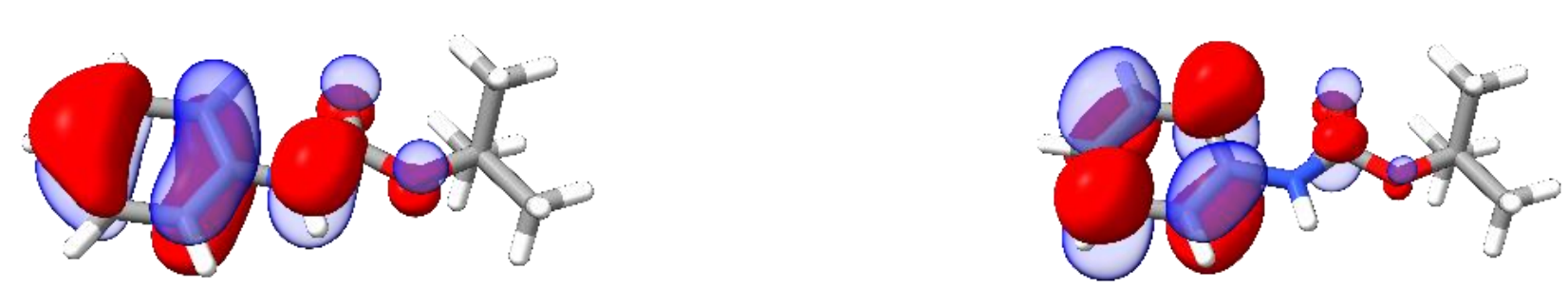

**17**	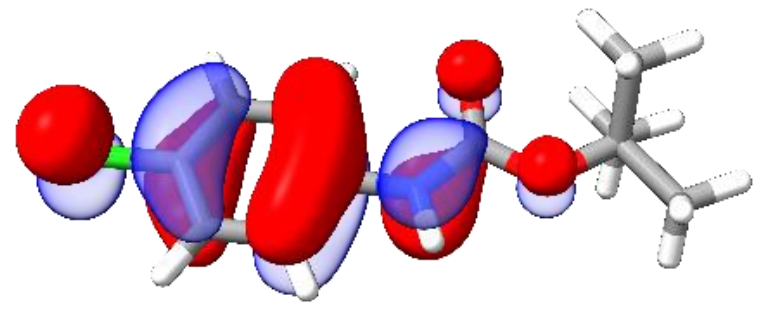	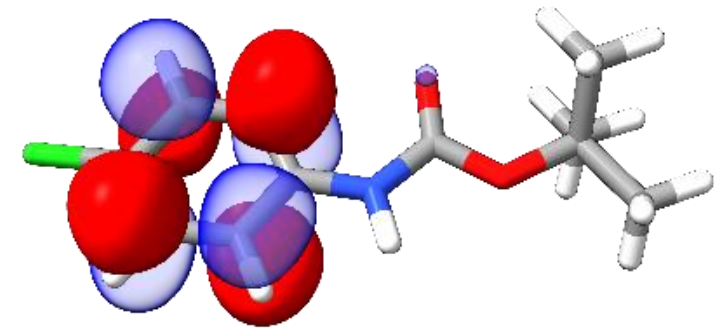
**18**	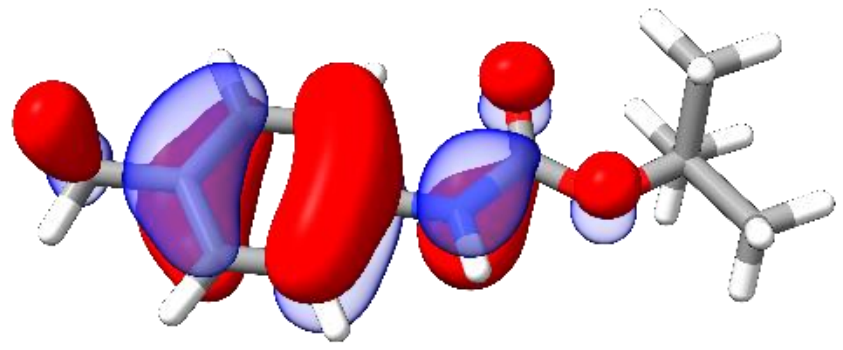	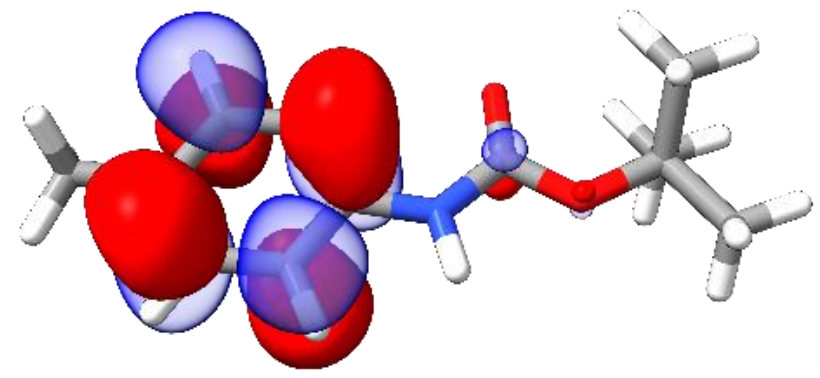
**19**	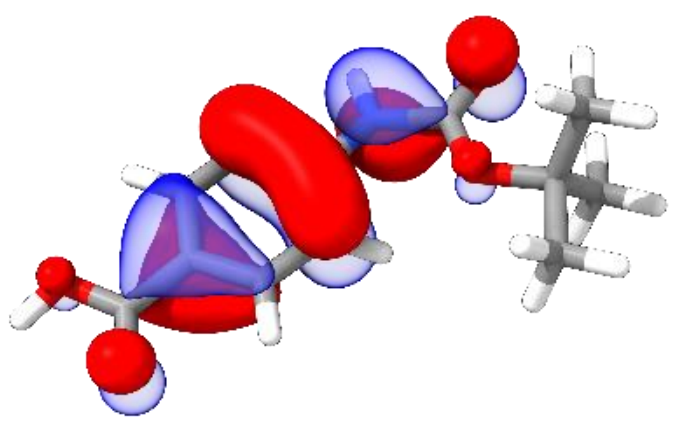	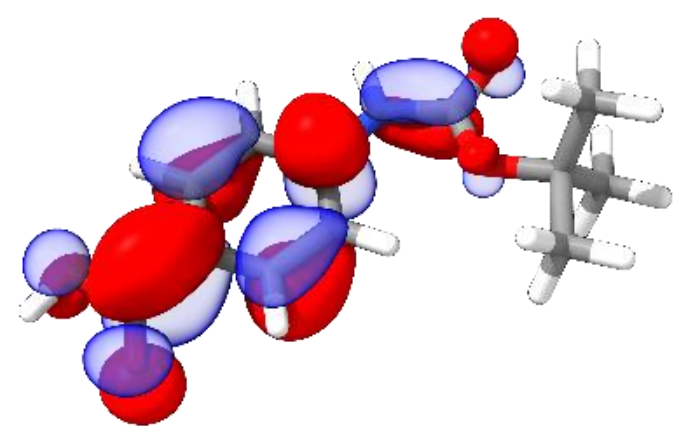
**20**	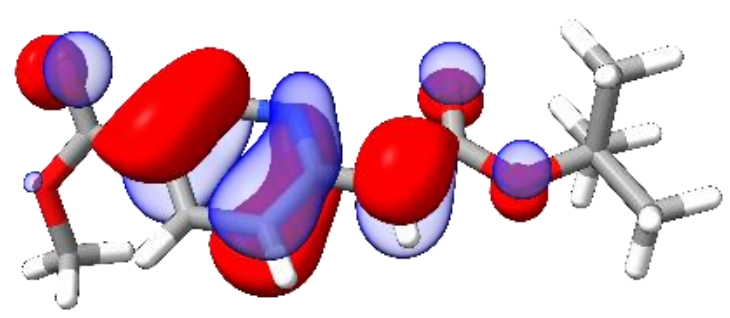	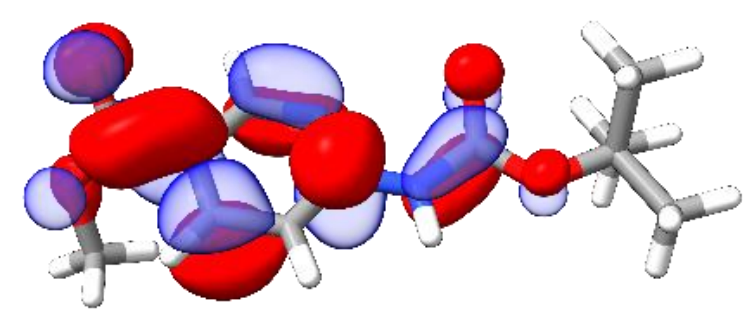
**21**	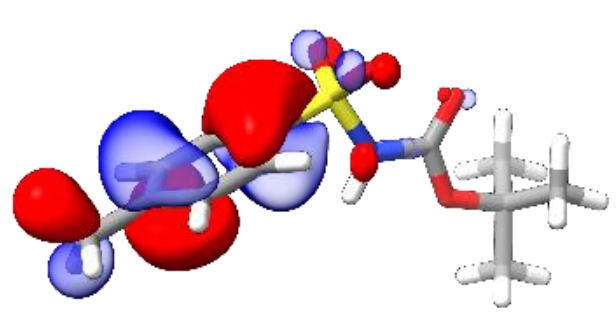	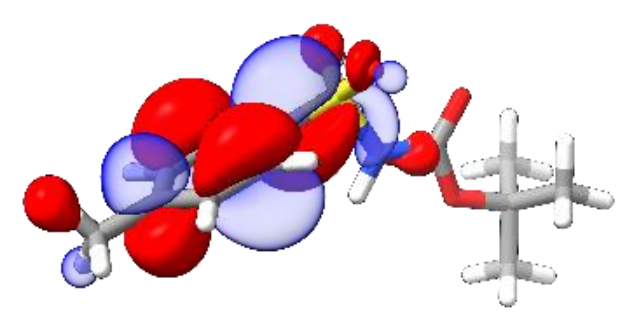
**22**	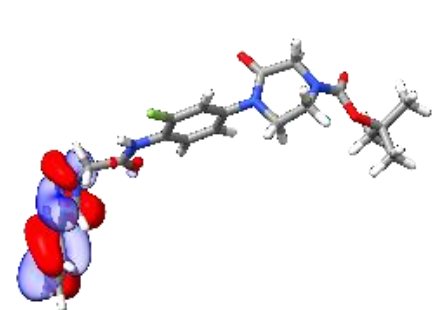	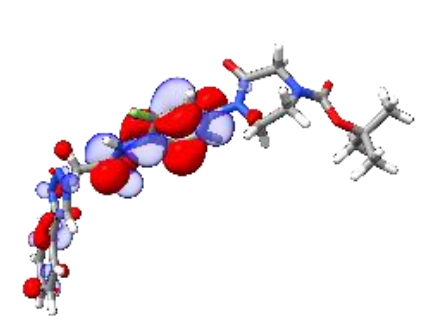

23	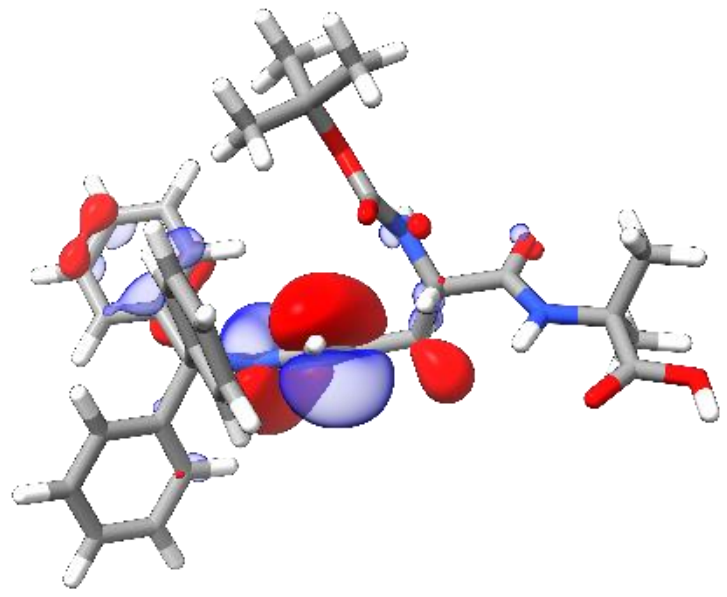	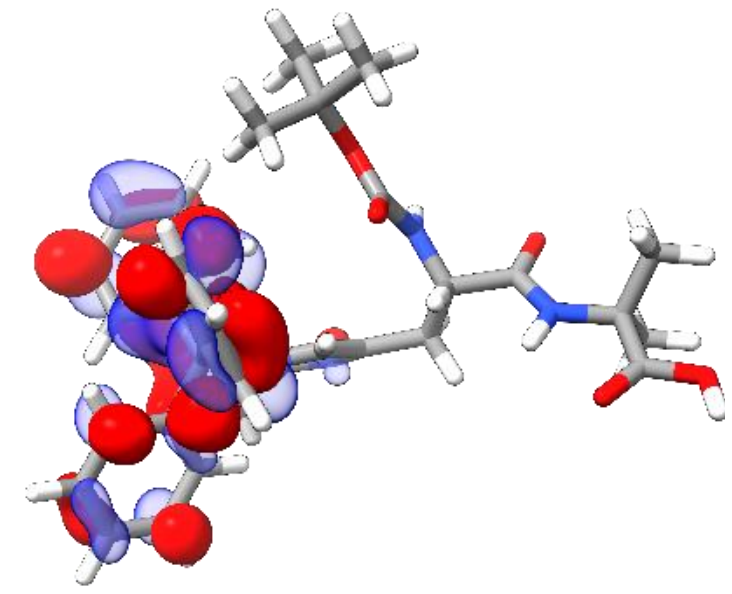

Table 3. Global reactivity descriptors, and HOMO LUMO orbital energies of the products.

Entry	Hardness	Softness	Chemical potential	Electrophilicity
1	3.45	0.29	-3.63	1.91
2	3.36	0.30	-3.68	2.01
3	3.18	0.31	-3.66	2.10
4	2.82	0.36	-3.80	2.56
5	3.34	0.30	-3.23	1.56
6	3.44	0.29	-3.58	1.86
7	3.49	0.29	-3.53	1.79
8	3.31	0.30	-3.66	2.02
9	3.31	0.30	-3.55	1.91
10	3.44	0.29	-3.33	1.62
11	3.41	0.29	-3.29	1.59
12	3.04	0.33	-3.92	2.52
13	2.67	0.38	-3.08	1.78
14	2.71	0.37	-3.19	1.88
15	2.69	0.37	-3.20	1.91
16	2.88	0.35	-3.33	1.93
17	2.75	0.36	-3.44	2.15
18	2.79	0.36	-3.21	1.85
19	2.45	0.41	-4.03	3.31
20	2.59	0.39	-4.12	3.27
21	2.85	0.35	-4.26	3.18
22	2.63	0.38	-3.71	2.61
23	2.60	0.38	-3.58	2.46

Table 4. HOMO/LUMO orbital representation of the calculated products

	HOMO	LUMO
**1**	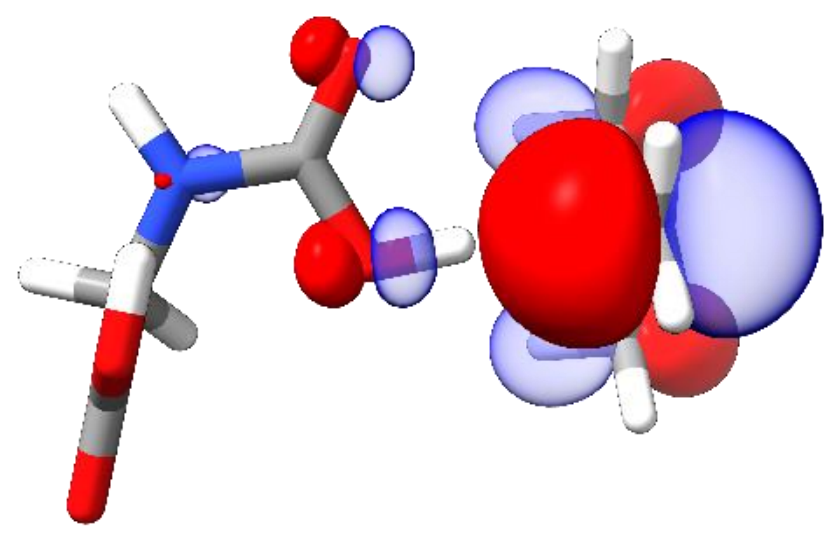	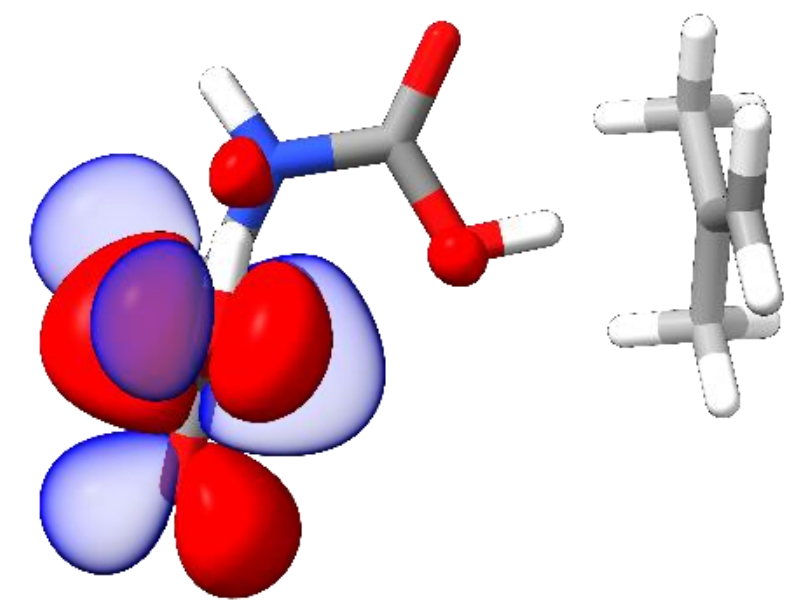
**2**	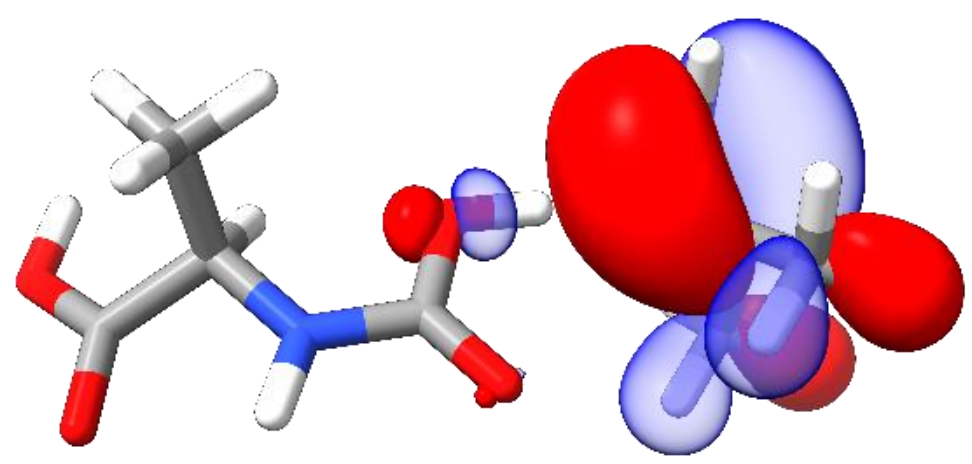	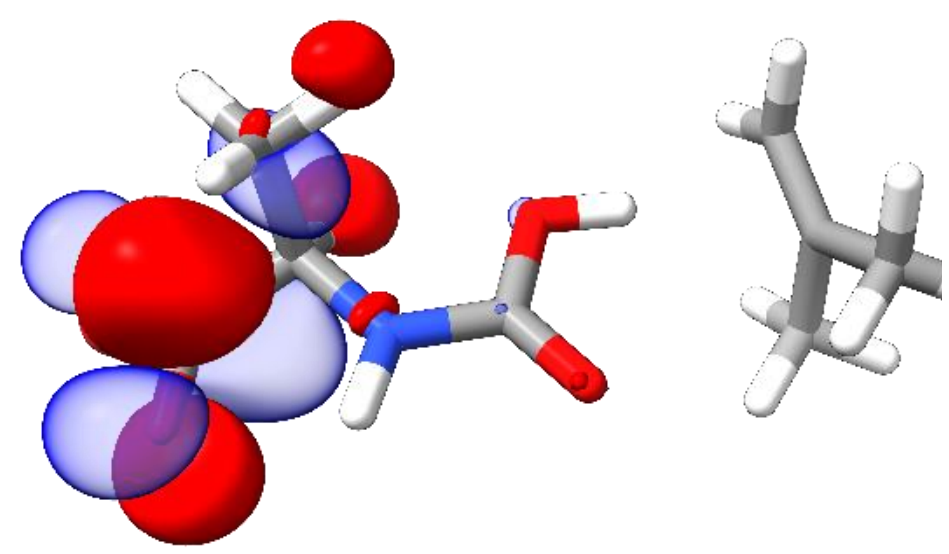
**3**	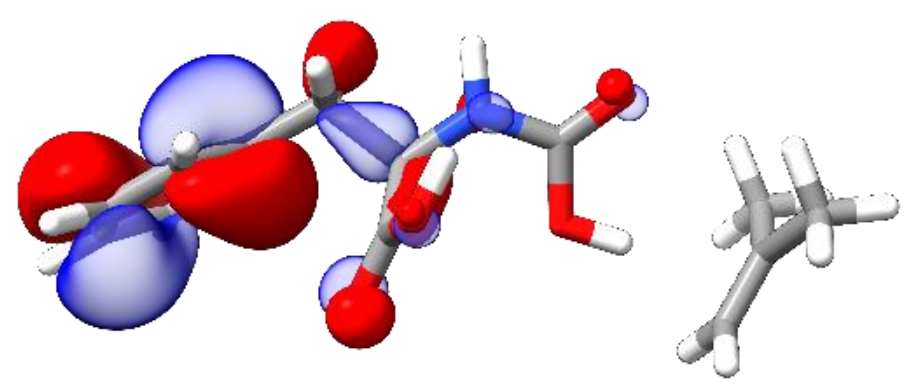	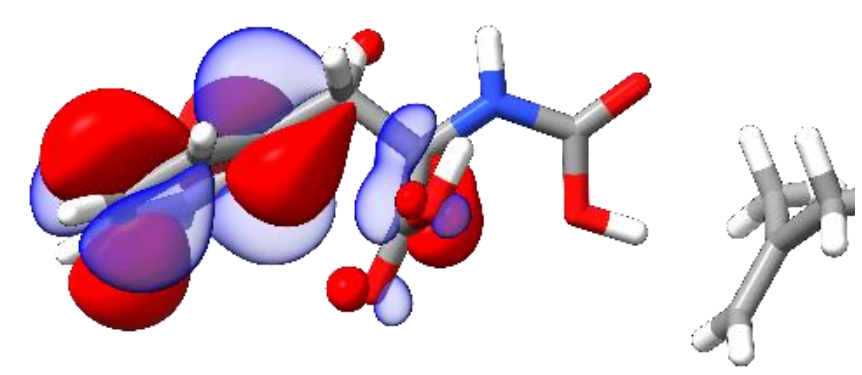
**4**	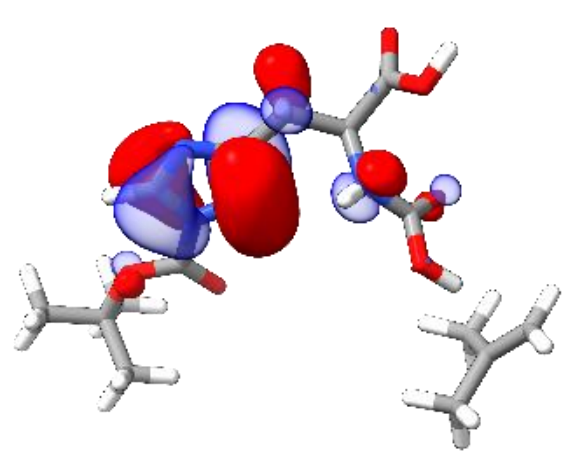	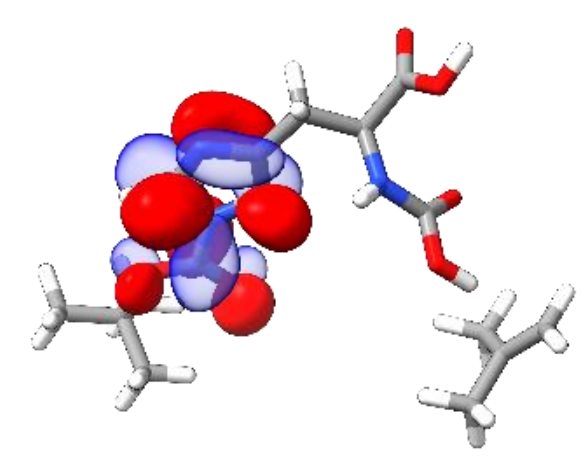
**5**	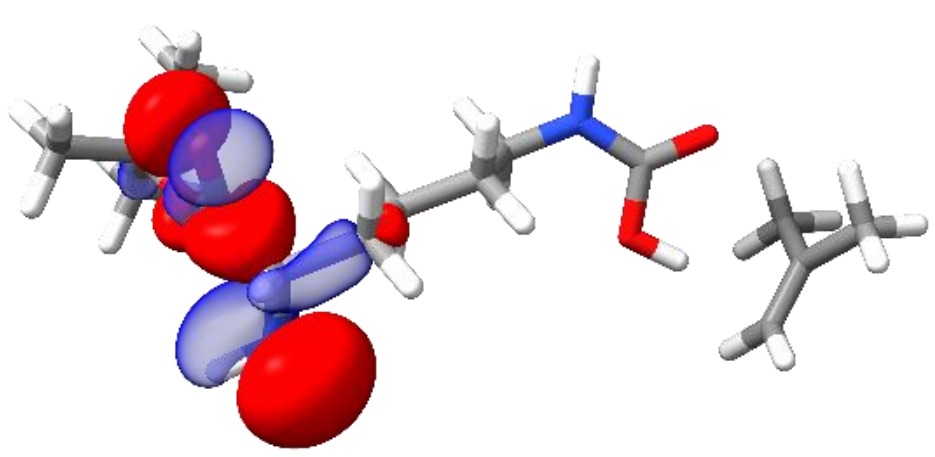	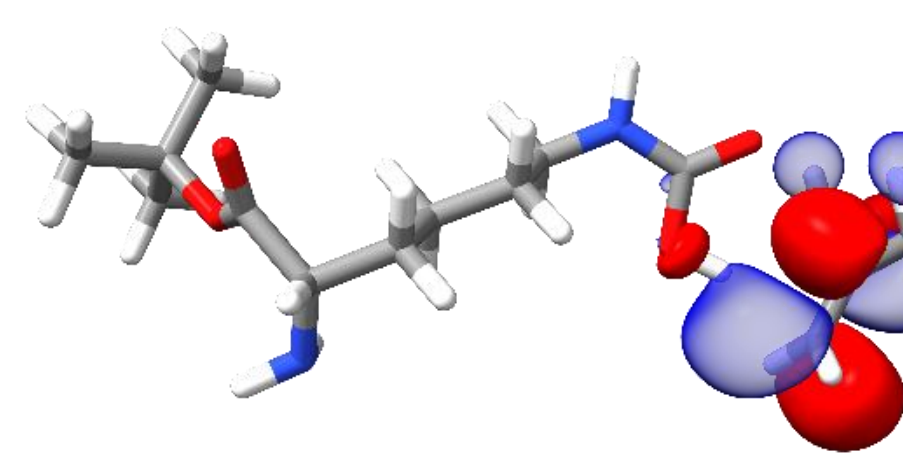

6	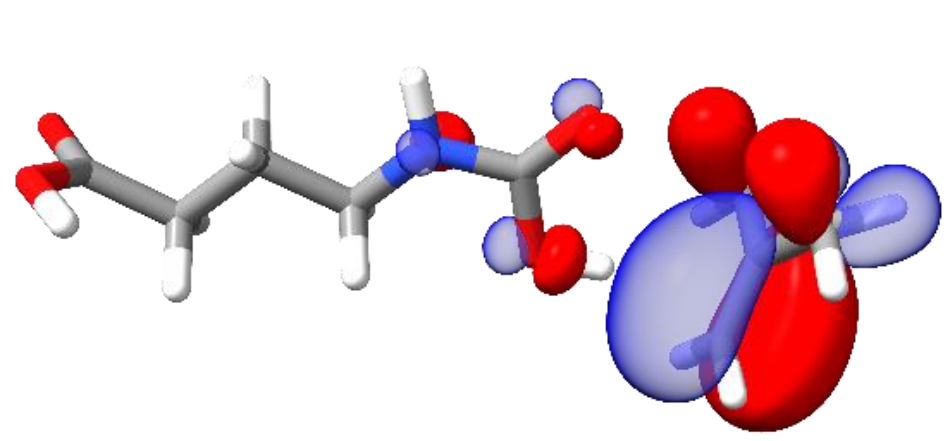	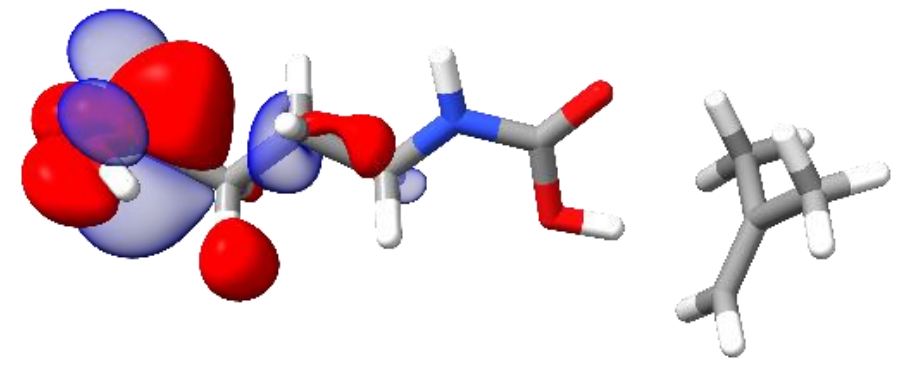
7	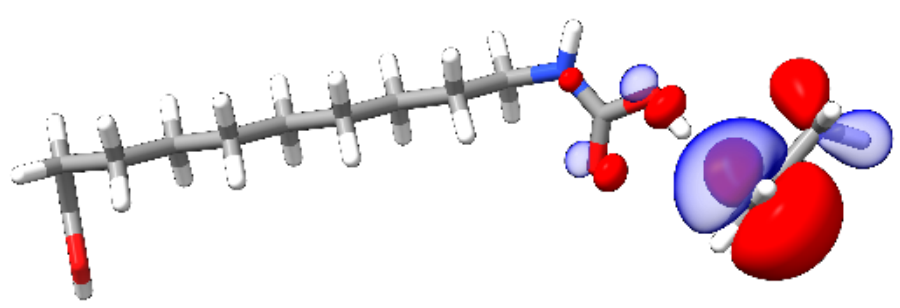	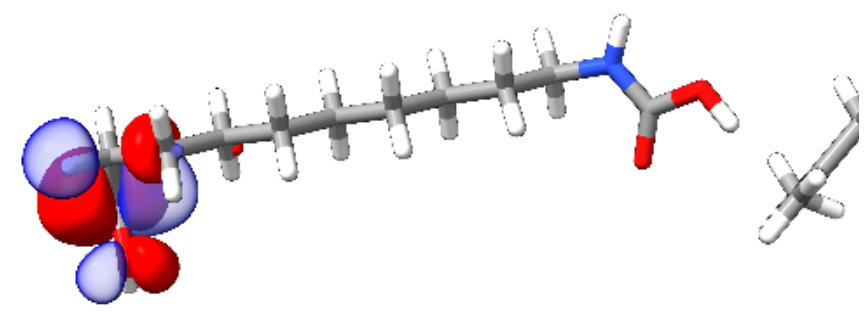
8	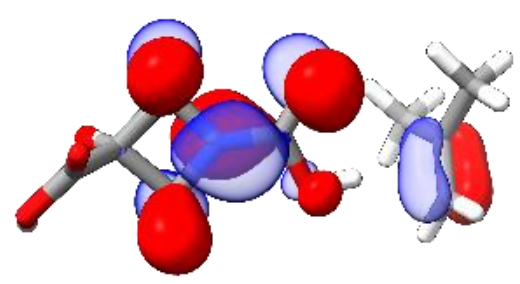	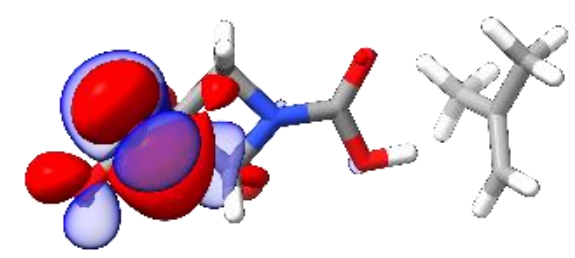
9	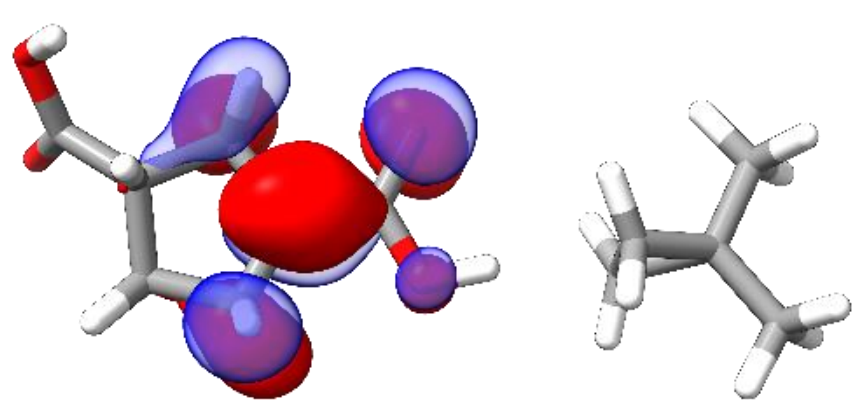	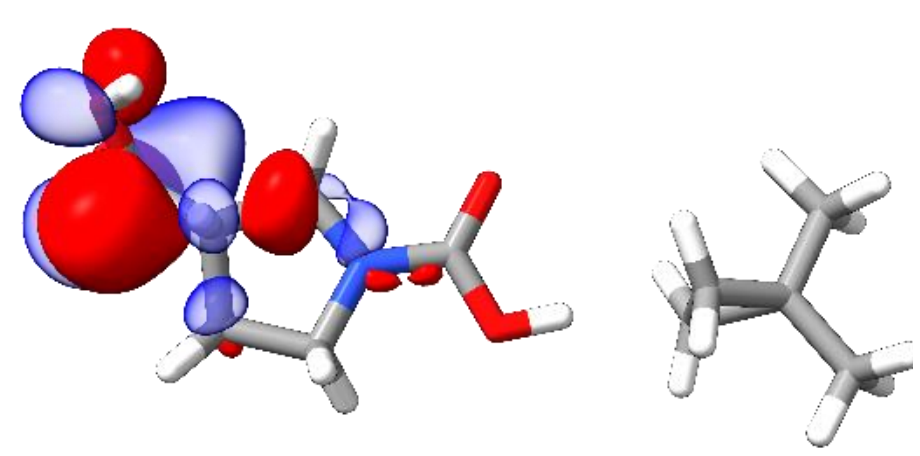
10	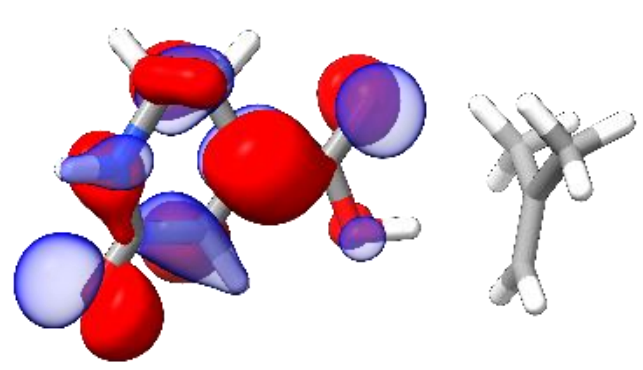	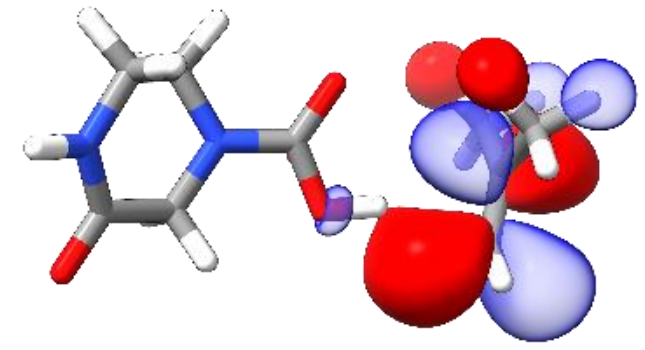

11 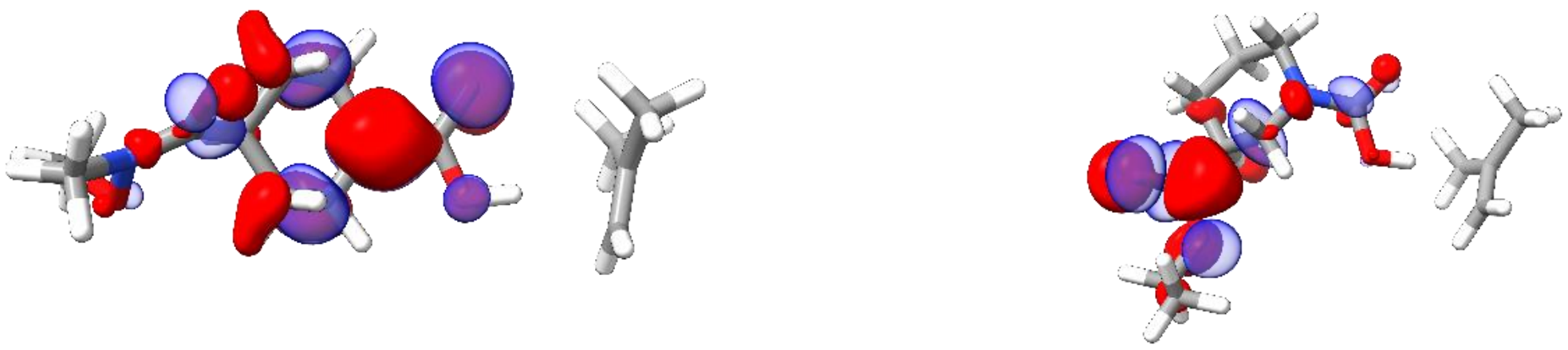

12 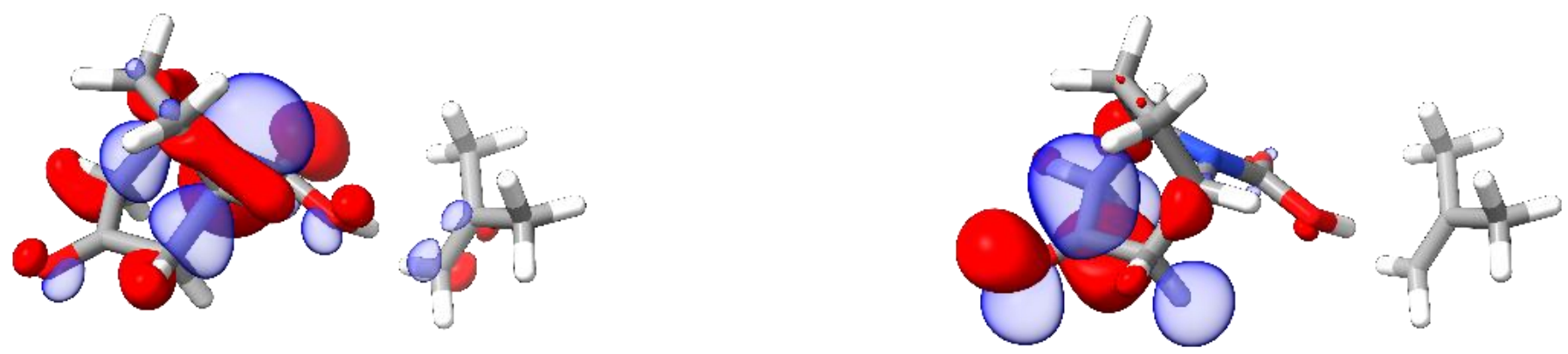

13 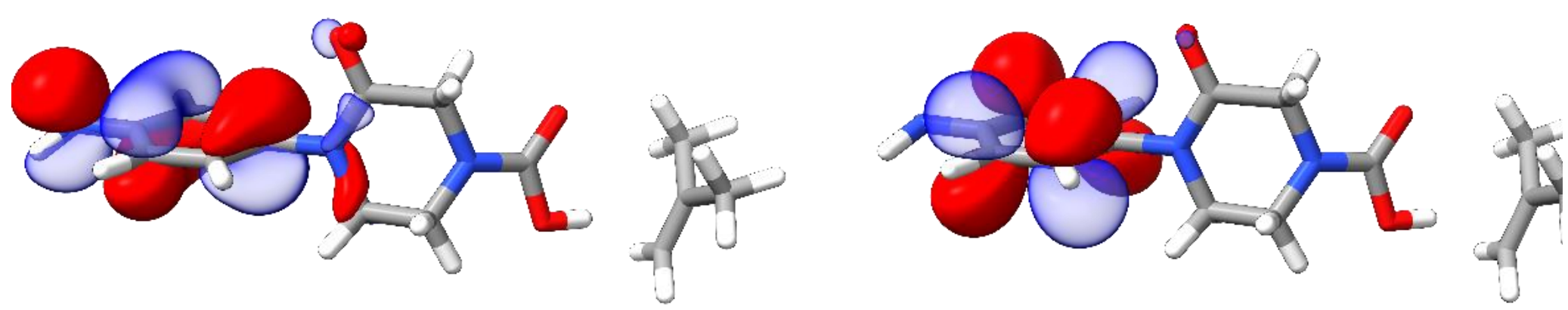

14 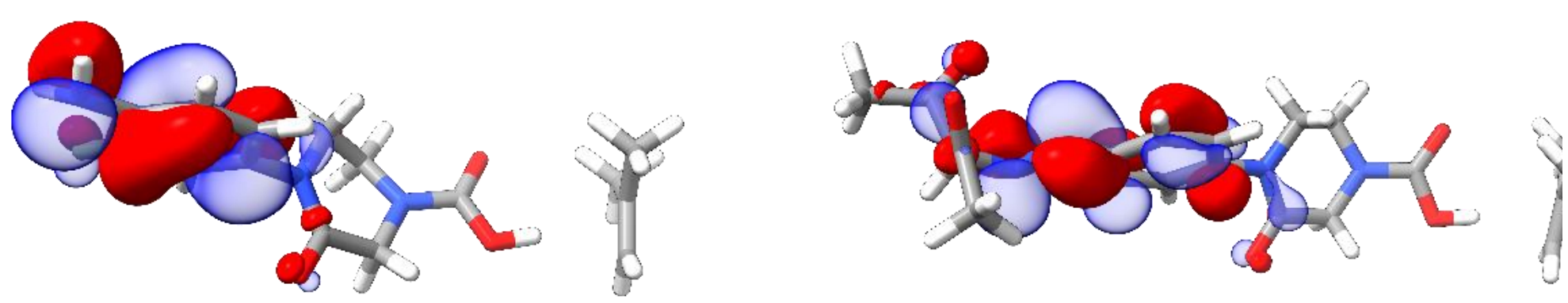

15 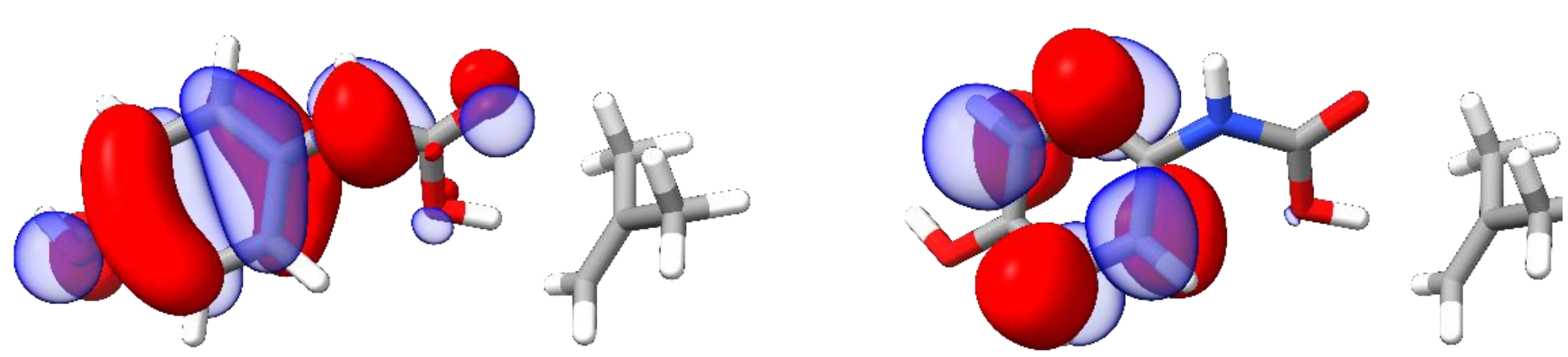

16 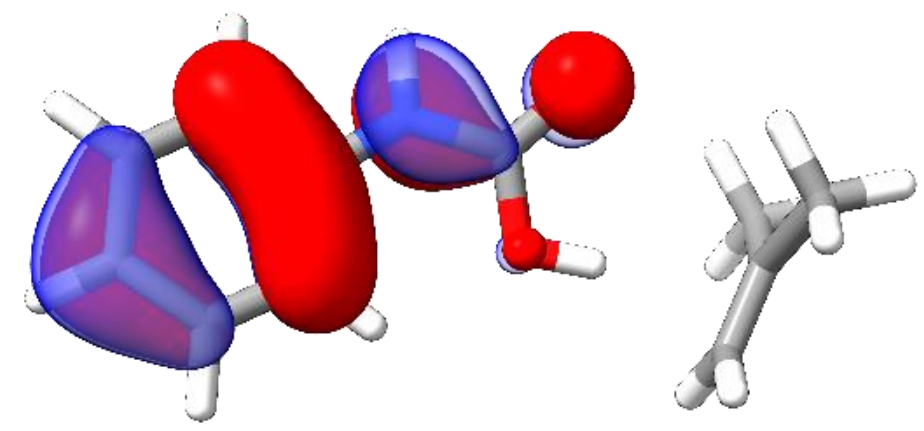 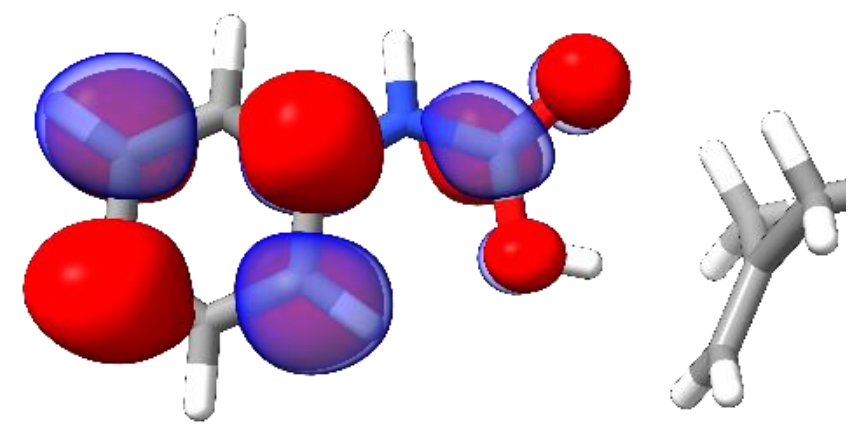

17 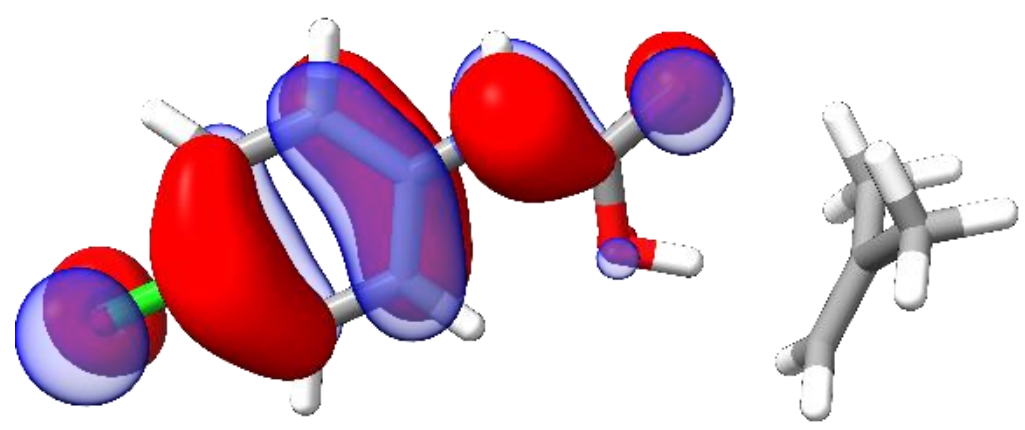 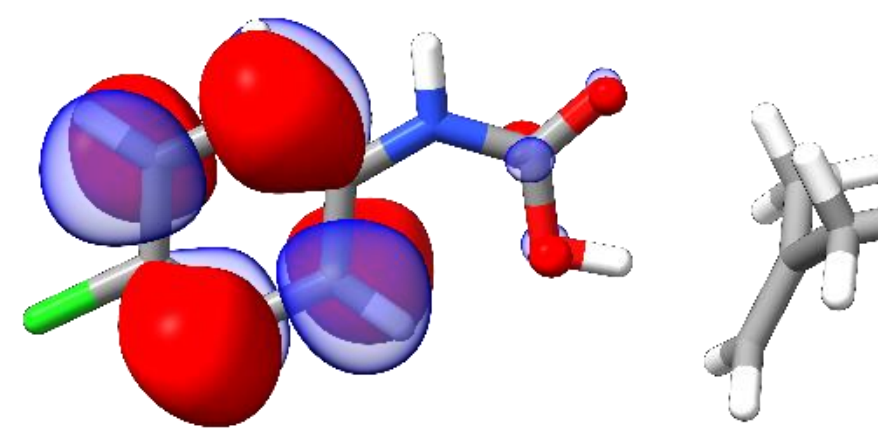

18 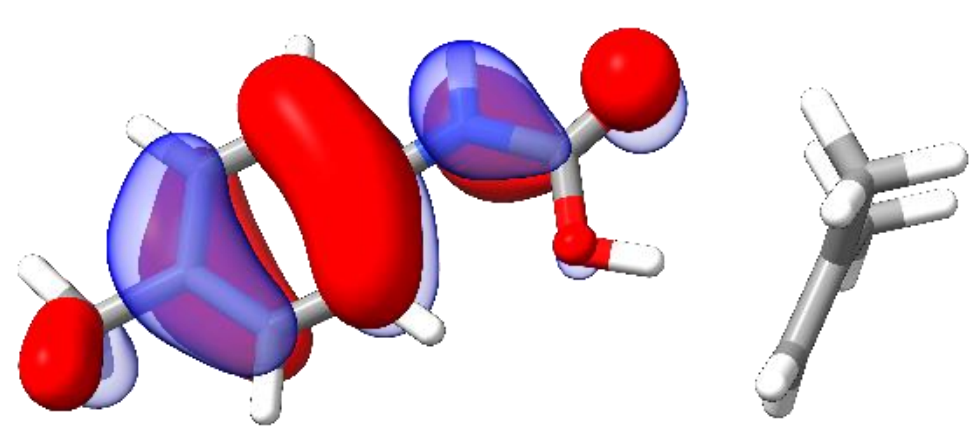 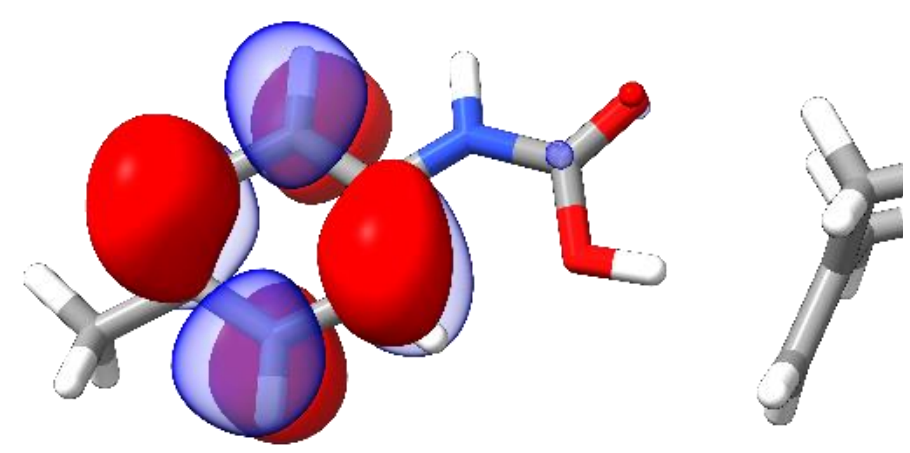

19 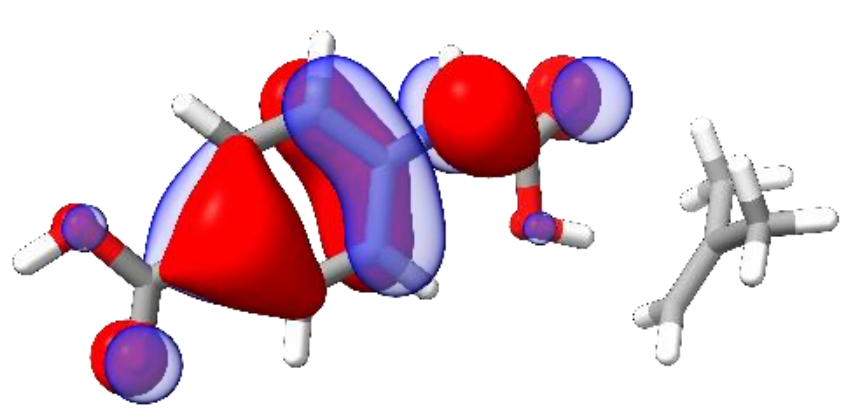 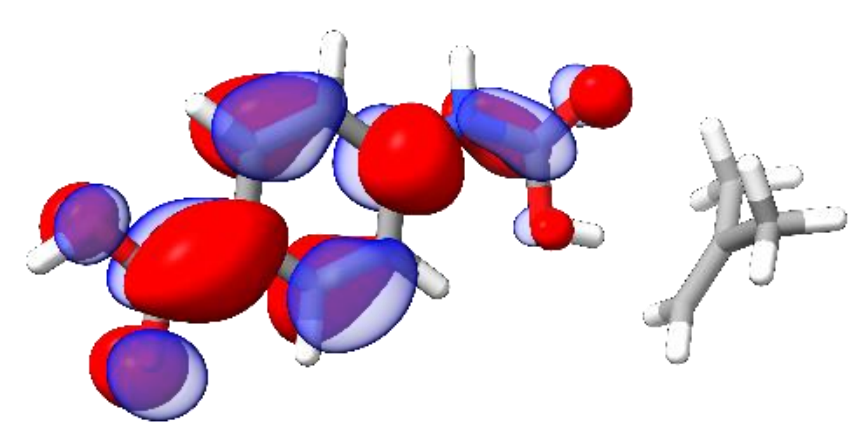

20 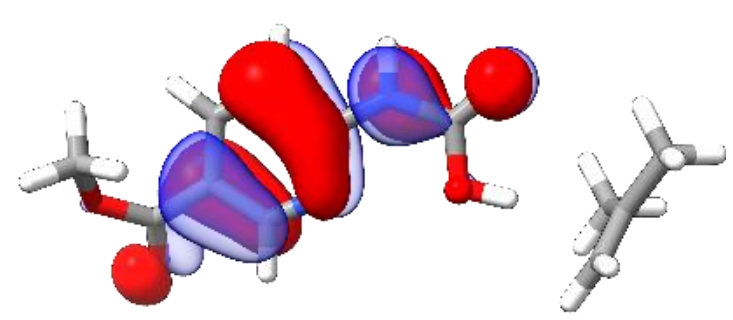 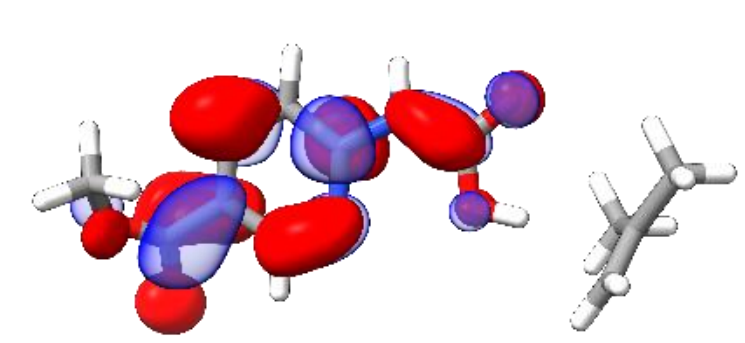

21

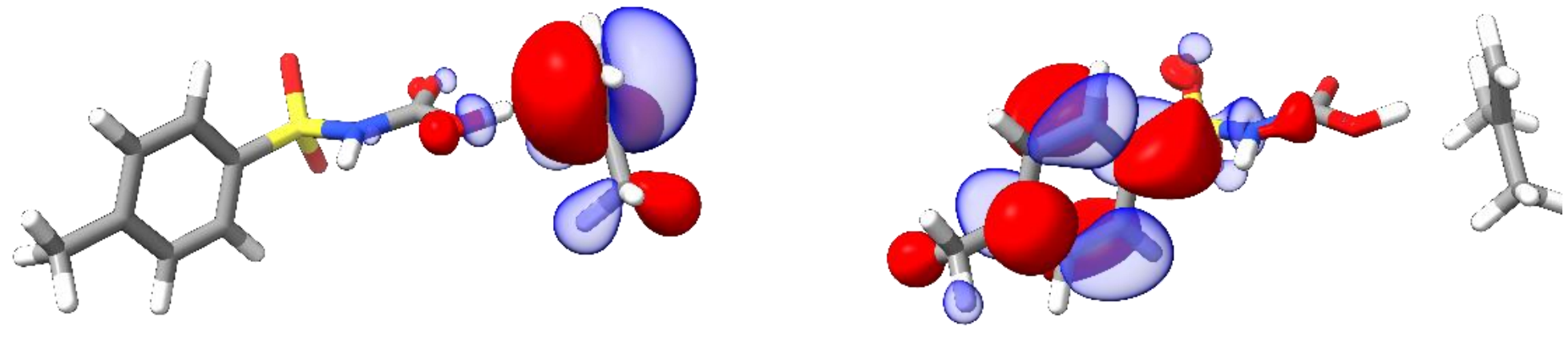

22

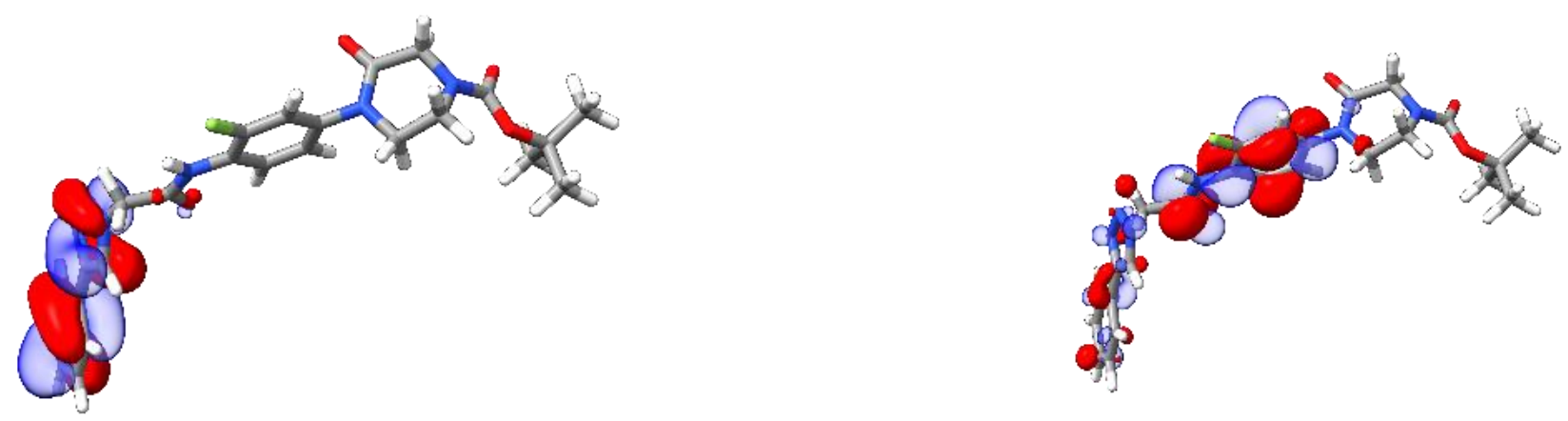

23

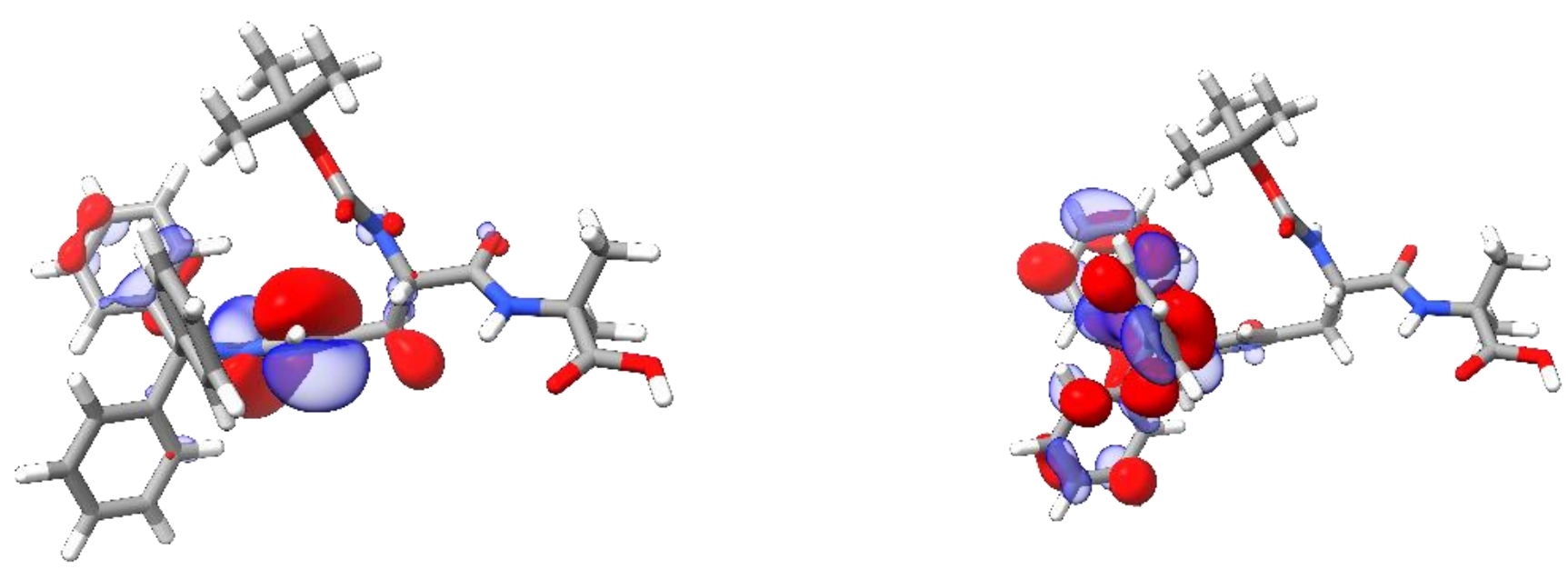

Table 5. Global thermochemistry (P-R)

Entry	dG kcal/mol	dH kcal/mol	TdS kcal/mol
**1**	4,27	7,36	-3,09
**2**	4,91	7,50	-2,59
**3**	4,50	8,63	-4,13
**4**	5,93	5,51	0,42
**5**	6,43	7,08	-0,65
**6**	4,17	7,82	-3,66
**7**	3,42	5,70	-2,28
**8**	3,50	6,34	-2,84
**9**	12,89	15,63	-2,75
**10**	4,62	5,97	-1,35
**11**	3,50	6,34	-2,84
**12**	3,71	6,73	-3,03
**13**	4,02	6,62	-2,60
**14**	3,98	6,64	-2,66
**15**	4,50	8,63	-4,13
**16**	6,84	7,91	-1,07
**17**	6,95	8,00	-1,05
**18**	7,36	7,24	0,12
**19**	4,67	7,23	-2,56
**20**	5,87	7,42	-1,55
**21**	3,91	8,06	-4,16
**22**	4,55	6,89	-2,35
**23**	2,52	5,74	-3,21

Table 6. Path summary for the NEB-TS calculation obtained from ORCA (3)

Image	dE(kcal/mol)	max(|Fp|)	RMS(Fp)
0	0.00	0.00007	0.00001
1	6.22	0.00310	0.00079
2	16.78	0.00284	0.00079
3	27.44	0.00184	0.00058
4	32.26	0.00200	0.00054
5 (TS)	32.34	0.00107	0.00016
6	28.57	0.00677	0.00176
7	14.96	0.01031	0.00169
8	10.34	0.00267	0.00061
9	8.83	0.00022	0.00007

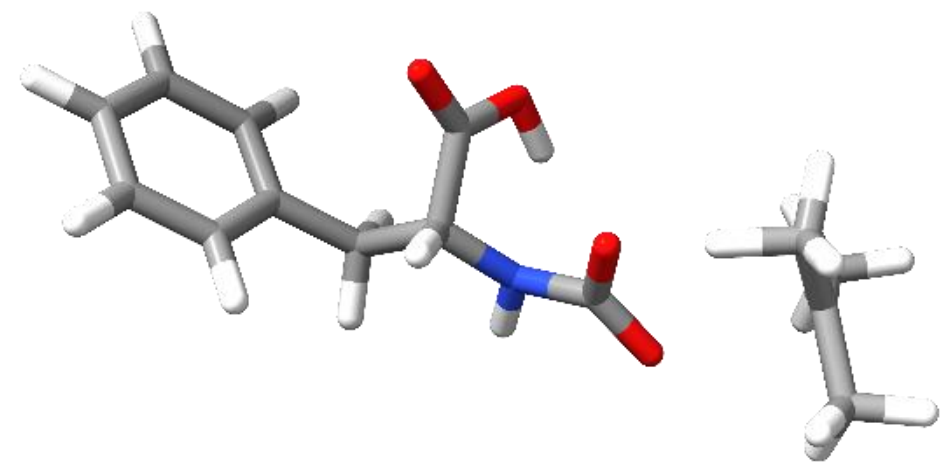

Table 7. Path summary for the NEB-TS calculation obtained from ORCA (**8**)

Image	dE(kcal/mol)	max(|Fp|)	RMS(Fp)
0	0.00	0.00011	0.00003
1	5.69	0.00595	0.00163
2	14.56	0.01006	0.00268
3	25.38	0.00916	0.00245
4	32.02	0.00360	0.00105
5 (TS)	33.64	0.00047	0.00008
6	17.28	0.00587	0.00191
7	11.85	0.00522	0.00157
8	8.83	0.00862	0.00128
9	7.62	0.00028	0.00007

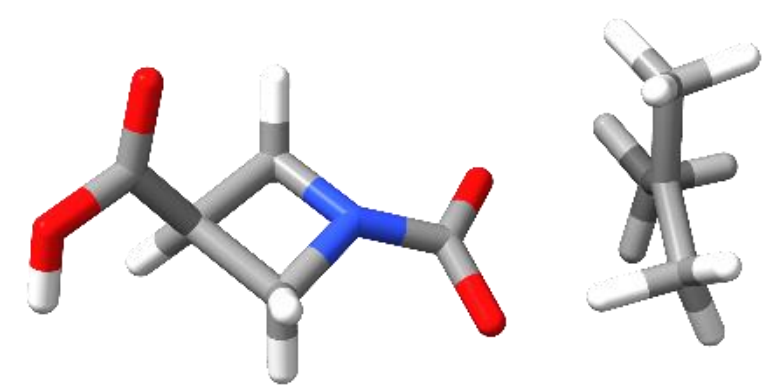

Table 8. Path summary for the NEB-TS calculation obtained from ORCA (**9**)

Image	dE(kcal/mol)	max(|Fp|)	RMS(Fp)
0	0.00	0.00011	0.00003
1	13.21	0.00237	0.00075
2	33.93	0.00492	0.00106
3	52.85	0.00202	0.00054
4	62.60	0.00111	0.00033
5 (TS)	61.38	0.00020	0.00005
6	58.20	0.00204	0.00063
7	48.75	0.00222	0.00068
8	21.80	0.00197	0.00053
9	16.43	0.00012	0.00003

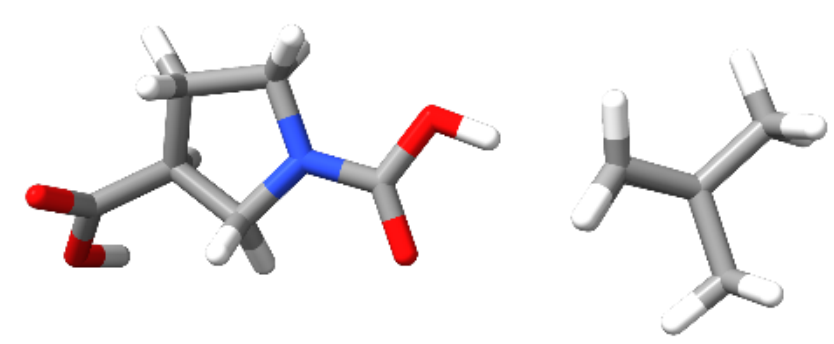

Table 9. Path summary for the NEB-TS calculation obtained from ORCA (**10**)

Image	dE(kcal/mol)	max(|Fp|)	RMS(Fp)
0	0	0,00008	0,00001
1	15,25	0,0017	0,0005
2	38,07	0,0016	0,00036
3 (TS)	33,37	0,0003	0,00009
4	40,49	0,00122	0,0004
5	37,03	0,0034	0,00066
6	11,71	0,00255	0,00065
7	8,61	0,00094	0,00037
8	7,93	0,00041	0,00015
9	7,4	0,00025	0,00007

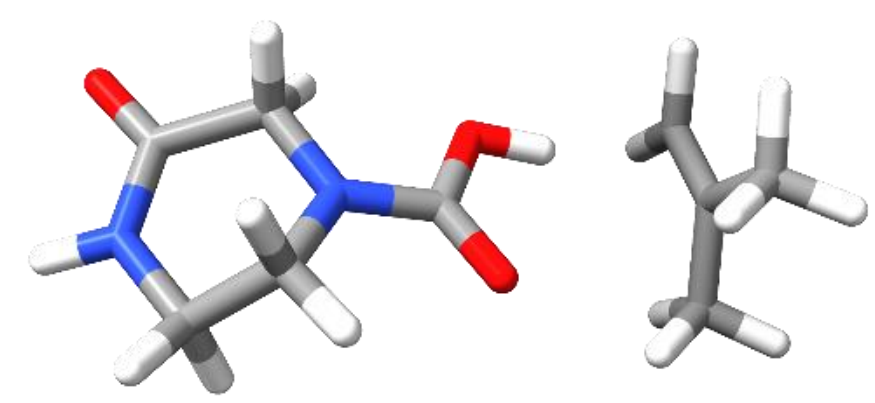

Table 10. Path summary for the NEB-TS calculation obtained from ORCA (**11**)

Image	dE(kcal/mol)	max(|Fp|)	RMS(Fp)
0	0.00	0.00012	0.00003
1	6.85	0.00114	0.00038
2	18.16	0.00252	0.00049
3	29.29	0.00139	0.00031
4	34.59	0.00120	0.00029
5 (TS)	34.23	0.00240	0.00037
6	26.20	0.00278	0.00056
7	13.02	0.00162	0.00035
8	8.96	0.00075	0.00022
9	7.13	0.00032	0.00007

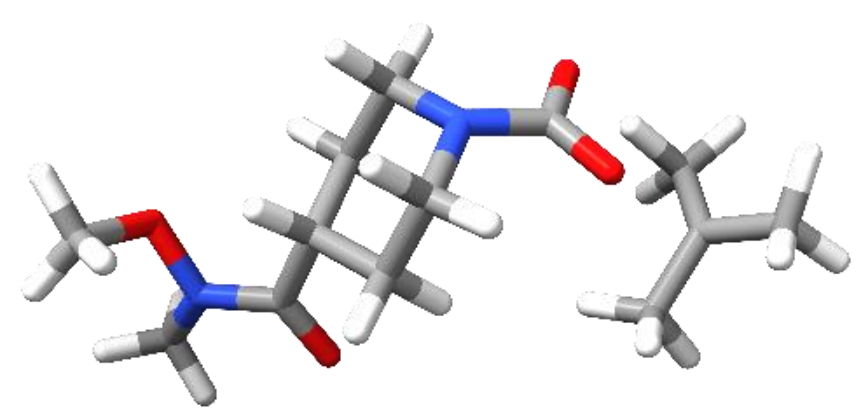

Table 11. Path summary for the NEB-TS calculation obtained from ORCA (**13**)

Image	dE(kcal/mol)	max(|Fp|)	RMS(Fp)
0	0	0,00017	0,00003
1	8,08	0,00362	0,00069
2	20,17	0,00342	0,00069
3	31,65	0,0021	0,00056
4	35,09	0,00184	0,00046
5 (TS)	33,33	0,00036	0,00005
6	27,77	0,00585	0,00157
7	10,97	0,00663	0,0012
8	7,91	0,00603	0,00094
9	7,38	0,00031	0,00008

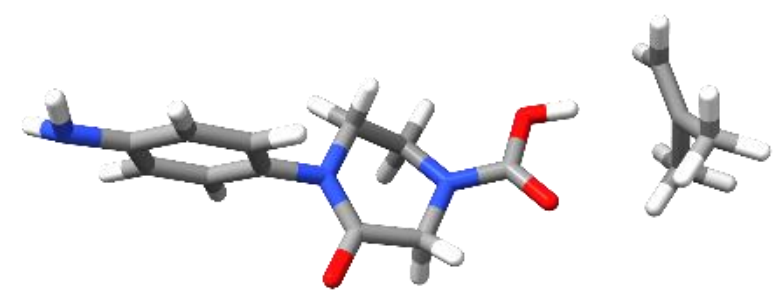

Table 12. Path summary for the NEB-TS calculation obtained from ORCA (**14**)

Image	dE(kcal/mol)	max(|Fp|)	RMS(Fp)
0	0.00	0.00006	0.00002
1	5.49	0.00603	0.00088
2	14.95	0.00256	0.00061
3	26.12	0.00303	0.00063
4	32.79	0.00245	0.00052
5	34.90	0.00190	0.00047
6 (TS)	33.38	0.00016	0.00004
7	22.33	0.00482	0.00125
8	9.61	0.00212	0.00051
9	7.48	0.00022	0.00007

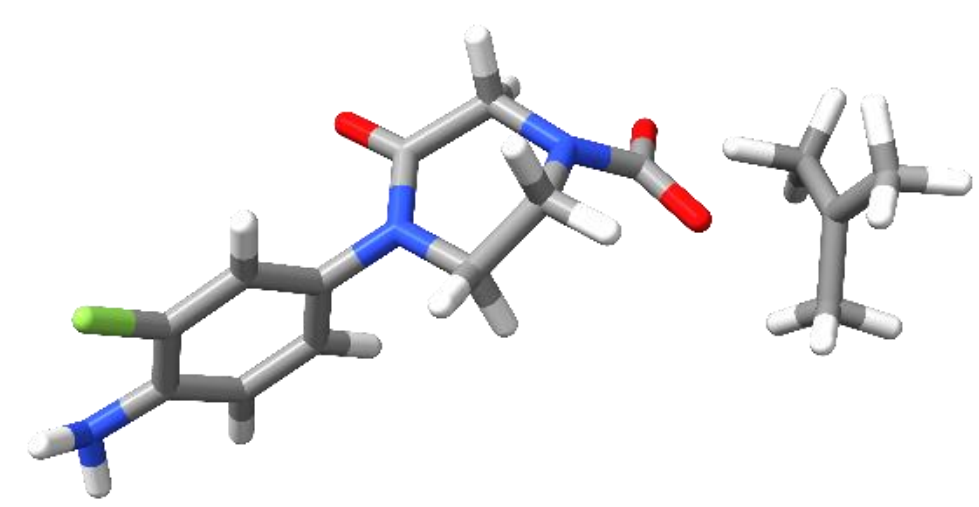

Table 13. Path summary for the NEB-TS calculation obtained from ORCA (**15**)

Image	dE(kcal/mol)	max(|Fp|)	RMS(Fp)
0	0.00	0.00014	0.00004
1	4.71	0.00673	0.00137
2	14.69	0.00520	0.00167
3	26.70	0.00256	0.00104
4	33.38	0.00232	0.00076
5 (TS)	33.70	0.00112	0.00016
6	24.81	0.01368	0.00271
7	13.48	0.00500	0.00122
8	9.93	0.00228	0.00054
9	8.81	0.00017	0.00005

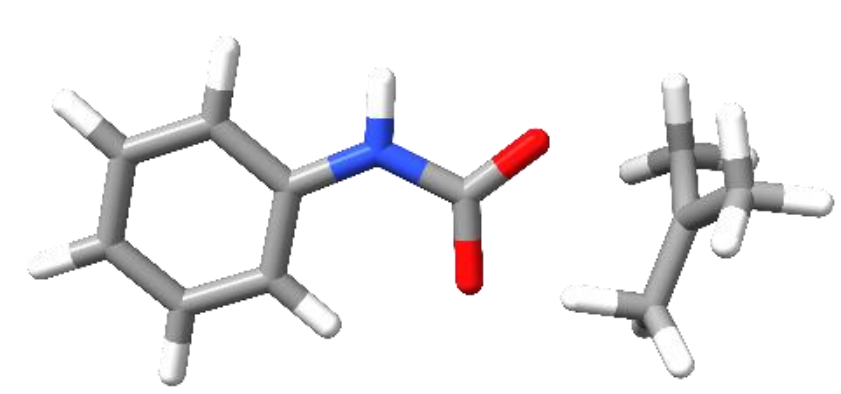

Table 14. Path summary for the NEB-TS calculation obtained from ORCA (**16**)

Image	dE(kcal/mol)	max(|Fp|)	RMS(Fp)
0	0.00	0.00017	0.00003
1	6.80	0.01085	0.00210
2	18.70	0.00538	0.00107
3	27.91	0.00290	0.00071
4	32.87	0.00245	0.00074
5 (TS)	33.18	0.00036	0.00008
6	34.39	0.00255	0.00057
7	25.36	0.00125	0.00045
8	11.54	0.00294	0.00078
9	9.22	0.00017	0.00006

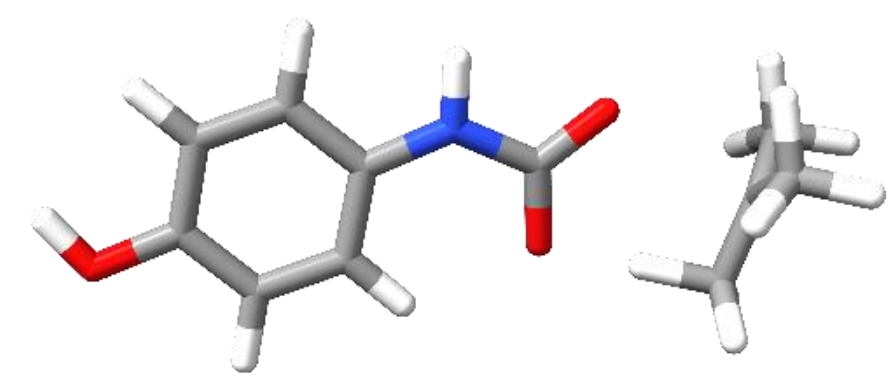

Table 15. Path summary for the NEB-TS calculation obtained from ORCA (**17**)

Image	dE(kcal/mol)	max(|Fp|)	RMS(Fp)
0	0.00	0.00017	0.00005
1	6.35	0.00554	0.00098
2	17.90	0.00306	0.00067
3	27.27	0.00283	0.00065
4	31.96	0.00288	0.00065
5	33.62	0.00242	0.00055
6 (TS)	32.82	0.00017	0.00004
7	24.89	0.00451	0.00110
8	11.64	0.00601	0.00119
9	9.32	0.00018	0.00005

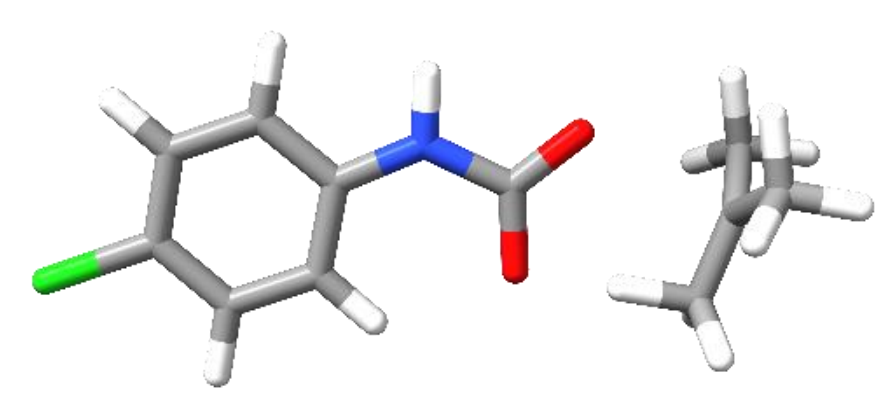

Table 16. Path summary for the NEB-TS calculation obtained from ORCA (**18**)

Image	dE(kcal/mol)	max(|Fp|)	RMS(Fp)
0	0.00	0.00021	0.00005
1	4.85	0.00466	0.00080
2	16.90	0.00328	0.00085
3	27.56	0.00290	0.00070
4	32.62	0.00253	0.00067
5	34.41	0.00212	0.00051
6 (TS)	33.47	0.00016	0.00004
7	25.45	0.00401	0.00133
8	11.64	0.00143	0.00051
9	9.17	0.00015	0.00004

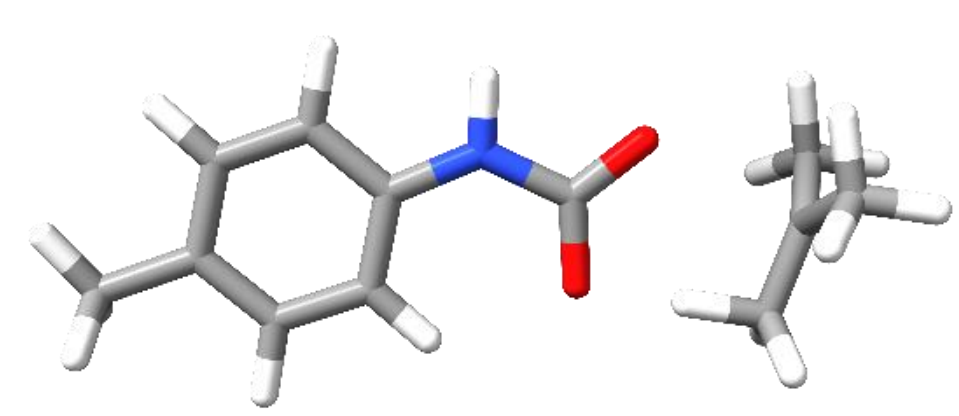

Table 17. Path summary for the NEB-TS calculation obtained from ORCA (**20**)

Image	dE(kcal/mol)	max(|Fp|)	RMS(Fp)
0	0.00	0.00008	0.00002
1	10.74	0.00223	0.00060
2	23.79	0.00175	0.00049
3	30.85	0.00138	0.00036
4	33.94	0.00147	0.00042
5 (TS)	32.04	0.00071	0.00010
6	33.21	0.00331	0.00086
7	17.47	0.00363	0.00078
8	11.01	0.00111	0.00036
9	8.83	0.00015	0.00005

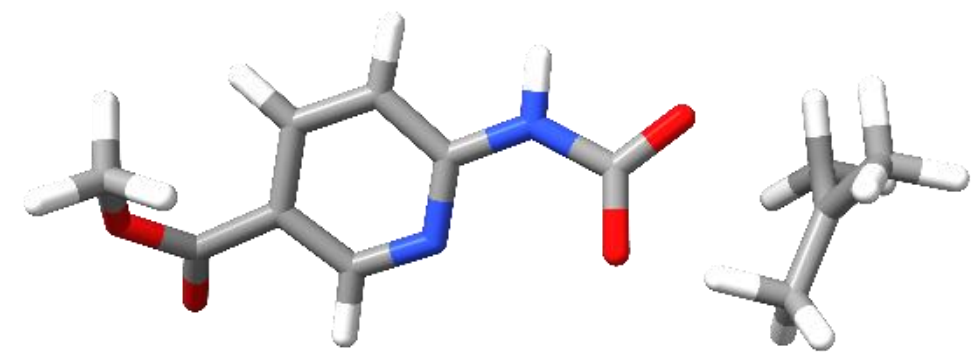

Table 18. Path summary for the NEB-TS calculation obtained from ORCA (**22**)

Image	dE(kcal/mol)	max(|Fp|)	RMS(Fp)
0	0	0,00008	0,00002
1	10,21	0,00224	0,00045
2	27,83	0,00151	0,00024
3 (TS)	33,24	0,00012	0,00002
4	28,56	0,00312	0,00057
5	13,84	0,00142	0,0003
6	10	0,00092	0,0002
7	8,86	0,00061	0,00013
8	8	0,00024	0,00007
9	7,64	0,00024	0,00007

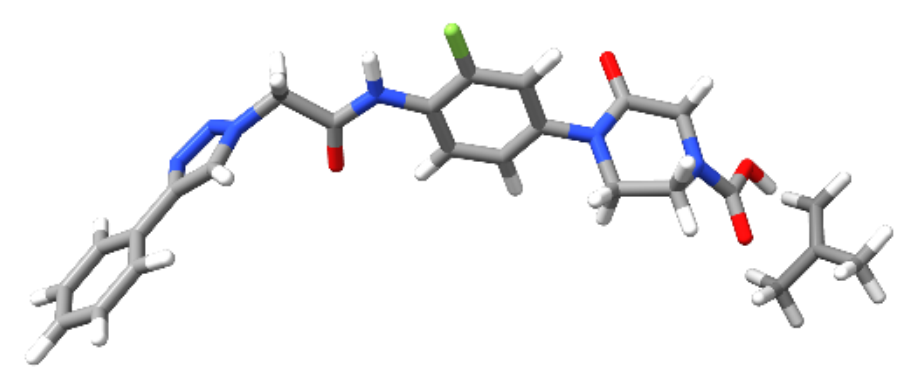

References

1 A. A. Vieira, F. R. Bryk, G. Conte, A. J. Bortoluzzi and H. Gallardo, *Tetrahedron Lett.*, 2009, **50**, 905–908.

2 A. Toró, P. Nowak and P. Deslongchamps, *J. Am. Chem. Soc.*, 2000, **122**, 4526–4527.

3 P. Wienecke and A. J. Minnaard, *J. Org. Chem.*, 2025, **90**, 3512–3518.

4 F. Neese, *WIREs Comput. Mol. Sci.*, 2012, **2**, 73–78.

5 F. Neese, *WIREs Comput. Mol. Sci.*, DOI:10.1002/wcms.1606.

6 M. D. Hanwell, D. E. Curtis, D. C. Lonie, T. Vandermeersch, E. Zurek and G. R. Hutchison, *J. Cheminform.*, 2012, **4**, 17.

7 E. Caldeweyher, J.-M. Mewes, S. Ehlert and S. Grimme, *Phys. Chem. Chem. Phys.*, 2020, **22**, 8499–8512.
